# Caloric restriction decelerates premature aging and cognitive decline in mice with deficient DNA repair

**DOI:** 10.1038/s42003-026-10182-3

**Published:** 2026-05-08

**Authors:** Chris Z. Wei, Yejie Shi, Wenting Zhang, Sulaiman H. Hassan, Ruyu Shi, Hongjian Pu, Hongfeng Mu, R. Anne Stetler, Rehana K. Leak, Jun Chen

**Affiliations:** 1 Geriatric Research, Education and Clinical Center, Veterans Affairs Pittsburgh Health Care System, Pittsburgh, PA USA; 2 Center for Cerebrovascular Disease Research and Department of Neurology, University of Pittsburgh, Pittsburgh, PA USA; 3 Department of Human Genetics, School of Public Health, University of Pittsburgh, Pittsburgh, PA USA; 4 Graduate School of Pharmaceutical Sciences, School of Pharmacy, Duquesne University, Pittsburgh, PA USA

**Keywords:** Cognitive ageing, Neural ageing

## Abstract

Accumulation of DNA damage, particularly oxidative DNA damage, is a major molecular driver of senescence and aging. The enzyme apurinic/apyrimidinic endonuclease-1 (Apex1) is essential for base-excision repair, but its role in protecting the brain from age-related deterioration remains unclear. Here we show that conditional knockout (cKO) of *Apex1* in forebrain neurons causes early and progressive cognitive impairment in mice. *Apex1* cKO mice display deficits in spatial learning and memory (8-12 weeks), alongside reduced synaptic proteins, altered neuronal morphology, and impaired long-term potentiation at 48 weeks. We further show that a 30% caloric restriction (CR) regimen at 8-48 weeks markedly attenuates these premature aging features and improves cognitive outcomes in *Apex1* cKO mice. These findings confirm *Apex1* as a critical genomic maintenance factor in the aging brain and highlight the *Apex1* cKO model as a valuable tool for studying endogenous defenses and dietary interventions against aging.

## Abstract (web summary)

Caloric restriction mitigates cognitive decline and synaptic deficits in a forebrain-specific *Apex1* knockout mouse, revealing a key role for DNA base-excision repair in protecting the aging brain from neurodegenerative changes.

## Introduction

DNA damage accumulates inexorably over life at the organismal, tissue, cellular and molecular levels^1^, serving as a powerful driving force for aging^2–4^. If left unrepaired, the accumulation of DNA damage hinders DNA replication and transcription, and promotes double-strand DNA breaks^5^. DNA damage is the result of active or passive molecular processes^6^. Active DNA damage is characterized by irreversible cellular suicide programs, whereas passive DNA damage may be elicited by reactive oxygen species (ROS). Passive DNA damage consists of DNA-protein cross-links, apurinic/apyrimidinic (AP) sites, single-strand breaks, and 8-hydroxy-2’-deoxyguanosine (8-OHdG) formation^7^. To counteract this oxidative DNA damage, neurons can engage base-excision repair (BER) processes, in which DNA glycosylases cleave the base-sugar bond to excise damaged/mismatched bases and generate temporary AP sites. The BER enzyme apurinic/apyrimidinic endonuclease 1 (*Apex1*) then excises the AP site to initiate the next step in BER^7,8^. *Apex1* cleaves the phosphodiester backbone 5’ to the AP site, creating a 3’-OH group and a 5’-deoxyribose phosphate residue, which allows a DNA polymerase to insert new nucleotides and a DNA ligase to join the strands. If AP sites are not efficiently removed by *Apex1*, the accumulation of unrepaired damaged sites may set DNA damage responses and cellular death programs in motion^9^.

Genomic instability is one of the twelve hallmarks of aging^10^. Emerging evidence suggests that DNA repair deficiency contributes to aging, including shortened lifespan and early onsets of tremor, imbalance, and muscular weakness^11,12^. While these studies shed light on the link between genomic instability and aging, there is no mechanistic evidence supporting a palliative role for *Apex1* against aging and cognitive function per se, partly because global *Apex1* knockout leads to deficiencies in developmental neurogenesis and is lethal at the embryonic stage^13^. To address this gap, we have developed the *Apex1* conditional knockout (cKO) mouse. Using the Cre/loxP recombination system, we crossed Cre mice under the control of *Camk2a* promoter with mice that have loxP sites around exons IV and V of the *Apex1* gene, thereby generating *Camk2a*-*Cre*^+/-^;*Apex1*^flox/flox^ mice, which are normal and fertile and do not exhibit physical or behavioral abnormalities in youth, defined as 0–8 weeks of age by Tsien and colleagues^14^. The *Camk2a* promoter was chosen due to the enriched expression of CaMKIIα protein in the excitatory synapses of the forebrain, including in CA1 and dentate gyrus neurons of the hippocampus^15,16^. In a previous study^17^, we focused on characterizing this *Apex1* cKO model at 24 weeks (6 months) of age, demonstrating early cognitive deficits, loss of hippocampal synaptic proteins, dendritic spine shrinkage, and impaired electrophysiological function in the brain during early adulthood, supporting its utility as a model of premature brain aging. These findings established the foundation for examining DNA repair deficits in adult neurons and enabled the testing of anti-aging interventions in a genetically defined context.

In the present study, we extended the age of study to 48 weeks in order to determine if our prior observations of premature cognitive impairments at early adulthood (24 weeks) persisted into late adulthood, or if wild-type (WT) brains would be indistinguishable from *Apex1*-cKO brain at higher ages. We then explored whether caloric restriction (CR) would be effective in mitigating the consequences of *Apex1* gene deletion in adult mice. Although the exact mechanism of CR remains elusive, a reduction in food consumption by 10-30%, without causing malnutrition, is widely recognized as one of the most effective interventions in expanding lifespan and health span, and delaying age-associated diseases^18–20^. Two of the twelve hallmarks of aging, “deregulated nutrient sensing” and “mitochondrial dysfunction,” are tightly linked to metabolic alterations^21^, whereas CR improves body mass index, skin condition, liver and kidney function, and decreases the risk of cardiovascular diseases^18,21^. In addition, CR may be of benefit in neurological conditions, such as Parkinson’s and Alzheimer’s diseases, as suggested by studies conducted in yeast, worms, flies, and mice^22–25^. These observations support the rationale for our hypothesis that DNA-repair mutant mice would benefit from CR over a prolonged period. We report that *Apex1* cKO mice display an accelerated aging phenotype into late adulthood (48 weeks), including reduced hippocampal field potentials, shorter dendritic spine lengths, and lower dendritic spine densities, and that a 30% reduction in daily caloric intake reduced multiple impairments associated with aging. We observed that deletion of *Apex1* in forebrain neurons compounded the decline in cognitive performance during aging, whereas CR effectively reduced cellular damage and improved cognitive functions in *Apex1* cKO mice by 48 weeks of age.

## Results

### Caloric restriction prevents premature aging-related decline of spatial memory and exploratory activity in *Apex1* cKO mice

**Fig. 1:**
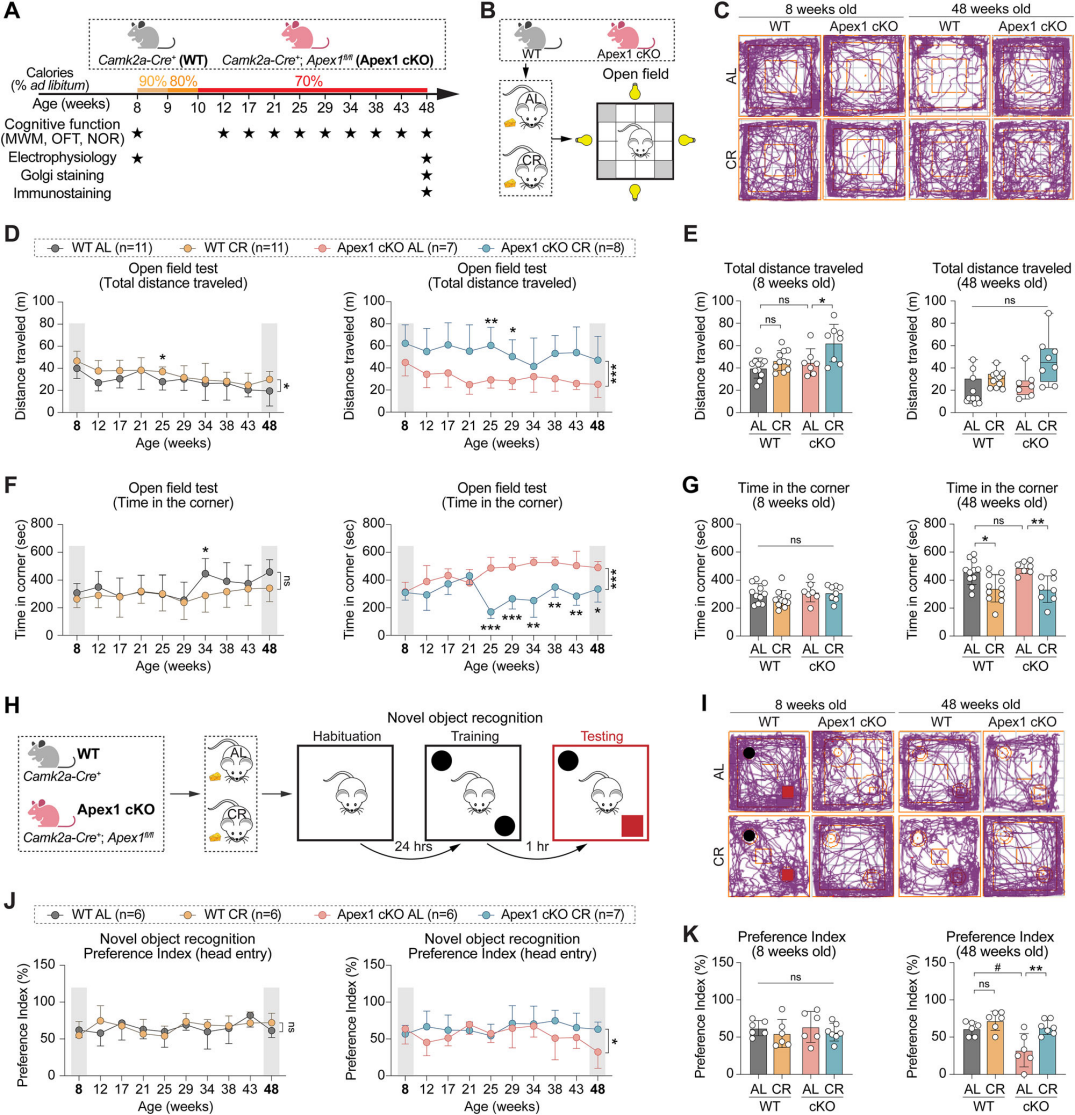
Caloric restriction boosts spontaneous motor activity, mitigates anxiety-like behaviors, and preserves novel object discrimination in *Apex1* cKO mice. **A** Timeline of caloric restriction (CR), behavior testing, immunostaining, Golgi staining, and electrophysiological analyses from week 8 to 48. **B** Experimental layout for the 10-minute-long open field test. **C** Representative locomotor activity tracks of WT *ad libitum* (AL), WT CR, *Apex1* cKO AL, and *Apex1* cKO CR mice at week 8 and 48 during the open field test. Total distance traveled (**D**) and time spent in the corner (**F**) in the open field test across 40 weeks. Gray bars in D and F indicate the time points (week 8 or 48) for multiple comparisons between WT and *Apex1* cKO groups. Total distance traveled (**E**) and time spent in the corner (**G**) of WT and *Apex1* cKO mice at week 8 and 48 in the open field test. *Apex1* cKO CR mice, but not WT CR mice, traveled significantly longer distances than their AL counterparts. WT CR mice and *Apex1* cKO CR mice also spent less time in the corners than their AL counterparts at week 48. **H** Layout of the novel object recognition (NOR) test. Arena habituation was performed 24 h before testing. **I** Representative NOR tracks of WT AL, WT CR, *Apex1* cKO AL, and *Apex1* cKO CR mice at week 8 and 48 during the NOR test. **J**–**K** Novel object preference index, as measured by % head entries towards the target object vs. both objects, over 40 weeks. Gray bars in J indicate the time points (week 8 or 48) for multiple comparisons between WT and *Apex1* cKO groups. **K** The preference index of WT and *Apex1* cKO mice was compared at week 8 and 48 in novel object recognition where *Apex1* cKO CR mice showed increased novel object exploration at week 48. Data are presented as mean ± SD or as boxplots with interquartile ranges. ns = no significant difference. # *p* < 0.05 *Apex1* AL vs. WT AL (K); *p < 0.05, **p < 0.01, ***p < 0.001 vs. AL mice of the same genotype by two-way repeated measures ANOVA/*Bonferroni* (**D**, **F**, and **J** line graphs) or the ordinary two-way ANOVA/*Bonferroni* (**E**, **G**, and **K** dot plot bar graphs). **A**, **B**, **H**: Created in BioRender. Zhang, W. (2026) https://BioRender.com/7dp5u1j.

To further establish the premature aging phenotype of the *Apex1* cKO model, we conducted an extended battery of behavioral tests spanning from 8 to 48 weeks of age, including open field, novel object recognition (NOR), and the Morris water maze, thereby building upon our prior work. In our previous study^17^, we assessed *Apex1* cKO mice up to 24 weeks in the Morris water maze and found that while spatial learning remained intact through 4-6 months, spatial memory deficits emerged by 4 months and worsened with age. The current study expands both the timeframe and behavioral repertoire, enabling a comprehensive assessment of cognitive decline. To test locomotor activity, anxiety levels and exploratory behavior, we employed the open field test^26,27^. Starting at week 8, animals under ad libitum (AL) and CR conditions were placed in the open field for 10 min once every 4 weeks over the time span of 40 weeks (Fig. 1A). *Apex1* cKO AL mice showed no difference in total distance traveled (Fig. S1A) but spent significantly more time in the corner zones and less time in the center compared to WT AL mice (Fig. S1B-C), suggesting increased anxiety-like behavior. In the NOR task, which tests recognition memory, *Apex1* cKO AL mice consistently exhibited a lower preference index than WT AL mice, based on both the number of head entries into the object zone (Fig. S1D) and the time spent exploring the novel object (Fig. S1E). These deficits were apparent across the 8 to 48-week testing window, supporting persistent recognition memory impairments. Finally, in the Morris water maze test for spatial learning and memory, *Apex1* cKO AL mice demonstrated slower escape latencies (Fig. S2A) and lower path efficiency (Fig. S2B) during the acquisition phase, indicating impaired spatial learning. During the probe trial on day 5, *Apex1* cKO AL mice spent less time in the target quadrant compared to WT AL controls (Fig. S2C), confirming spatial memory deficits. Together, these findings demonstrate that *Apex1* cKO AL mice exhibit significant impairments across multiple cognitive domains—including recognition memory, spatial memory, and stress-related behavior—beginning early in life and progressively worsening with age. The use of three independent behavioral paradigms over an extended time course provides strong evidence that *Apex1* cKO mice represent a reliable model of premature cognitive aging.

To examine the effects of CR on behavioral outcomes in WT and *Apex1* cKO mice, we implemented a stepwise caloric reduction protocol starting at 8 weeks of age, beginning with a 10% reduction in food intake and reaching a sustained 30% reduction by 10 weeks (Fig. 1A). Behavioral phenotyping was performed from 8 to 48 weeks using the open field, NOR, and Morris water maze tests. In the open field test (Fig. 1B-C), CR increased exploratory locomotion, as reflected by longer total distance traveled and altered zone exploration. The two-way ANOVA revealed a significant main effect of diet on total distance traveled at 48 weeks of age, with no significant genotype × diet interaction, indicating that the locomotor effects of CR were genotype-independent (Fig. 1D-E). Similarly, CR reduced anxiety-like behavior, as measured by decreased time spent in corner regions of the open field (Fig. 1F-G) and increased center exploration (Fig. S3A-B), across both genotypes. While individual group comparisons suggested variability in effect magnitude, these differences did not reach statistical significance after correction for multiple comparisons. Together, these results indicate that CR broadly enhances exploratory behavior and reduces anxiety-like features independent of *Apex1* genotype. In the novel object recognition test for memory (Fig. 1H-I), *Apex1* cKO CR mice showed increased novel object exploration, measured by both zone entries (Fig. 1J-K) and target zone dwell times (Fig. S3C). Novel object preference indices (calculated from head entries and zone occupancy time) demonstrated enhanced object discrimination in *Apex1* cKO CR mice compared to *Apex1* cKO AL mice over 40 weeks (Fig. 1J-K; S3C-D). No difference in novel object exploration was observed between WT AL and WT CR animals.

**Fig. 2:**
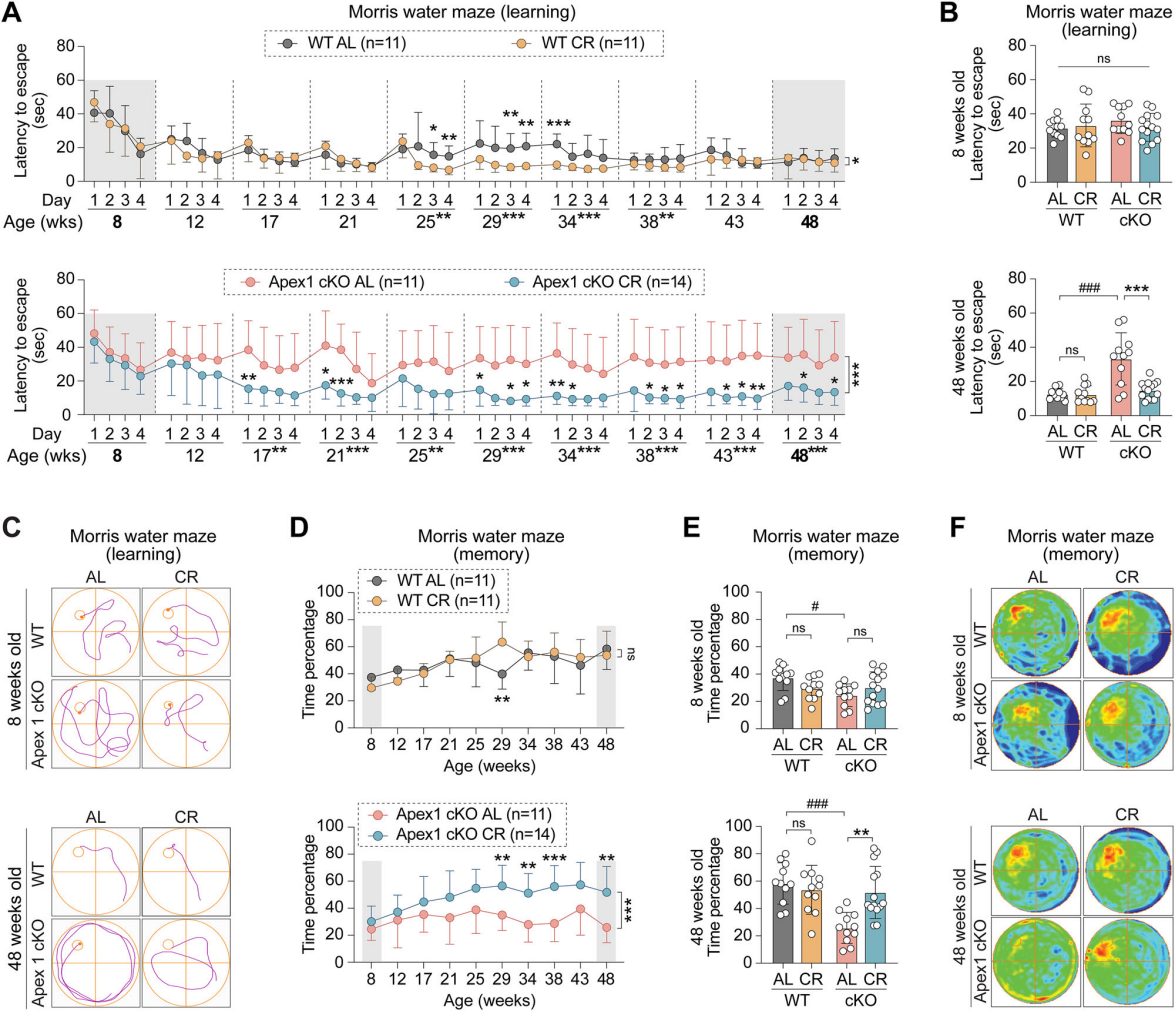
*Apex1* cKO in the mouse forebrain neurons induces premature cognitive impairments, whereas caloric restriction for 40 weeks ameliorates the decline in spatial memory. **A**, **B** Latency to reach the submerged platform across 40 weeks of AL feeding or CR. Mice were tested for 5 consecutive days each week (the first 1-4 days for the learning phase, and the 5^th^ day for memory testing). Baseline spatial learning was comparable across all groups at week 8. WT CR mice showed modest tendencies towards better performance than WT AL mice from weeks 25-38. *Apex1* cKO CR mice exhibited improved spatial learning than *Apex1* cKO AL mice from weeks 17-48. Gray bars in A indicate the time points for multiple comparisons between WT and *Apex1* cKO presented in (**B**). **C** Representative swim paths in the Morris water maze on day 4 (learning phase) at week 8 and 48. **D**–**E** The percentage time spent swimming in the target quadrant in the memory test across 40 weeks (**D**) and the testing sessions in week 8 and 48 (**E**). The memory test revealed no difference between WT groups, whereas *Apex1* cKO CR mice spent more time in the target quadrant than *Apex1* cKO AL mice from weeks 29-48. Data are presented as mean ± SD. ns = no significant difference. # *p* < 0.05, ### p < 0.001 *Apex1* AL vs. WT AL; *p < 0.05, **p < 0.01, ***p < 0.001 vs. AL mice of the same genotype by ordinary (**B**, **E**) or repeated-measures (**A**, **D**) two-way ANOVA/*Bonferroni*. **F** Representative heat map of target quadrant occupancy on day 5 (memory test) at week 8 and 48.

To assess the impact of *Apex1* expression and CR on spatial learning and memory, we used the Morris water maze test in WT and *Apex1* cKO mice with or without CR. We conducted water maze testing monthly from week 8 to 48 in WT and *Apex1* cKO mice under AL and CR conditions, respectively (Figs. 2; S4). Although this multiple testing paradigm might introduce a confound in that an ‘acclimation effect’ could emerge, the experimental design allows the assessment of habitual learning and subsequent retrieval of learned memories. The visible platform test was performed after escape latency and memory tests on day 5 of week 48, and the consistency across genotypes in locating the visible platform shows a lack of confounding visual deficits (Fig. S4C). However, *Apex1* cKO CR mice displayed faster swim speeds than *Apex1* cKO AL and WT CR animals during week 48 (Fig. S4D). Thus, to control for swim speed differences, we used both latency and path efficiency to assess spatial learning abilities (Fig. 2A-C; S4A-B). Baseline performance was comparable across all groups at week 8, indicating similar spatial learning skills across genotypes prior to CR intervention (Fig. 2B; S4B). Modest group differences were found in spatial learning performance between WT AL and WT CR mice across 40 weeks (*p* = 0.0114), as WT CR mice slightly outperformed WT AL mice from weeks 25 to 38 (Fig. 2A). *Apex1* cKO CR mice reached the submerged platform faster than *Apex1* cKO AL mice through weeks 17 to 48 (Fig. 2A, *p* < 0.0001). At later time points, escape latencies in WT mice approached a performance ceiling, potentially limiting the detectable magnitude of CR-induced improvement. In the probe trial, we found no significant difference between WT AL and WT CR mice. In contrast, *Apex1* cKO CR mice spent significantly more time in the target quadrant compared to *Apex1* cKO AL mice, from 29 to 48 weeks of age (Fig. 2C-F). These genotype-dependent effects on memory retention were further supported by analyses of latency to first entry into the target zone (Fig. S4E) and the time to cross the previous location of submerged platform (Fig. S4F).

Together, these results demonstrated that knockout of *Apex1* in neurons of the mouse forebrain impairs cognitive functions in both the novel object and Morris water maze tests, whereas prolonged CR from 8 to 48 weeks of age protected cKO mice against these cognitive deficits. WT mice did not display robust cognitive impairments up to 48 weeks of age, and the beneficial impact of CR was less apparent in WT mice. In contrast, beneficial effects of CR were pronounced in *Apex1* cKO mice, perhaps reflecting a deceleration of an aging process that is driven by impaired DNA repair. In addition, *Apex1* cKO CR mice traveled longer distances (Fig. 1E) and spent more time exploring the center region (Fig. S3B) than *Apex1* cKO AL mice at 8 weeks. Because an effect of CR was not detected in any other behavioral testing parameters in either *Apex1* cKO or WT mice at 8 weeks of age, we interpret these observations as a stress response induced by CR; in the current study, the first open field test was delayed for a few days *after* the initiation of CR. Previous studies have demonstrated that CR induces stress in mice, resulting in higher levels of corticosterone and anxiety-like behaviors^28,29^. Thus, *Apex1* cKO mice also appear to be more sensitive to CR-related stressors than WT mice.

### Caloric restriction preserves the structural properties of CA1 pyramidal neurons and suppresses DNA damage in the hippocampi of *Apex1* cKO mice

**Fig. 3:**
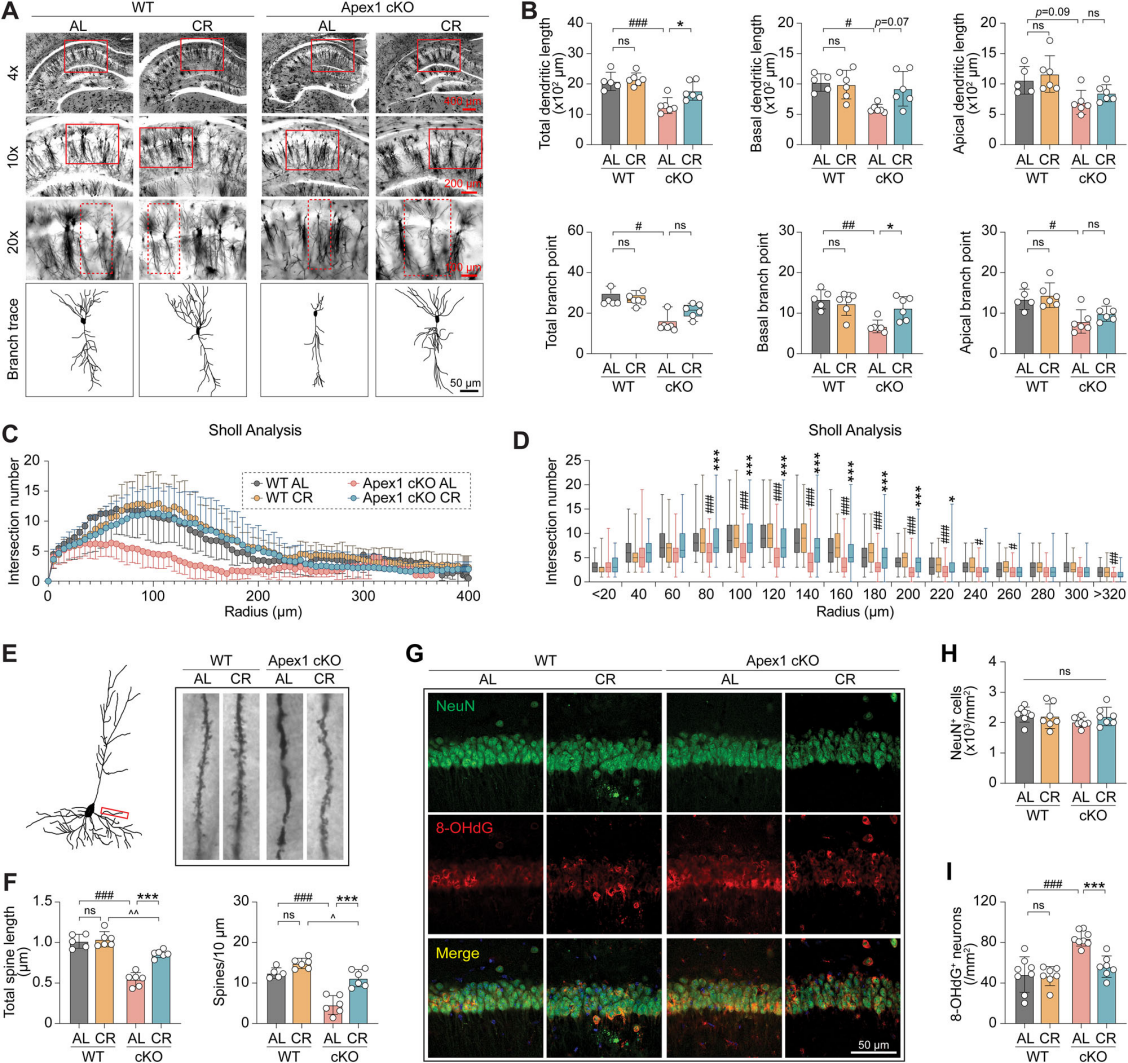
Caloric restriction protects CA1 pyramidal neurons in *Apex1* cKO mice by preserving dendritic structure and suppressing oxidative DNA damage. **A** Representative images and traces of Golgi-stained neurons of the dorsal hippocampus at 48 weeks of age. Images were photographed at 4× (cortex and hippocampus, Scale bar: 400 µm), 10× (hippocampus, Scale bar: 200 µm) or 20× (CA1, Scale bar: 100 µm) magnification. **B** Dendritic length and branch points in CA1 neurons at week 48. Each data point is the mean of 10 ROIs (*per* neuron) out of 15–20 randomly selected CA1 neurons *per* mouse. **C**, **D** Sholl analyses of hippocampal CA1 neurons (dendritic intersections at concentric circles drawn at 5 µm intervals from the soma). **E** Representative images of apical dendrites in Golgi-stained CA1 pyramidal neurons. **F** Quantification of apical CA1 dendritic spine lengths and densities. Total spine lengths and spine densities were reduced in *Apex1* cKO AL mice compared to WT AL mice. CR preserved total spine lengths and spine densities in *Apex1* cKO mice but had no effects in WT mice. Each data point is the mean of 150-200 randomly selected CA1 apical spines of each mouse. **G** Representative images of NeuN^+^ (*green*) and 8-OHdG^+^ (*red*) immunofluorescence in CA1 at week 48. Scale bar: 50 µm. **H**, **I** NeuN^+^ cell density and 8-OHdG^+^/NeuN^+^ cell counts in CA1 at week 48. Data are presented as mean ± SD or as boxplots with interquartile ranges. WT AL (*n* = 5 animals), WT CR (*n* = 6 animals), *Apex1* cKO AL (*n* = 6 animals), and *Apex1* cKO CR (*n* = 6 animals). ns = no significant difference. # *p* < 0.05, ## *p* < 0.01, ### *p* < 0.001 *Apex1* cKO AL vs. WT AL, *p < 0.05, **p < 0.01, ***p < 0.001 *Apex1* cKO CR vs. *Apex1* cKO AL, ^ *p* < 0.05, ^^ *p* < 0.01 *Apex1* cKO CR vs. WT CR by ordinary two-way ANOVA/*Bonferroni* tests for normal data distributions or Kruskal-Wallis one-way ANOVA/*Dunn* tests for non-normal data distributions.

Previously, we examined the loss of *Apex1* in adult mice at 6 months of age to assess the impact of *Apex1* in the brains of relatively young animals. Those studies identified that loss of *Apex1* was correlated with the loss of dendritic integrity in CA1 neurons compared to age-matched wildtype littermates. Here, we extended the study to 48 weeks of age and determined whether CR can mitigate the impact of loss of *Apex1* at later ages, by assessing morphological features of CA1 pyramidal neurons. *Apex1* cKO AL mice displayed severe deformation of Golgi-impregnated neuronal structures at 48 weeks of age, exhibiting decreases in total dendritic length (*p* = 0.0007), basal dendritic length (*p* = 0.0152), total branch points (*p* = 0.0122), basal branch points (*p* = 0.0016), and apical branch points (*p* = 0.0157) compared to WT AL mice (Fig. 3A-B). *Apex1* cKO CR mice showed greater total dendritic lengths (*p* = 0.0366) and basal branch points (*p* = 0.0318) and a statistical trend toward increases in basal dendritic length (*p* = 0.0701) compared to *Apex1* cKO AL mice (Fig. 3B). In contrast, no difference in dendritic morphologies was observed between WT AL and WT CR animals at 48 weeks of age. These results support a role for *Apex1* in maintaining the structural integrity of CA1 neuronal processes during aging.

We further analyzed dendritic structures in hippocampal CA1 using Sholl analysis^30^, which quantifies dendritic intersections at concentric circles drawn at 5-µm intervals from the soma. Compared to all other groups, *Apex1* cKO AL mice showed reduced dendritic intersections at intermediate to large radii (80 - >320 µm) (Fig. 3C-D). *Apex1* cKO CR mice had more intersections compared to *Apex1* cKO AL mice at intermediate radii (80–220 µm) (Fig. 3D). No difference in dendritic intersections was observed between WT AL and WT CR animals at 48 weeks of age (Fig. 3D). We also measured dendritic spine densities (the number of spines per 10 micrometers) and spine length (total spine length per micrometer) of CA1 pyramidal neurons (Fig. 3E-F). *Apex1* cKO AL mice showed lower dendritic spine densities and spine lengths (*p* < 0.0001) compared to WT AL mice (Fig. 3F), whereas CR significantly rescued both parameters (*p* < 0.0001) compared to *Apex1* cKO AL mice. No differences in dendritic spine densities and spine length were observed between WT AL and WT CR animals at 48 weeks of age (Fig. 3F).

To test if CR preserved cellular and dendritic spine integrity in hippocampal pyramidal neurons by reducing oxidative DNA damage, we first evaluated *Apex1* expression in hippocampal neurons by measuring the percentage of NeuN^+^ cells that co-expressed *Apex1*. The proportion of NeuN/APE1-double positive cells was markedly reduced in *Apex1* cKO mouse in the CA1, CA3, and cortex (Fig. S5A-D), with no difference between the *Apex1* cKO AL and CR groups. These results confirm the deletion of *Apex1* in a sizable fraction of hippocampal neurons in *Apex1* cKO mice. We subsequently evaluated the expression of DNA polymerase beta (PolB), an enzyme downstream of *Apex1* along the BER pathway, which showed no significant differences across all groups (Fig. S5E-H), suggesting no compensatory upregulation of PolB by *Apex1* cKO or CR. Taken together, these data reveal that the memory-preserving effects of CR are not mediated through upregulation of APE1 or PolB.

We further examined the impact of CR and *Apex1* loss on oxidative DNA damage by measuring 8-hydroxy-2’-deoxyguanosine (8-OHdG)^31,32^. *Apex1* cKO AL mice showed higher 8-OHdG signals in NeuN^+^ neurons in CA1 compared to WT AL mice (*p* < 0.0001); this increase was attenuated in *Apex1* cKO CR mice (*p* < 0.0001, Fig. 3G, I). Thus, CR-afforded protection against hippocampal impairment in *Apex1* cKO mice is associated with less oxidative DNA damage.

### Caloric restriction preserves long-term potentiation in *Apex1* cKO mice

Age-related decline in spatial memory is strongly associated with reduced long-term potentiation (LTP) at hippocampal Schaffer collateral-CA1 synapses^33,34^. To determine whether *Apex1* cKO impairs synaptic plasticity and if CR rescues this deficit, we assessed LTP ex vivo in acute hippocampal slices. At 8 weeks of age, both WT and *Apex1* cKO mice (without CR) exhibited comparable LTP, with no significant differences in fEPSP slopes across the 60-minute post-stimulation recording period (Fig. S6A, B). In contrast, by 48 weeks, *Apex1* cKO AL mice showed significantly reduced LTP (*p* = 0.0317) compared to WT AL mice (Fig. S6C), suggesting that loss of *Apex1* from forebrain neurons impairs synaptic plasticity over time. The aging effect on LTP was also analyzed in WT AL and *Apex1* cKO AL mice by comparing LTP between 8 and 48 weeks of age. While no difference was found between 8 and 48 weeks in WT AL mice (*p* = 0.5328), LTP was impaired in *Apex1* cKO AL mice at 48 weeks compared to 8 weeks (*p* = 0.0347), supporting the notion that *Apex1* deficiency accelerates brain aging.

**Fig. 4:**
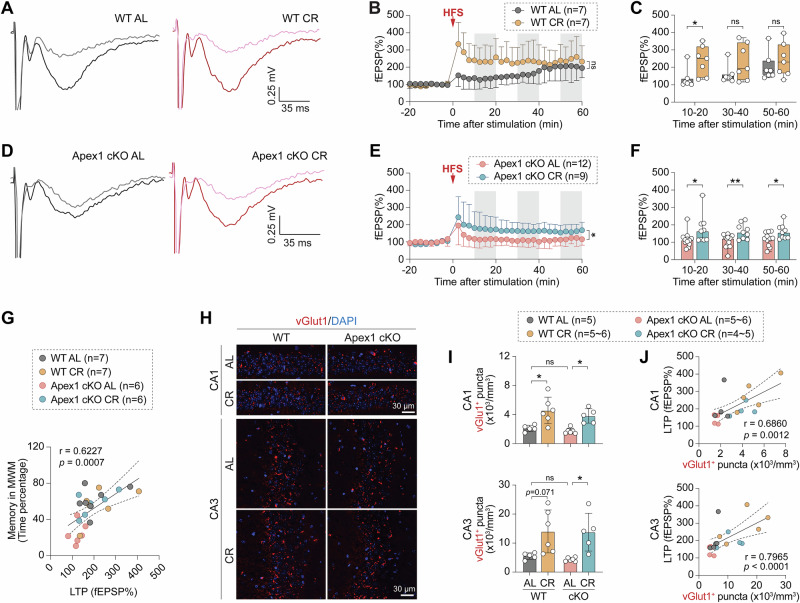
Caloric restriction restores synaptic plasticity and enhances long-term potentiation in *Apex1* cKO mice. Representative traces of evoked LTP and quantification of fEPSP slope percentages for WT AL and WT CR mice (**A**) and for *Apex1* cKO AL and *Apex1* cKO CR mice (**D**) at 48 weeks of age. **B**, **E** Cumulative data plots of fEPSP slope (normalized from baseline) before and up to 60 min after high-frequency stimulation (HFS). **C**, **F** Quantification of fEPSP slope recording at the indicated three 10 min courses (10–20 min, 30–40 min, and 50–60 min) after HFS. *n*  =  7 per WT group, *n* = 9–12 per *Apex1* cKO group. **G** Pearson’s two-tailed correlation coefficient analysis of LTP magnitude vs. spatial memory performance in the Morris water maze test at week 48. **(H)** Representative images of DAPI (*blue*), vesicular glutamate transporter 1 (vGlut1, *red*) in hippocampal CA1 and CA3 at week 48 (scale bar: 30 µm). **I** Quantification of excitatory vGlut1^+^ puncta densities from randomly selected regions of interest in hippocampal CA1 and CA3, respectively, analyzed from Z-stack confocal images. **J** Spearman’s two-tailed correlation analysis of fEPSP slope magnitude versus vGlut1^+^ puncta densities of hippocampal CA1 and CA3. *n* = 5 per WT group, *n* = 5 for *Apex1* cKO AL group, *n* = 4 for *Apex1* cKO CR group. Data are mean ± SD. ns = no significant difference. *p < 0.05, **p < 0.01 vs. indicated group by two-way repeated-measures (**B**–**F**) or two-way ordinary ANOVA/*Bonferroni* tests (**I**).

Next, we assessed the impact of CR in mice of both genotypes. In WT mice, CR had modest effects on LTP at 48 weeks: fEPSP slopes were transiently elevated in WT CR mice 10–20 min after stimulation compared to WT AL mice, but no differences were observed at later timepoints (30–40 and 50–60 min; Fig. 4A-C). In contrast, CR markedly improved LTP in *Apex1* cKO mice (Fig. 4D-E). Hippocampal slices from *Apex1* cKO CR mice exhibited significantly higher fEPSP slopes than their AL-fed counterparts across the entire recording period (10–20, 30–40, and 50–60 min post-stimulation; Fig. 4F). Notably, Pearson’s correlation coefficient analysis revealed that the magnitudes of LTP positively correlated with spatial memory performance (Morris water maze) across groups (Fig. 4G), suggesting that the ability of CR to restore synaptic plasticity may underlie its cognitive benefits in *Apex1*-deficient mice.

A reduction in presynaptic protein expression could account for an LTP deficiency^35,36^. To investigate potential mechanisms underlying the observed LTP changes, we examined the expression of synaptic markers in the hippocampus at 48 weeks. We stained for the excitatory presynaptic marker vGlut 1 (Fig. 4H) and the inhibitory marker GAD (Fig. S7). While vGlut1 expression was comparable in the CA1 and CA3 regions between WT AL mice and *Apex1* cKO AL mice (Fig. 4H-I), vGlut1 expression was elevated in CA1 of WT CR mice (*p* = 0.0102) and *Apex1* cKO CR mice (*p* = 0.0361) and CA3 of *Apex1* cKO CR mice (*p* = 0.0357), compared to corresponding AL controls (Fig. 4H-I). Furthermore, vGlut1 expression levels in CA1 and CA3 correlated positively with fEPSP slopes (Fig. 4J), thereby reinforcing the link between synaptic structure and electrophysiological function. In contrast, GAD expression levels remained unchanged in CA1 and CA3 across all groups (Fig. S7A-B). These results suggest that CR may preserve electrophysiological functions of hippocampal Schaffer collateral-CA1 synapses by elevating the expression of excitatory presynaptic proteins, including vGlut1.

### Gene expression changes in the brain with *Apex1* conditional knockout

**Fig. 5:**
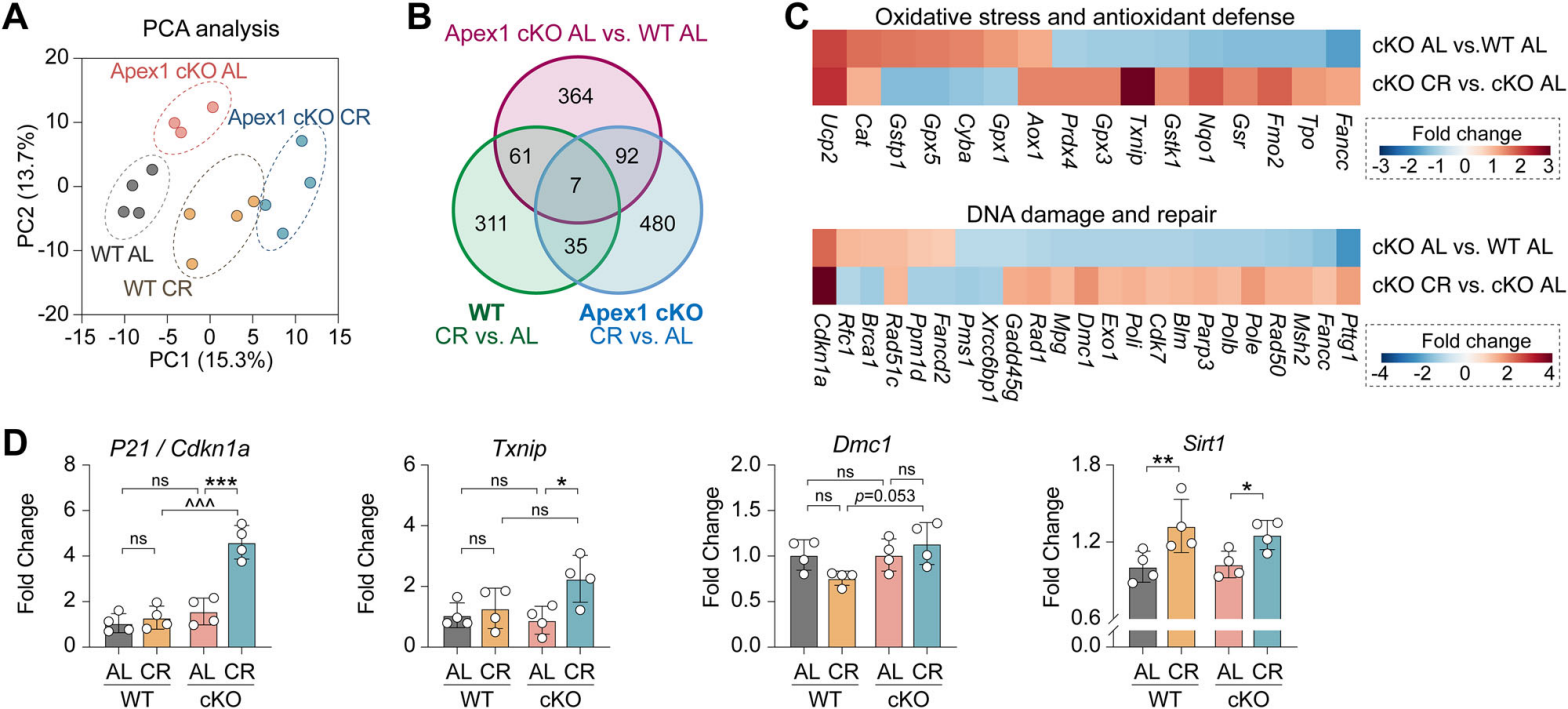
Caloric restriction alters the hippocampal transcriptomic profiles. **A** Principal component analysis (PCA) score plot for the microarray results. **B** Venn diagram of differentially expressed genes for *Apex1* cKO AL vs. WT AL (*purple*), WT CR vs. WT AL (*green*), and *Apex1* cKO CR vs. *Apex1* cKO AL (*blue*), and overlapping genes for the three clusters. **C** Heatmap of changes in the expression of DNA damage/repair and oxidative stress/antioxidant defense genes. **D** qPCR verification of *P21/Cdkn1a*, *Txnip*, *Dmc1*, and *Sirt1*, which were originally identified from the microarray experiment. PCR data are shown as mean ± SD. ns = no significant difference. *p < 0.05, **p < 0.01, ***p < 0.001, ^^^ *p* < 0.001 vs. indicated group by two-way ANOVA/*Bonferroni* tests.

We performed exploratory microarray analyses on hippocampal tissue from 48-week-old mice across four experimental groups: WT AL, WT CR, *Apex1* cKO AL, and *Apex1* cKO CR (Fig. 5A-C). Principal component analysis (PCA) revealed low within-group variability and clear transcriptional distinctions between genotypes and treatment conditions (Fig. 5A). In particular, the *Apex1* cKO AL group clustered distinctly from WT AL mice, highlighting widespread gene expression alterations due to *Apex1* deletion (Fig. 5A). Notably, CR induced a strong shift in transcriptional profiles within the *Apex1* cKO group, bringing *Apex1* cKO CR samples closer to WT CR samples in the PCA space (Fig. 5A). These observations suggest that CR partially mitigates the transcriptional abnormalities caused by Apex1 deficiency, supporting a role for CR in restoring gene expression homeostasis, even in a potentially progeroid background. To further quantify group differences, we include a Venn diagram summarizing differentially expressed genes (DEGs; *p* value < 0.05; and fold change > 1.5) across key pairwise comparisons (Fig. 5B). A total of 524 DEGs were identified between *Apex1* cKO AL and WT AL mice, with 313 genes upregulated and 211 downregulated in the *Apex1* cKO AL group relative to WT AL (Fig. 5B). In WT mice, comparison of CR and AL conditions revealed 414 DEGs, including 251 upregulated and 163 downregulated genes in the CR group (Fig. 5B). Notably, CR induced more transcriptional changes in *Apex1* cKO mice than in WT controls, with 614 DEGs identified between *Apex1* cKO CR and AL groups—308 genes were upregulated and 306 were downregulated in response to CR (Fig. 5B). Out of these genes, we examined five functional clusters relevant to aging biology: aging and cellular senescence, DNA damage and repair, inflammation, oxidative stress, and synaptic plasticity. Within these categories, *Apex1* cKO AL mice demonstrated an upregulation of antioxidant defenses genes compared to WT AL mice at 48 weeks of age, including *Axo1, Cat*, *Gpx1*, *Gpx5*, and *Ucp2* (Fig. 5C), consistent with compensatory responses to oxidative stress. CR enhanced the expression of additional antioxidant genes in *Apex1* cKO mice, including *Txnip, Fmo2*, and *Nqo1*, which may underlie the observed reduction in oxidative DNA damage. CR also upregulated key DNA repair genes (*Fancc, Dmc1, Rad50, Blm*, and *Parp3;* Fig. 5C), suggesting that CR boosted the molecular machinery that bypasses BER.

To validate selected DEGs from the microarray, we performed RT-PCR on representative genes (Fig. 5D). Notably, p*21/Cdkn1a* was upregulated in *Apex1* cKO CR mice compared to both *Apex1* cKO AL and WT CR mice. Although p*21/Cdkn1a* is known for its pro-senescence properties, it is a pleiotropic gene that assists DNA repair by inducing cell cycle arrest and may be beneficial for genome maintenance in the aging brain. As expected, CR also upregulated *Sirt1*, an anti-senescence and neuroprotective gene, in both *Apex1* cKO and WT mice (Fig. 5D). More modest patterns of upregulation were seen for *Dmc1* and *Txnip*. Together, these findings indicate that CR induces transcriptional programs related to DNA repair, anti-aging, and antioxidant defense in *Apex1*-deficient mice, which may contribute to the observed deceleration of premature aging and establishment of redox equilibrium.

### Human *APEX1* expression as a function of age at death

Leveraging our previously published bulk RNA-sequencing work on postmortem human tissues (for SMART-Seq methodology and brain dissection images, please see ref.^37^), we reevaluated those data for expression of DNA repair genes as a function of age at death [all demographics of these cases, including ages at death and comorbidities, are provided in our prior reports^38,39^]. These tissues had been collected postmortem from the olfactory bulbs and amygdalae at UCLA and deidentified samples were donated to the NIH NeuroBioBank (see refs. ^37–39^). Additional information on RIN values and other variables is listed for each case ID on the NeuroBioBank web portal. The two-tailed *p* values obtained from linear regression analyses of age at death versus gene transcripts per million (TPM) for genes involved in DNA repair and antioxidant defense are illustrated (Fig. S8A), and linear correlations between *Apex1* and age at death are graphed and presented (Fig. S8B). A positive correlation between age and death and *Apex1* gene expression was observed in the amygdala, suggesting that the risk of mortality may be inversely associated with *Apex1* expression in some regions of the CNS. All original datasets for these human bulk RNA-seq studies can be found at NCBI’s Gene Expression Omnibus (ID number GSE297212).

### Human hippocampal transcriptomic alterations as a function of age

We also analyzed the human hippocampal transcriptome using bulk-RNA sequencing data and subject-level demographic information from the Genotype-Tissue Expression (GTEx) Project, version 10 (open-access release, https://www.gtexportal.org/home/downloads/). Among 255 individuals with hippocampal expression data, 218 subjects of the 50–59, 60–69, and 70–79 years of age groups were retained for downstream comparisons. Based on the DEGs across pairwise comparisons (60–69 group or 70–79 group vs. 50–59 group), functional enrichment analysis (Metascape) revealed remarkable age-dependent transcriptomic alterations. Figure S9A-B shows the top-10 upregulated GOs and top-10 downregulated GOs in each of the above described pairwise comparisons. The top upregulated GOs in the older age groups (60–69 or 70–79 group vs. 50–59 group) are enriched for cytokine production and leukocyte-mediated immune responses, whereas downregulated genes were enriched for oxidative phosphorylation, ATP synthesis, and synaptic signaling pathways (Fig. S9A-B). These results implicate chronic neuroinflammation and heightened oxidative stress, alongside impaired mitochondrial function and synaptic neurotransmission in the hippocampus of older age groups.

To examine age effects on pathways in the human hippocampus, we conducted targeted statistical modeling across a predefined panel of genes (Fig. S9 C-D). Violin plots overlaid with boxplots and jittered samples were generated to visualize expression distributions, and significant or nominal *post-hoc* differences were annotated directly on each panel. Importantly, many DEGs overlap between the human hippocampal results and our mouse microarray data. These include the pro-inflammatory genes *C1QC*, *C5AR1*, *CD14*, and *FCER1G*; oxidative stress regulatory genes *CYBA*, *GPX5*, and *SOD1*; and the pro-senescence gene *CDKN1A*, whose expression levels showed significant differences across different age groups (Fig. S9C). Among the DNA damage response and repair genes, several genes, including *RAD51C, RAD1, PTTG1, and POLB*, were expressed at lower levels in the 60–69 age group than the younger age group (Fig. S9D), supporting, but not proving, the notion that a decline in DNA repair activity helps drive brain aging. Notably, none of the DNA repair genes of interest showed reduced expression in the 70–79 age group compared with the younger age groups (Fig. S9D). A similar pattern was noted for the antioxidant defense genes *GPX5* and *SOD1*. The consistency of the U-shaped patterns in log-transformed DNA repair gene expression data suggests that humans who survived beyond their 60 s into their 70 s may not experience a decline in their ability to repair oxidative DNA lesions, even if they display higher pro-inflammatory markers. This potential endogenous protective mechanism may influence mortality, as has been suggested for the expression of DNA repair genes in studies of centenarians, who tend to also display lower mutation burdens than younger controls^40^.

### Apex1 cKO promotes astrogliosis in the hippocampus

We next studied the impact of CR and *Apex1* deletion on glial cell populations, focusing on microglia (Iba1^+^ cells) and astrocytes (GFAP^+^ cells). No differences in the numbers of Iba1^+^ cells were detected in CA1, CA3, or cortical regions across the four groups (Fig. S10A-D). In contrast, GFAP^+^ astrocyte numbers were markedly increased in CA1 (*p* < 0.0001) and CA3 (*p* < 0.0001), but not in cortical regions (*p* > 0.9999) of *Apex1* cKO AL mice compared to WT AL mice (Fig. S10E-H), suggesting astrogliosis in response to oxidative stress or neuronal injury in the hippocampus. This gliosis is likely driven by the accumulation of oxidative DNA damage and dendritic degeneration observed in *Apex1*-deficient hippocampal neurons. Importantly, CR abolished the increase in GFAP^+^ astrocytes in both CA1 (*p* < 0.0001) and CA3 (*p* < 0.0001) of *Apex1* cKO mice (Fig. S10 E, F, H), aligning with the preservation of neuronal integrity observed in these regions. These findings support the interpretation that CR not only protects neurons from hippocampal degeneration but also attenuates neuroinflammation by suppressing secondary astrocytic activation.

### Caloric restriction reduces body and peripheral organ weights

We investigated whether *Apex1* influences the metabolic effects of CR by monitoring bodyweight every 4 weeks and assessing organ weights at 48 weeks of age (Fig. S11). *Apex1* cKO mice maintained under AL conditions appeared phenotypically normal but showed lower body weights compared to WT AL mice (Fig. S11A). As expected, CR led to reduced bodyweight in both WT and *Apex1* cKO mice. Fasting blood glucose levels, measured prior to euthanasia at 48 weeks of age, followed a similar trend, with CR animals displaying lower glucose levels than their AL-fed counterparts (Fig. S11B). A robust positive correlation between blood glucose concentrations and bodyweight was also observed (Fig. S11C), further confirming the metabolic impact of CR. Neither *Apex1* knockout nor CR affected absolute brain weight (Fig. S11D). However, CR mice had smaller liver and spleen weights in both WT and *Apex1* cKO mice. Interestingly, kidney weight was reduced by CR only in *Apex1* cKO mice (Fig. S11D). When tissue weights were normalized to body weight, similar trends were observed for the liver, kidney, and spleen (Fig. S11E). Notably, brain weight as a proportion of body weight was higher in CR mice than in WT AL mice and *Apex1* cKO AL mice (Fig. S11E). These results suggest that CR induces robust systemic metabolic effects, including organ-specific changes, and that *Apex1* deletion may sensitize certain organs—such as the kidney—to CR-induced atrophy.

## Discussion

Aging is hypothesized to lead to cognitive decline because of compromised DNA repair mechanisms and the accumulation of oxidative DNA damage^1,21,41^. A number of diseases with rapid progression of aging phenotypes, such as Cockayne syndrome, Werner syndrome, and ataxia telangiectasia, are associated with impaired DNA repair systems^42^. In healthy cells, damaged DNA bases are rapidly removed and repaired by an array of DNA repair pathways, including mismatch repair, homologous recombination and nonhomologous DNA end joining (NHEJ), nucleotide excision repair (NER), and BER. Among the DNA repair mechanisms, BER is particularly important for postmitotic cells such as neurons. Although numerous studies report that loss of NER and transcription-coupled repair can lead to brain aging^43,44^, and recent work has begun to explore the role of *Apex1* in this context^17,45^, the specific contribution of *individual* BER enzymes to age-related neuronal dysfunction remains incompletely understood. The present study builds on this emerging body of work by using the new neuron-specific *Apex1* cKO mouse model to directly examine the relationship between DNA damage and aging, and to evaluate the neuroprotective effects of CR in this setting.

As early as 21 weeks of age, we observed a decline in the spatial memory of *Apex1* cKO mice relative to WT controls and increased oxidative DNA damage in their hippocampi at 48 weeks of age. Neural cells are particularly sensitive to ROS, and BER is the main pathway that repairs oxidative DNA damage after ROS attack^46–49^. Our results suggest that *Apex1* ensures proper function of BER during aging. Our group recently reported that *Apex1* cKO leads to premature aging, as revealed by earlier decline in spatial memory compared to age-matched, WT controls^17^, and the current report is, to the best of our knowledge, the first to suggest that a prolonged period of CR retards this decline. CR is the only intervention that effectively extends longevity in multiple species by counteracting age-related loss of oxidative DNA damage in tissues such as brain, liver, kidney, heart, and testis^50–52^. Accordingly, we observed that CR reduced oxidative DNA damage in the hippocampi of *Apex1* cKO CR mice. We examined a wide range of age-related phenotypes, from the structures of synapses and their electrophysiological properties to animal behavior. CR preserved the number of dendritic spines and spine lengths in CA1, as well as expression of synaptic proteins, and increased hippocampal field potentials. In the Morris water maze, animals without *Apex1* expression in the forebrain exhibited a premature decline in path efficiency and memory, starting at 21 weeks of age. CR improved spatial learning and memory and alleviated anxiety-like behaviors. Animals undergoing CR also showed an increase in open-field locomotor activities. Thus, we conclude that CR retards the accelerated aging process in *Apex1* cKO animals.

One consideration in our study design is that animals in the CR and AL groups experienced different housing conditions during feeding: CR animals were housed individually only during the daytime feeding period to ensure precise and consistent delivery of the restricted diet, whereas AL animals were pair-housed continuously to minimize social stress and cost. However, all animals—including CR mice—were returned to pair-housing during the dark cycle, during which most social activities are expected to occur in nocturnal animals. Although temporary individual housing could potentially influence behavioral outcomes such as anxiety or locomotor activity, CR itself has been shown to produce dominant and reproducible effects on neurobehavioral phenotypes, even under isolated housing conditions. Moreover, our study design was internally consistent, with genotype and dietary groups balanced within each housing condition. While we acknowledge that husbandry differences may introduce a minor limitation in interpretation of behavior data, we believe that the transient nature of the individual housing mitigates this concern. Nonetheless, future studies may consider fully harmonized housing conditions to further isolate dietary effects.

To assess the trajectory of cognitive aging, we employed a repeated testing paradigm in which behavioral tasks were administered at multiple time points over the study period. While repeated exposure can lead to task familiarity, a longitudinal design enabled us to track within-subject changes over time, increasing sensitivity to genotype- and age-related differences. This approach emphasizes memory retention and progression, rather than isolated memory acquisition. Although it may not distinguish de novo learning at each time point, the consistency of the beneficial effects of CR across the testing interval indicates that the effects are not masked by task familiarity alone, particularly in *Apex1* cKO mice.

Another potential limitation of our study is that the CR regimen was calculated based on baseline food intake without dynamic adjustment over time. While food intake in rodents increases from juvenile stages to adulthood, implementing continuous recalibration of caloric allotments over a 40-week period would require individualized feeding and housing, which can introduce additional stress and experimental variability. Consequently, we employed a group-housed CR paradigm with a fixed reduction, which differs from standard long-term CR protocols that incorporate longitudinal monitoring and adjustment of food intake. This design choice may underestimate the degree of caloric restriction at later ages. Nonetheless, CR animals maintained a sustained and significant reduction in body weight throughout the study, indicating a persistent caloric deficit. Future studies incorporating dynamic intake monitoring will be important to further refine CR paradigms and assess how protocol differences influence age-related phenotypes.

Notably, whereas reduced vGlut and GAD expression was observed in younger *Apex1* cKO mice in our prior study^17^, these differences were no longer detectable at 48 weeks of age in the current cohort. This finding suggests that alterations in these synaptic markers may evolve with age and warrants further investigation into their temporal dynamics in the context of DNA repair deficiency. In addition to neuronal structure and function, we also observed signs of glial reactivity in the aging *Apex1* cKO brain. Although the *Apex1* deletion was restricted to forebrain neurons, we detected a significant increase in GFAP^+^ astrocytes in the hippocampus at 48 weeks of age. This likely reflects a reactive astrocytic response to progressive neuronal injury. Astrocytes are highly responsive to disruptions in the local neuronal environment and undergo activation in response to DNA damage, synaptic dysfunction, and neurodegeneration. In *Apex1* cKO mice, the accumulation of oxidative DNA damage and dendritic degeneration likely triggered this reactive gliosis. Notably, CR reduced GFAP^+^ cell numbers in hippocampal regions CA1 and CA3, coinciding with preservation of neuronal integrity. These findings suggest that CR not only protects neurons but also mitigates neuroinflammation by suppressing secondary astrocytic activation. Beyond the central nervous system, we also observed systemic effects in *Apex1* cKO mice, including reductions in body weight, blood glucose levels, and kidney mass at 48 weeks of age. Although the deletion of *Apex1* was neuron specific, accumulating evidence suggests that chronic neuronal stress and neurodegeneration can influence peripheral physiology through altered neuroendocrine signaling, metabolic homeostasis, and inflammation^53,54^. For example, hypothalamic inflammation has been implicated in regulating systemic aging and energy balance^53^. It is therefore plausible that long-term neuronal dysfunction in *Apex1* cKO mice contributes to premature systemic aging phenotypes.

Although there was no difference in oxidative DNA damage between WT AL and WT CR mice at 48 weeks of age, this is not considered an “old” age in rodents, and CR therefore did not lower oxidative DNA damage in wildtype controls by this age. CR may protect against oxidative DNA damage by rejuvenating mitochondria and mitigating leakage of oxygen radicals out of the electron transport chain. It is also possible that CR may lead to less reliance on BER in *Apex1* cKO animals. In support of this idea, our previous study demonstrated that young adult *Apex1* cKO mice exhibit a significant increase in AP sites in the brain compared to wild-type controls^55^, underscoring the importance of *Apex1* activity in maintaining genomic stability. Although we did not assess AP site accumulation in aged animals in the present study, future investigations should determine if CR mitigates this accumulation in aging brains lacking *Apex1*. This would help clarify the extent to which CR preserves neuronal function through modulation of DNA repair demand or efficiency. In addition to *Apex1*, the downstream gene DNA polymerase beta (*PolB*) also plays an active role in repair of genomic and mitochondria DNA^7,56^. Although we observed decreased oxidative DNA damage after CR, we did not gather evidence that *Apex1* knockout or CR impacts the expression of *PolB*. The interaction between CR and other proteins along the BER pathway is beyond the scope of the present study and warrant further long-term studies.

The preservation of neuronal structure and function by CR in *Apex1* cKO mice is consistent with emerging evidence on the salutary properties of CR in animal models of various neurodegenerative disorders^57,58^. The fact that CR yields such pronounced neuroprotection suggests that neurons, even in later life, maintain inducible programs that can counteract DNA damage and avert cell loss. These benefits likely reflect a combination of local cellular adaptations and CR-driven changes in whole-body physiology, including modulation of inflammatory tone and endocrine pathways. Longitudinal work on CR in non-human primates highlight the complexity of biological responses to CR^59,60^, including the impact of diet composition, feeding regimens, and age of onset of the dietary intervention. Nonetheless, even in human subjects, 25% caloric restriction for two years decelerated aging in randomized, non-obese adults enrolled in the Comprehensive Assessment of Long-Term Effects of Reducing Intake of Energy (CALERIE) trial, according to the sensitive DunedinPACE blood DNA methylation algorithm, which is designed to predict overall physiological decline until midlife^61^.

The present study focused exclusively on male mice to ensure sufficient statistical power, draw on the extensive aging literature in males, and minimize variability from hormonal fluctuations in females. While this approach was appropriate for our initial investigation, the lack of female subjects remains a limitation. Future studies will be needed to assess whether the effects of CR and *Apex1* deficiency are consistent across sexes or show sex-specific differences. In this context, it is worth noting that no key sex differences were identified for the aforementioned blood DNA methylation analysis of the CALERIE trial subjects^61^.

One major strength of the *Apex1* cKO model is the atrophy of postsynaptic structures in the absence of frank loss of neuronal (NeuN^+^) somata in the hippocampal formation. Outside of early neurodevelopment, a large body of recent evidence supports a lack of significant degeneration of forebrain neurons with advancing age^62^. Rather, normal aging induces regression of neuronal processes and functional alterations in their electrophysiological features (reviewed by Dickstein et al. in ref. ^63^). Accordingly, it is these particular features of normal aging, alongside the expected cognitive decline, that are accelerated with *Apex1* cKO—rather than neuronal death per se. The age-related mechanisms underlying these structural/functional changes are not well understood, but our study now points to potential oxidative DNA damage from *Apex1* dysfunction as a mediator of dendritic shrinkage, LTP breakdown, and memory deterioration. In conclusion, the present study addresses the structural and functional consequences of CR and *Apex1* on the mouse hippocampus during premature aging. Use of the new *Apex1* cKO line will reduce the time and cost of typical aging studies in rodents and may be a useful tool for testing anti-aging interventions, defining the long-term health effects of nutrition, and discovering more viable alternatives to CR.

## Methods

### Mouse model

*Apex1* ^flox/flox^ mice on C57BL/6 background were generated as described^55^, where LoxP sites flank exons IV and V of the *Apex1* gene. *Apex1* cKO mice were obtained by crossing B6.CaMKIIα-Cre mice^64^ (Strain # 005359, the Jackson Laboratory) and *Apex1* ^flox/flox^ mice for two generations (genotype: *Camk2a*-*Cre*^+/-^;*Apex1*^flox/flox^). Hemizygous CaMKIIα-Cre^+/-^ mice served as age- and sex-matched WT control mice for the *Apex1* cKO mice. Only male (8- to 48-week-old) mice were used in this study. Mice were housed in a temperature- and humidity-controlled animal facility with a 12 h light/dark cycle. Water was available for all groups of animals and ad libitum food was available for AL control animals, while food containers were left empty for CR animals (see below). Mice were weighed once every four weeks and were visually inspected weekly for gross morphological abnormalities. All animal procedures were approved by the University of Pittsburgh Institutional Animal Care and Use Committee and performed in accordance with the *National Institutes of Health Guide for the Care and Use of Laboratory Animal*. All efforts were made to minimize animal suffering and the number of animals used. We have complied with all relevant ethical regulations for animal use.

### Diets

All animals were fed AIN93G synthetic pellets (Research Diet Services B.V.; gross energy content 4.9 kcal/g dry mass, digestible energy 3.97 kcal/g). Average daily dietary intake was measured prior to the study; each mouse ate ~3.3 g food per day under *ad libitum* conditions. All animals of both genotypes were randomly assigned to AL or CR groups by lottery. CR was initiated at 8 weeks of age with 10% food reduction (3.0 g/day), and was increased weekly by 10%, until 30% CR was achieved (2.3 g/day) and maintained from 10 weeks of age onward. The control diet animals received free access to food until 48 weeks of age. Animals in the CR groups were individually housed in separate cages from 9 AM to 5 PM while exposed to specified amounts of food, to ensure accurate food delivery. Half of the calculated food was given to each animal at 9 AM, while the other half was given at 5 PM. The initial CR allotments were calculated based on baseline food intake from age-matched AL controls and were held constant thereafter, in accordance with standard protocols used in aging research. While we recognize that food intake can change with age, dynamic recalibration of food allotment over a 40-week period would require continuous individualized feeding and introduce additional stress and variability. Thus, our goal was to model a consistent and widely accepted CR regimen, rather than to match precise intake across age.

### Blood glucose measurement

Mice were euthanized by CO₂ asphyxiation, and blood was immediately collected from the heart. Blood glucose levels were measured using a Freestyle Mini blood glucose meter. Measurements were taken after feeding, at the beginning of the dark period.

### Immunohistochemistry and image analysis

At 48 weeks of age, mice were deeply anesthetized and euthanized in a CO_2_ chamber prior to transcardial perfusion with 0.9% NaCl. Brains were harvested and fixed in 4% (wt/vol) paraformaldehyde in PBS for one week followed by cryoprotection in 30% (wt/vol) sucrose in PBS. Frozen serial coronal brain sections (25 µm thick) were prepared on a cryostat (Microm HM459, Thermo Scientific). Sections were blocked with 5% donkey serum in PBST for 1 h, followed by overnight incubation (4 °C) with the following primary antibodies: rabbit anti-NeuN (1:500; EMD Millipore, Billerica, MA, USA), mouse anti-APE1 (1:500; Abcam, Cambridge, United Kingdom), rat anti-vGlut1 (1:100; NeuroMab, Davis, CA, USA), rabbit anti-GAD (1:1000; EMD Millipore, Billerica, MA, USA), goat anti-Iba1 (1:500; Abcam), goat anti-GFAP (1:500; Abcam), mouse anti-8-OHdG (1:500; Abcam), and rabbit anti-PolB (1:200; Acris, Bronx, NY, USA). After washing, sections were incubated for 1 h at room temperature with donkey secondary antibodies conjugated with DyLight 488 or Cy3 (1:1000, Jackson ImmunoResearch Laboratories, Inc., West Grove, PA, USA). Alternate sections from each experimental condition were incubated in all solutions except the primary antibodies to assess nonspecific staining. Fluorescence images were captured with confocal microscopy (see below).

Excitatory or inhibitory protein expression was measured through v-Glut1 and GAD immunostaining, respectively. Stained sections were analyzed with the software Imaris (Version 9.0, Bitplane, Belfast, United Kingdom). Z-stack images (1 µm apart) were captured at 60× magnification on an Olympus Fluoview FV1000 confocal microscope using FV10-ASW 2.0 software (Olympus America, Center Valley, PA, USA). 3D-images were reconstructed using Imaris and pre- and post-synaptic proteins were analyzed using the “spot-detection” feature in Imaris.

### Behavioral tests

#### Morris water maze test

To evaluate long-term cognitive function, the Morris water maze test was initiated at 8 weeks of age and repeated every 4-5 weeks using ANY-MAZE software, as previously described^65^. Briefly, a square platform (11 cm × 11 cm) was submerged in a pool of opaque water (109 cm in diameter). To assess learning, mice were placed into the pool from one of four locations and allowed 60 s to locate the hidden platform. At the end of each trial, the mouse was placed on the platform or allowed to stay on the platform for 30 s, with prominent spatial cues displayed around the room. The time required for the animal to find the platform (escape latency) along with path efficiency, was recorded for each trial. Path efficiency was calculated by the shortest possible distance from the start location to the platform divide by the total distance traveled by the animal. Three trials were performed on each day for 4 consecutive days. After the learning test, the platform was removed and a single 60 s probe trial was performed. The percentage time spent in the target quadrant, platform site crossings, and the latency of first entry into platform area were recorded to assess spatial reference memory. Swim speed was also recorded to assess differences in gross locomotor abilities, which can confound the interpretation of water maze tests. The mice that cannot remain upright in the water or fail to find the visible platform were excluded from the experiment (Supplementary Table 1).

#### Visible platform test

To avoid the confounding effects of potential age-related loss of vision in the Morris water maze test, we performed the visible platform test on the final testing day, at 48 weeks of age. Animals that failed to find the platform were then excluded from further analyses. As no training was performed before the visible platform test, a high range of variation was observed within each group. No differences across groups were observed, suggesting a lack of vision-related confounding factors.

#### Open-field test

In the open-field test, each mouse was placed near the wall of a 30 × 25 × 20 cm^3^ open-field arena, and the movement of the mouse was recorded by USB webcam (LifeCam HD-6000, Microsoft) for 10 min and analyzed by ANY-MAZE software. The total distance traveled, average speed in the field, time in the corner, and time in center (15 × 15 cm^2^ imaginary squares) were measured. The arena was cleaned with 70% ethanol and wiped with paper towels between each trial. Animals that remain immobile for up to 50% or greater of the total trial duration were excluded from the analysis.

#### Novel object recognition test

Object recognition experiments were conducted in the same open-field arena (30 × 25 × 20 cm^3^) as above. The open-field arena and the stimulus objects were cleaned with 70% ethanol between trials. A video camera was positioned over the arena, and the behavior of the mice was recorded using a video tracking and analysis system by ANY-maze software. Two identical objects (denoted as A and A’) were placed in the arena, and mice were allowed to explore for 10 min. Another 10 min test session was performed 1 h later, and one of the twin objects was replaced with a novel object (denoted by B)^66^. Two parameters (number of head entries into the object zone and head time spent in the object zone) were quantified to assess mouse exploratory behavior toward familiar and novel objects. The Preference Index was calculated for each parameter, as the percentage time exploring the novel object versus both objects, and the percentage head entries into the zone of the novel object versus both objects, respectively^66,67^. All behavior experiments were conducted 2 h after food intake in both CR and AL animals by an observer blinded to group assignments. Animals were excluded from the analysis if they failed to reach a minimum of 10 seconds of active exploration per object during either the familiarization or the test phase.

### Electrophysiology

Hippocampal slices (*n* = 7–12 slices/group from 7-10 animals/group) were obtained at 48 weeks of age. Hippocampal slices from both hemispheres were sectioned in 400 µm increments and submerged in oxygenated artificial cerebrospinal fluid (aCSF) at 34 °C for 1 h and room temperature for the next hour. Field potentials were amplified 100×, Bessel-filtered at 1 kHz, and digitized at 10 kHz using a Multiclamp 700B amplifier and Digidata 1550a digitizer (Molecular Devices). Field excitatory postsynaptic potential potentiation (fEPSP) was evoked with a bipolar tungsten-stimulating electrode by orthodromic stimulation of CA3 pyramidal cell axons in the Schaffer collateral pathway (FHC, ME, LOT# 213025). Glass micropipettes filled with aCSF (resistance 1–3 MΩ) were used to measure fEPSPs in the recipient CA1 stratum radiatum, ~1 mm from the point of stimulation. After placement of stimulating and recording electrodes in the stratum radiatum, an initial input-output curve was recorded by stimulating at intensities ranging from 12.5 μA up to the intensity (300 μA) that yielded fEPSPs of maximal slope, with a 0.2 ms single pulse every 30 s. For each brain slice, the test stimulus intensity was calibrated to elicit an fEPSP amplitude equal to 40–60% of the maximal response. After stable baseline recording for at least 20 min, LTP was induced using tetanic stimulation (three trains of 100 pulses at 100 Hz, delivered at 0.5 Hz) with the same stimulus intensity, and then fEPSP was recorded up to 60 min. Inclusion criteria: LTP induction was considered valid if the mean fEPSP slope between 40 and 60 min post-tetanus was ≥130% of baseline. If the average fEPSP slope during this period fell below baseline, LTP induction was considered a failure. Slices failing either criterion were excluded a priori from analyses. All LTP data reported were filtered according to this standard.

### Golgi staining and analysis

Animals were deeply anesthetized and euthanized in a CO_2_ chamber prior to cardiac perfusion with 0.9% NaCl solution. One hemisphere of each mouse was quickly harvested and processed for Golgi impregnation using FD Rapid GolgiStain Kit (FD Neurotechnologies, Inc., Columbia, MD). Brain slices were cut coronally at a thickness of 120 µm on a cryostat (Microm HM459, Thermo Scientific). Sections were mounted on gelatin-coated glass slides (FD Neurotechnologies), dried and stored overnight in a dark chamber away from light. Brain slides were subsequently stained, dehydrated and prepared for imaging according to the manufacturer’s instructions (FD Neurotechnologies).

Images of Golgi-impregnated single neurons were captured with 20× objectives under brightfield illumination using an Olympus BX51 microscope. Only neurons with intact basal as well as apical dendrites were analyzed. Labelled neurons were selected from the center of the 120 µm slice to avoid the confounding impact of the cutting^68,69^. Five mice were randomly selected from each group and 20 CA1 pyramidal neurons per mouse (100 neurons in total per group) were randomly selected for morphological analysis. Neurons had to reside in the middle 30 µm of the Z-plane with no or minimal breaks in order to be included in the calculation. Golgi-labelled neurons were then traced based on Imaris algorithms in a semi-automated manner. Parameters included neurite length, branch points, and concentric Sholl intersections at different radii from the soma within the Sholl sphere. The tracing starting point was set at the center of the soma and traces were made for both apical and basal dendrites. For dendritic spine density and morphology, images were captured with a 100× oil objective (Olympus).

### Microarrays

Microarray analyses were performed in the Genomics Research Core at the University of Pittsburgh. Mouse hippocampi from one hemisphere (left or right) of four groups (WT AL, WT CR, *Apex1* cKO AL, and *Apex1* cKO CR) were dissected and snap-frozen on dry ice. Total tissue mRNA was extracted using Trizol (Qiagen), according to the manufacturer’s instructions. Transcription of cDNA was performed using WT Plus reagent kit (Thermo Fisher) according to the manufacturer’s instructions. Reverse transcription was performed using 100 ng total RNA and dNTP-T7 random primers. Second-strand synthesis and in vitro transcription created amplified cRNA. cDNA from a second reverse transcription reaction was fragmented and end-labeled with biotin for hybridization to the Affymetrix Clariom S mouse array. Following overnight (16 h) hybridization at 45 ˚C with rotational mixing at 60 rpm, arrays were processed on a GeneChip 450 Fluidics Station using manufacturer-specified protocols. To remove unbound samples, arrays were first washed with non-stringent wash buffer A. The GeneChips were then stained for 10 min in stain cocktail 1. Buffer A was again used to wash off excess stain. Signal amplification was achieved by 10 min incubation with stain cocktail 2 followed by a second 10 min incubation with stain cocktail 1. The chip was washed with high-stringency buffer B and filled with Array Holding Buffer before being removed from the fluidics station and scanned using the GeneArray 3000 scanner. First-level image analysis was performed using Affymetrix Expression Console. Four biological replicates were used for each experimental condition. Detected probes were analyzed using Transcriptome Analysis Console (Affymetrix). Quality control analyses revealed that one sample from the *Apex1* cKO AL group deviated substantially from its group; this sample was therefore excluded prior to differential expression analysis. The final dataset included 15 animals: n = 3 for Apex1 cKO AL and *n* = 4 for each of the other three groups.

### Reverse transcription polymerase chain reaction (RT-PCR)

The total RNA of brain tissue (hippocampi) was first extracted using TRIzol (Invitrogen). RNA purity and concentration were assessed via A260/280 ratios using NanoDrop microvolume spectrophotometers. Then the first-strand cDNA was synthesized for qPCR templates. Bio Rad iTaq™ Universal SYBR® Green Supermix was used for qPCR to determine the relative expression levels of the target genes. Briefly, qPCR reactions were run in triplicate on a 96-well plate with 1 μL cDNA as template in a final reaction volume of 10 μL per well for each sample. Plates were loaded onto the ABI-700 sequence Detection System PCR machine (Applied Biosystems, USA). Amplification of target genes was initially started at 95 °C for 5 min, followed by cycling conditions: 40 cycles of 95 °C for 10 s (denaturation), 56 °C for 30 s (annealing), and 72 °C for 30 s (extension). Melting curve analysis was performed from 60 °C for 30 s at a linear ramp rate of 0.5 °C /s with data acquisition every 0.5 °C. Gene expression was normalized to *Gapdh* using the ΔCt method. Relative fold changes were calculated using 2^−ΔΔCt^ method, with the average ΔCt of the control group serving as the calibrator. A detailed list of primer sequences for the target genes used in this study is provided in Supplementary Table 2.

### Human bulk RNA sequencing

Postmortem human olfactory bulb and amygdala tissues were collected with IRB approval at UCLA for the NIH NeuroBioBank, as described in refs. ^37–39^. All tissue samples had been deidentified. The postmortem intervals and ages at death did not differ across groups (see our prior work^38^). The UPMC Health Sciences Sequencing Core at UPMC Children’s Hospital of Pittsburgh completed the RNA isolation, library preparation, sequencing, and data analyses (described in ref.^37^). The images of the amygdalar dissections have also been published (see Fig. S11 in ref. ^37^). All demographics, including ages at death and comorbidities, such as Lewy body disease, are provided in our prior reports^38,39^. Additional information linked to each case ID can also be found on the NeuroBioBank web portal.

### Human hippocampal transcriptomic and enrichment analyses

We characterized age-associated transcriptional alterations in the human hippocampus using bulk RNA-sequencing data and demographic information from the Genotype-Tissue Expression (GTEx) Project, version 10 (open-access release, https://www.gtexportal.org/home/downloads/). Of 255 individuals with available hippocampal expression profiles, 218 subjects aged 50–59, 60–69, or 70–79 years were retained for downstream analyses. Gene-level TPM values were extracted and log-transformed using log_2_(TPM + 0.1) to stabilize variance. Differential expression was evaluated across pairwise age contrasts (60–69 vs. 50–59; 70–79 vs. 50–59) to derive direction-specific gene sets. Gene Ontology (GO) over-representation analysis was performed for each direction group using clusterProfiler (v4.18.2) in R, with a primary focus on the Biological Process ontology. Entrez gene identifiers were mapped using the org.Hs.eg.db annotation database (v3.22.0). Statistical significance was assessed using Benjamini–Hochberg false-discovery rate correction, and enriched GO terms with adjusted *p* ≤ 0.05 were retained.

To examine age effects on human hippocampus, we conducted targeted statistical modeling across a predefined panel of genes. For each gene, one-way ANOVA tested differences in log-transformed expression across the three age groups, yielding F statistics, *p*-values, and generalized effect sizes. Pairwise contrasts were evaluated using Tukey’s HSD with adjusted *p*-values. Violin plots overlaid with boxplots and jittered samples were generated to visualize expression distributions, and significant or nominal *post hoc* differences were annotated directly on each panel.

### Statistics and reproducibility

All measurements were performed with investigators blinded to genotype and diet. The sample size of each experiment was determined based on our pilot study. The type I error rate was limited using an α level of 5%, with Bonferroni’s adjustment for pairwise comparisons. Data in scatterplot bar graphs and line graphs are presented as mean ± SD. Data in box-and-whisker plots are presented as interquartile ranges. Comparisons of means between two groups were performed using a two-tailed *t*-test for normally distributed data and a Mann-Whitney test for non-normally distributed data. Differences among multiple groups were analyzed using ordinary one-way ANOVA or repeated measures two-way ANOVA, followed by Bonferroni *post hoc* tests for normally distributed data, whereas non-normally distributed data were analyzed using Kruskal-Wallis one-way ANOVA followed by Dunn’s *post hoc* test. Two-tailed Pearson or Spearman correlation analyses were used for normally distributed or non-Gaussian data, respectively. A *p*-value less than or equal to 0.05 was deemed statistically significant. All testing was two-sided. For additional statistical methods, please refer to the section above on human hippocampal transcriptomic analyses. Comprehensive statistical analysis results are provided in Supplementary Data 1, 2, and 3. The source data behind the graphs are provided in Supplementary Data 4.

### Reporting summary

Further information on research design is available in the Nature Portfolio Reporting Summary linked to this article.

## Supplementary information

Transparent Peer Review file

Supplementary Information PDF

Description of Additional Supplementary Files

Supplementary Data 1

Supplementary Data 2

Supplementary Data 3

Supplementary Data 4

Reporting Summary

## Data Availability

Microarray data are deposited in the Gene Expression Omnibus database at the National Center for Biotechnology Information (GSE324956 and GSE297212). All other relevant data supporting the findings are available within the paper and the Supplementary Materials. Source data used for generating the plots are available in the Supplementary Data file associated with the manuscript. Additional information and reagents are available from the corresponding author upon reasonable request.
