# Supplementary material for: Caloric restriction decelerates premature aging and cognitive decline in mice with deficient DNA repair
Source: Commun Biol. 2026 May 8;9:960. doi: 10.1038/s42003-026-10182-3 (PMC13369994; doi:10.1038/s42003-026-10182-3)
Supplement: Supplementary file 2 — Supplementary Information PDF [file 42003_2026_10182_MOESM2_ESM.pdf]

Supplementary Figures

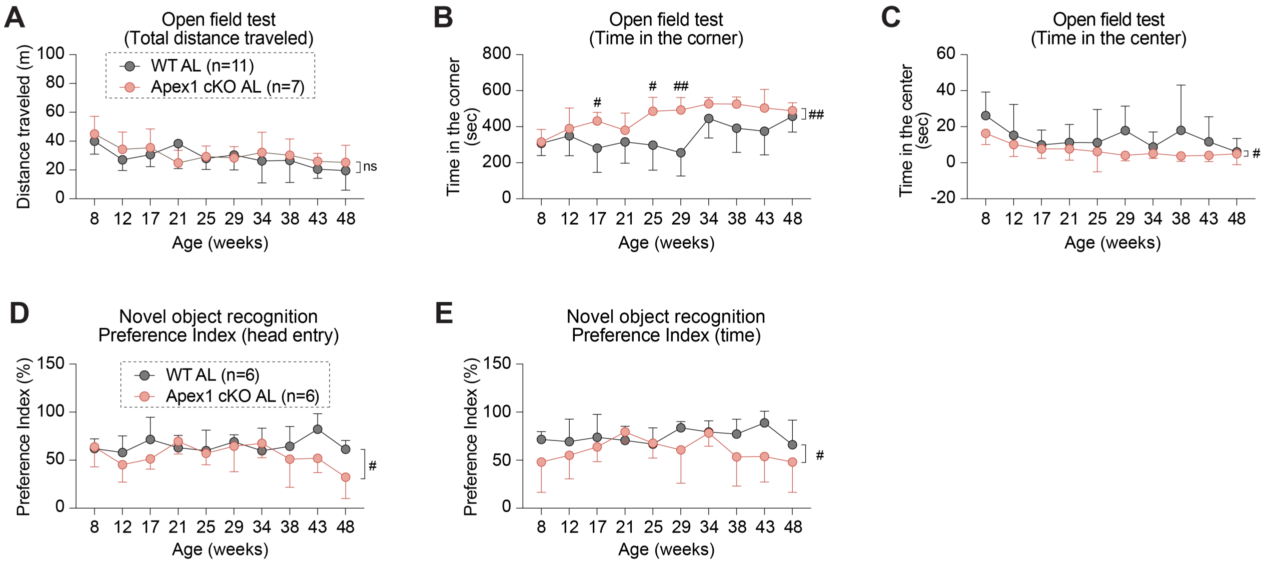

Supplementary Figure 1. Cognitive deficits in *Apex1* cKO mice, detected by the open field test and novel object recognition test. (A-C) The distance traveled (A), time spent in the corner (B), and time spent in the center (C) of the open field test were compared between WT and *Apex1* cKO mice over 40 weeks under *ad libitum* (AL) feeding conditions. WT n = 11, *Apex1* cKO n =7. **(D-E)** Preference index (head entry; D) and preference index (time spent on exploring novel object versus exploring both objects; E) in the novel object recognition test of WT and *Apex1* cKO mice over 40 weeks. n = 6 per group. ns = no significant difference. # $p < 0.05$, ## $p < 0.01$ vs. WT AL mice by repeated measures two-way ANOVA/*Bonferroni* tests.

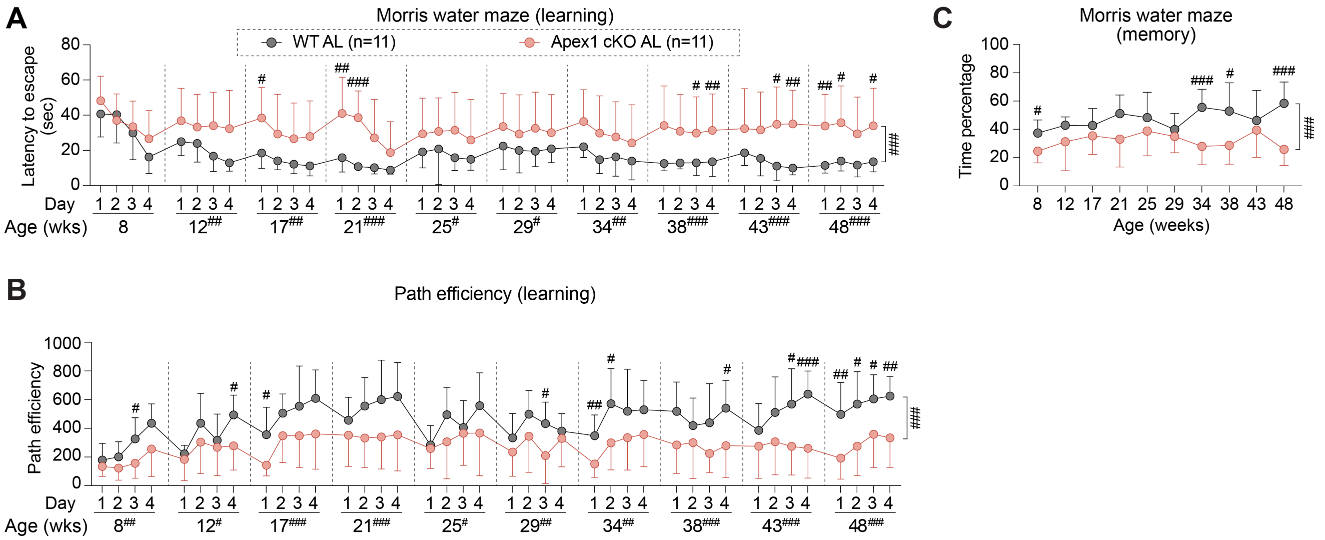

Supplementary Figure 2. Progressive spatial cognitive deficits in *Apex1* cKO mice, detected by the Morris water maze test. (A) Latency to escape in the learning phase of the Morris water maze over 40 weeks under *ad libitum* (AL) conditions. **(B)** Path efficiencies in the water maze test. **(C)** Percentage of time spent crossing the target platform during the probe trial test over 40 weeks. n = 11 per group. # $p < 0.05$, ## $p < 0.01$, ### $p < 0.001$ vs. WT AL mice by repeated measures two-way ANOVA/*Bonferroni* tests.

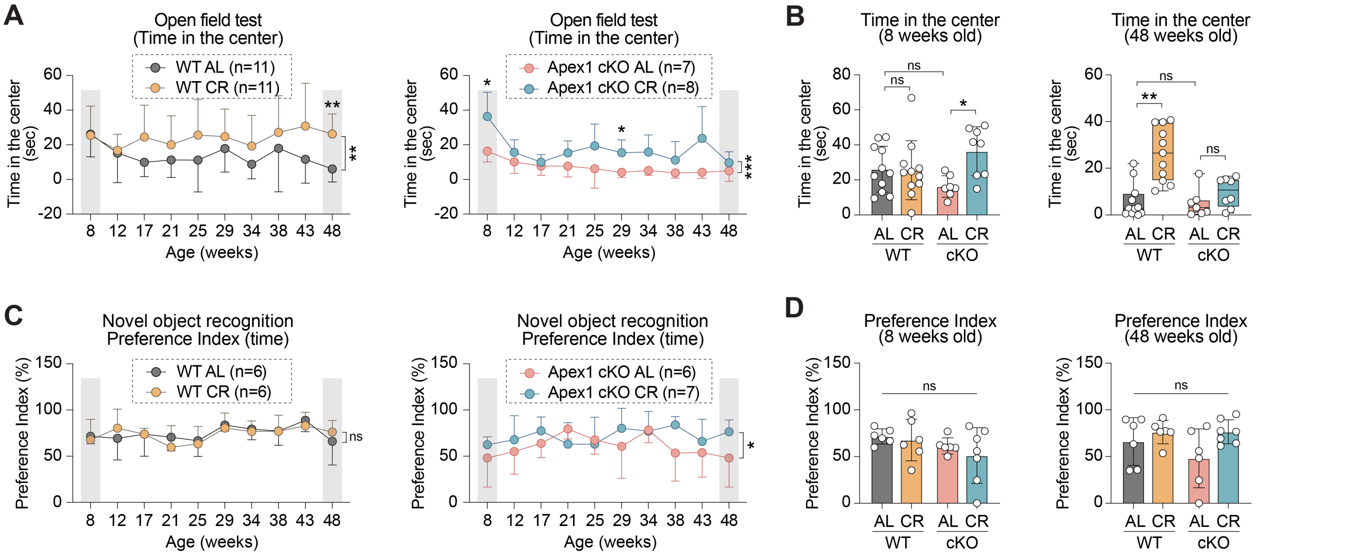

Supplementary Figure 3. Caloric restriction has modest effects on certain parameters of the open field test and novel object recognition test. (A) Time spent exploring the center zone in the open field over 40 weeks. Gray bars indicate the time points for multiple comparisons between WT and *Apex1* cKO groups in B. **(B)** Time spent exploring the center zone in the open field test at week 8 (left panel) and 48 (right panel), respectively. n = 11 per WT group. n = 7-8 per *Apex1* cKO group. **(C)** Novel object preference (% time spent exploring novel object versus both objects) over 40 weeks. Gray bars indicate the time points for multiple comparisons between WT and *Apex1* cKO groups in D. **(D)** Preference index % (time spent exploring novel object versus exploring both objects) in the novel object recognition test at week 8 (left panel) and 48 (right panel), respectively. n = 6 per WT group, n = 6-7 per *Apex1* cKO group. ns = no significant difference. *p < 0.05, **p < 0.01, ***p < 0.001 vs. AL mice by repeated measures (line graphs) or ordinary (bar graphs) two-way ANOVA/*Bonferroni* tests for normally distributed data and Kruskal-Wallis one-way ANOVA/*Dunn* for non-normal data distributions.

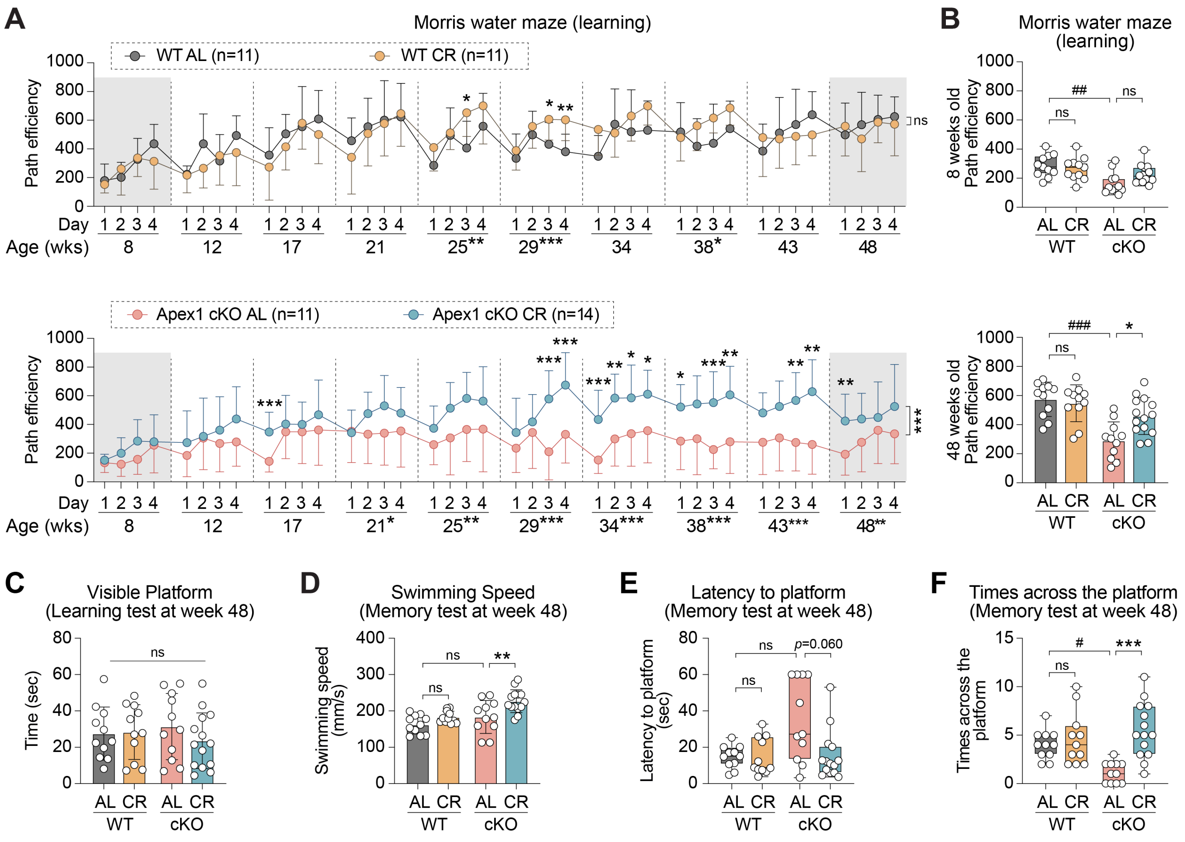

Supplementary Figure 4. Caloric restriction improves spatial cognition in *Apex1* cKO mice at week 48, detected by the Morris water maze test. (A) Path efficiencies in the water maze test of WT (top) and *Apex1* cKO (bottom) mice over 40 weeks. Gray bars indicated the time points (week 8 and 48) for multiple comparisons between WT and *Apex1* cKO groups, presented in B. **(B)** Path efficiencies at week 8 (top) and 48 (bottom) in the learning test of Morris water maze. **(C)** Latency to reach the visible platform of the Morris water maze during the learning test at week 48, to confirm comparable visual acuity across all groups. **(D)** Swimming speeds in the water maze at week 48. **(E-F)** Latency to reach the platform area and times across the platform area (within 60 seconds of test time) during the memory test in the Morris water maze, respectively. ns = no significant difference. # $p < 0.05$, ## $p < 0.01$, ### $p < 0.001$ *Apex1* cKO AL vs. WT AL mice; *$p < 0.05$, **$p < 0.01$, ***$p < 0.001$ vs. AL mice of the same genotype by repeated-measures (A) or ordinary (B bottom panel, C, D) two-way ANOVA/*Bonferroni* tests for data with normal distributions and Kruskal-Wallis one-way ANOVA/*Dunn* tests for non-normally distributed data (B top panel, E, F).

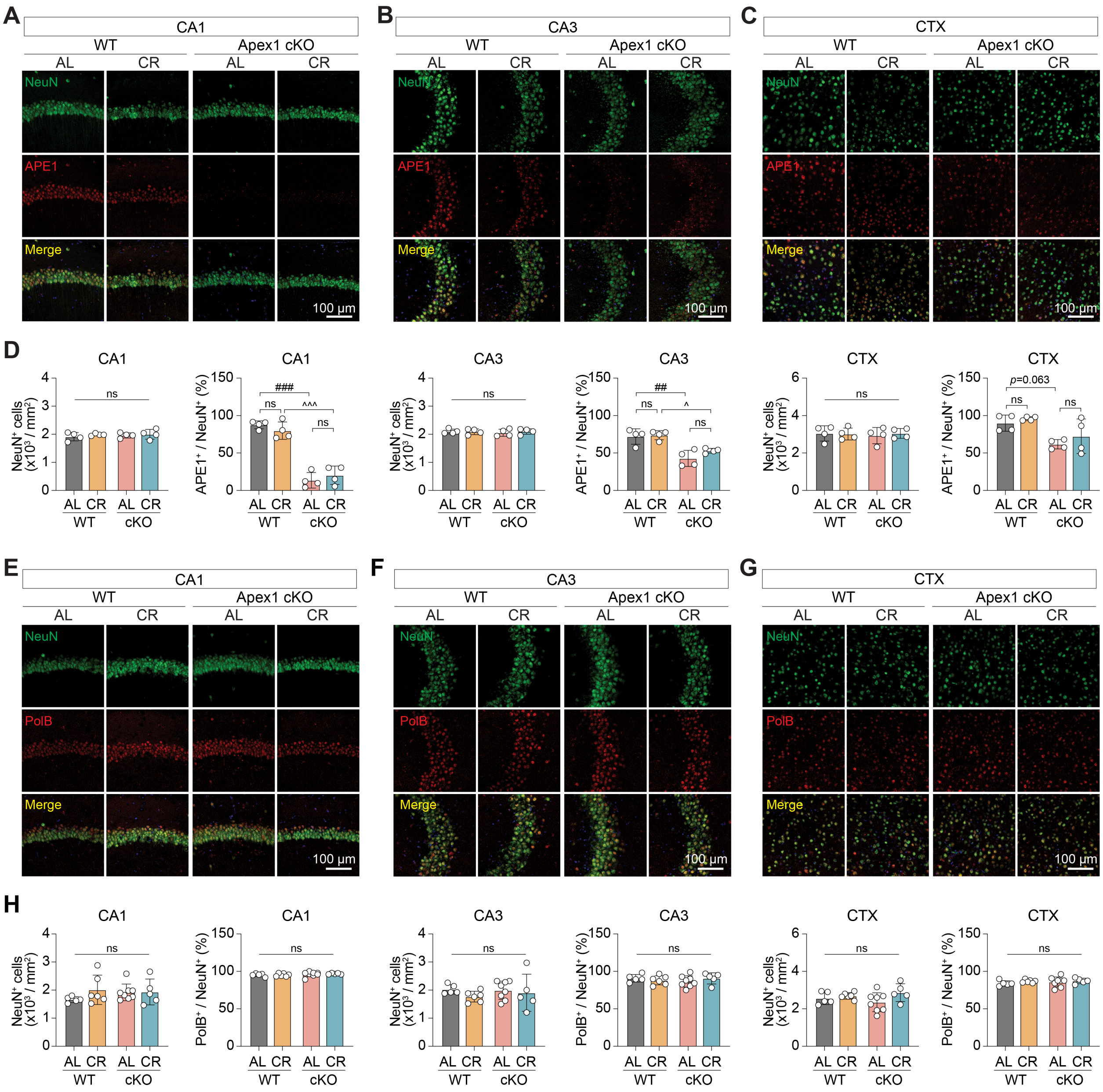

Supplementary Figure 5. Expression of APE1 and PolB in *Apex1* cKO mice. (A-D) Representative immunofluorescence images (A-C) and quantifications of NeuN$^+$ cell densities and APE1$^+$/NeuN$^+$ cell densities in hippocampal CA1, CA3, and cortex (CTX) at week 48 (D). **(E-H)** Representative immunofluorescence images (E-G) and quantifications of NeuN$^+$ cell densities and PolB$^+$/NeuN$^+$ cell densities in hippocampal CA1, CA3, and CTX at week 48 (H). Scale bars: 100 μm. Data are presented as mean ± SD. ns = no significant difference. ## $p < 0.01$, ### $p < 0.001$, ^ $p < 0.01$, ^^^ $p < 0.001$ vs. indicated group by two-way ANOVA/*Bonferroni* tests.

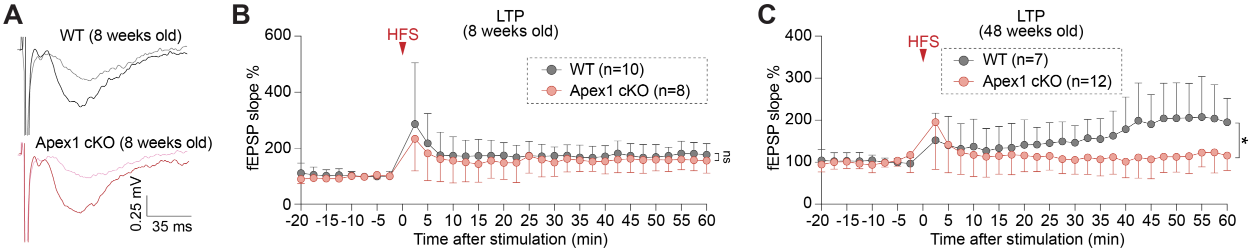

Supplementary Figure 6. LTP reduction in *Apex1* cKO mice at 48 weeks. (A) Representative traces of evoked LTPs in WT AL and *Apex1* cKO AL mice at week 8. **(B-C)** Quantification of % fEPSP slopes at week 8 (B) and 48 (C). Data are presented as mean ± SD. ns = no significant difference. *$p < 0.05$ by repeated-measures two-way ANOVA/*Bonferroni* tests.

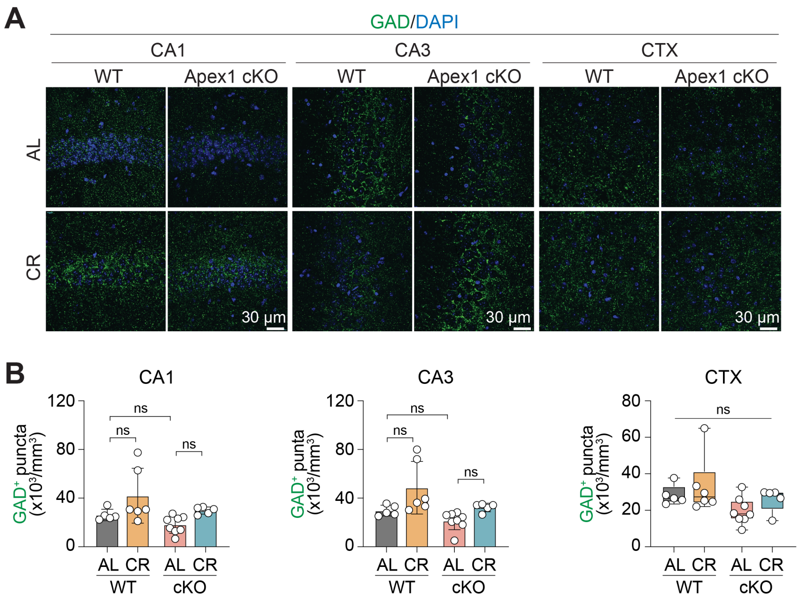

Supplementary Figure 7. Lack of changes in GAD expression after caloric restriction. (A) Representative immunofluorescence images of DAPI (blue) and GAD (green) in hippocampal CA1, CA3, and cortex (CTX) at week 48 (scale bar: 30 µm). **(B)** GAD^{+} puncta densities were quantified from confocal Z-stack images. Data are presented as mean ± SD or as boxplots with interquartile ranges. ns = no significant difference. Comparisons were made by two-way ANOVA/*Bonferroni* tests for normally distributed data and Kruskal-Wallis one-way ANOVA/*Dunn* tests for non-normally distributed data.

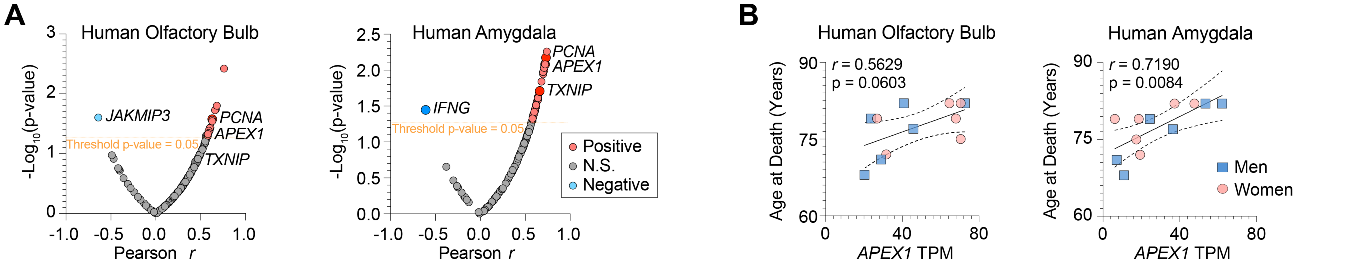

Supplementary Figure 8. Human olfactory bulb and amygdala gene expression. (A) Volcano plots of p values and correlation coefficients from two-tailed Pearson linear regression analyses of age at death versus transcripts per million (TPM) for DNA repair and antioxidant defense genes in human olfactory bulb and amygdala tissues. **(B)** Spearman's and Pearson's two-tailed correlation analysis of *APEX1* expression in human olfactory bulb and amygdala versus age at death, respectively.

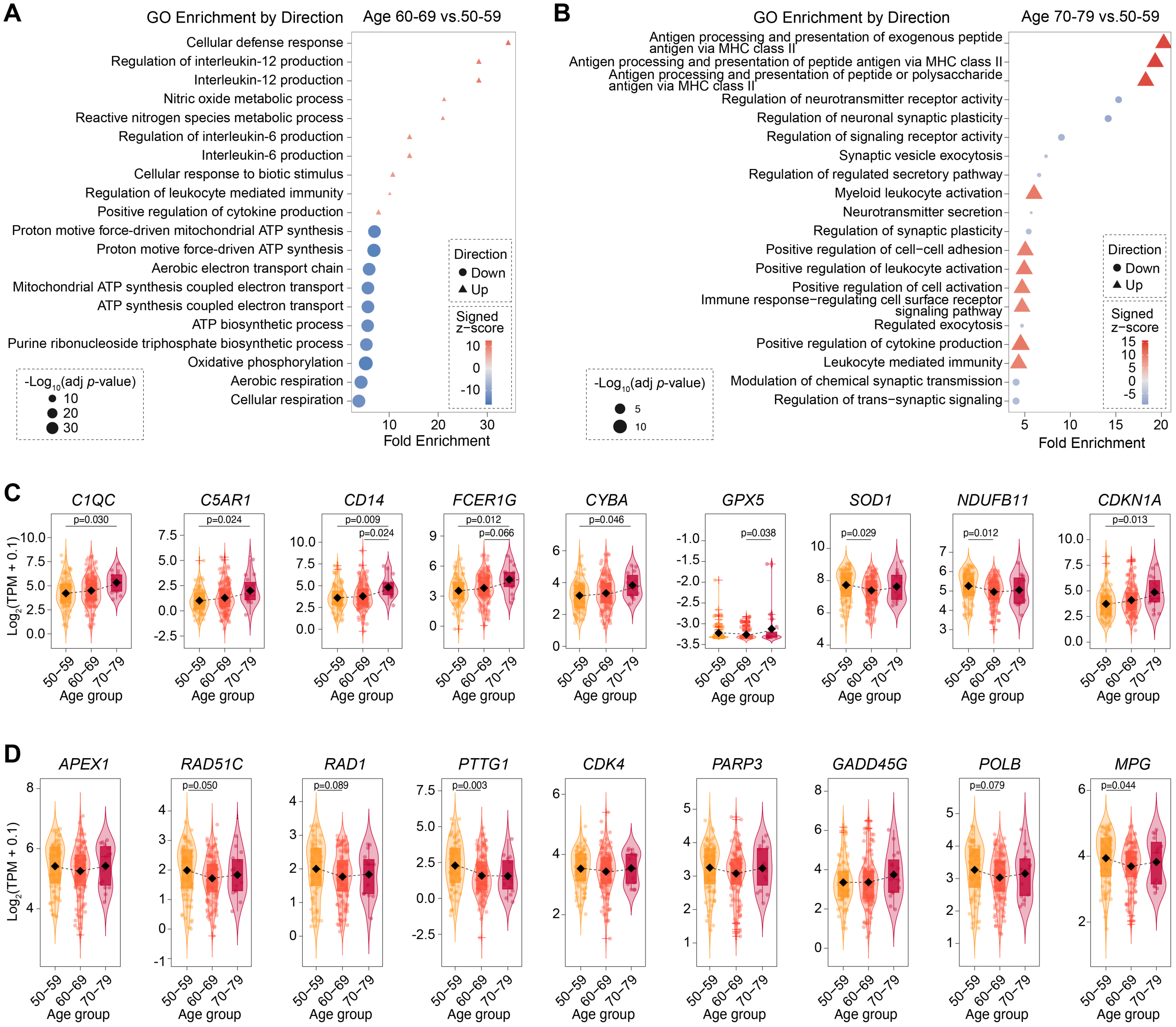

Supplementary Figure 9. Human hippocampus gene expression. (A-B) Directional enrichment of Gene Ontology (GO) pathways across age-associated gene expression contrasts. GO biological processes enriched among genes exhibiting higher or lower expression levels in older (60-69 or 70-79) versus younger (50-59) age groups. Each point represents one GO term. The x-axis displays fold enrichment, the point size reflects enrichment significance as -$\log_{10}$ (adjusted p-value), and the point color denotes the signed enrichment z-score. Directional differences are indicated by shape, upward-pointing triangles represent pathways upregulated with age, and solid circles correspond to pathways downregulated with age. Selected pathways were chosen based on lowest adjusted p-values for each direction. (**C-D**) Age

differences in hippocampal gene expression for select pathways chosen based on overlap with the mouse microarray dataset, including pro-inflammatory genes and oxidative stress regulatory genes (C), and DNA damage repair and genome maintenance genes (D). Violin plots show the distribution of log_2-transformed expression values [log_2(TPM + 0.1)] for individuals aged 50-59, 60-69, and 70-79 years in the GTEx hippocampus dataset. Boxplots represent median and interquartile range (IQR), jittered points represent individual subjects, black circles indicate group means, and dashed lines connect mean values across age groups. Significant or nominal *post-hoc* differences are annotated using adjusted p-values from Tukey's HSD tests. Panels represent individual genes within the selected pathways.

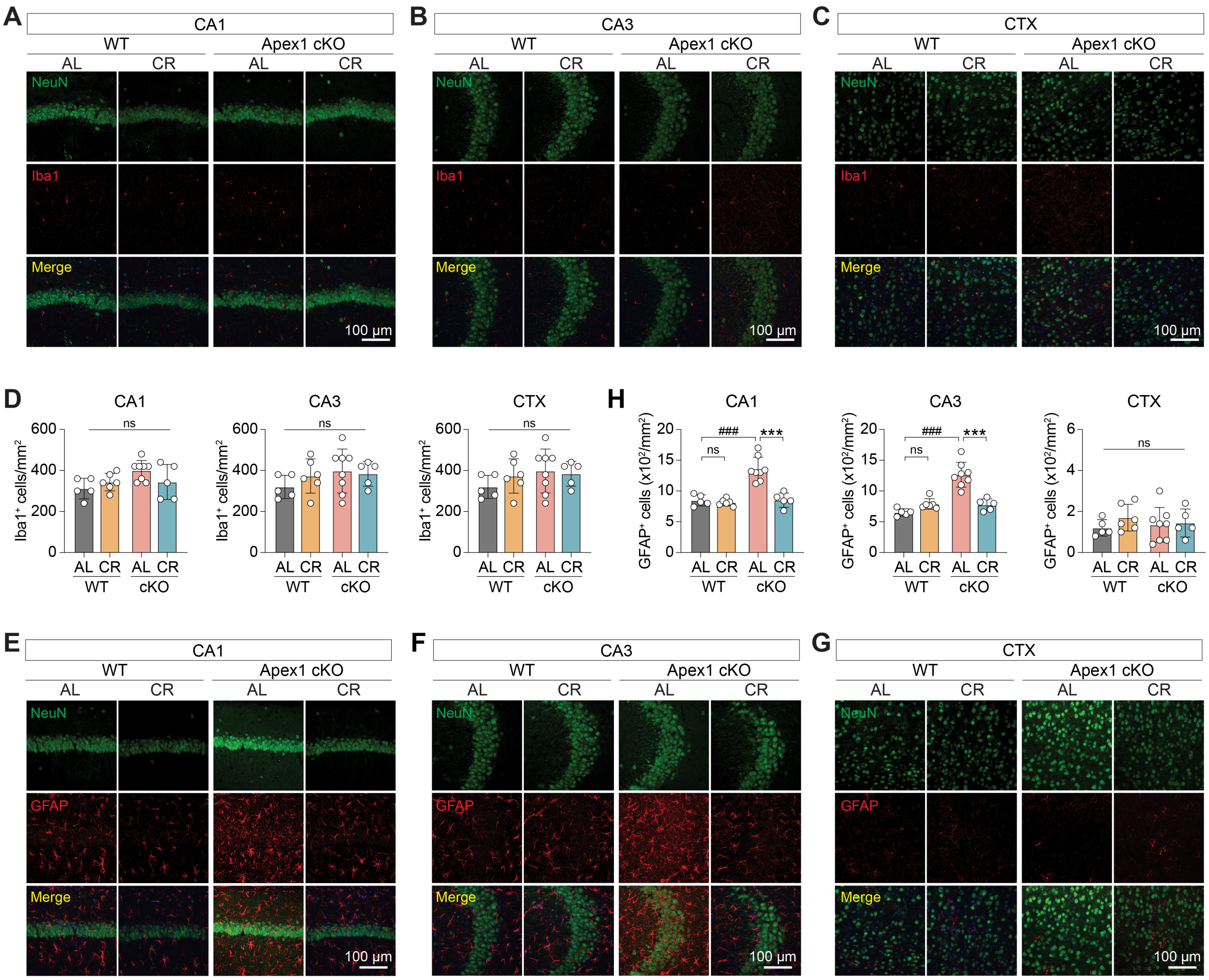

Supplementary Figure 10. Increased astroglia activation in the hippocampus of *Apex1* cKO mice. (A-D) Representative immunofluorescent images (A-C) and quantification (D) of Iba1$^+$ (red) cells in hippocampal CA1, CA3, and cortex (CTX) at week 48 (scale bar: 100 μm). **(E-H)** Representative immunofluorescent images (E-G) and quantification (H) of GFAP$^+$ (red) cells in hippocampal CA1, CA3, and CTX at week 48 (scale bar: 100 μm). Data are presented as mean ± SD. ns = no significant difference. ### $p < 0.001$, ***$p < 0.001$ vs. indicated group by two-way ANOVA/*Bonferroni* tests.

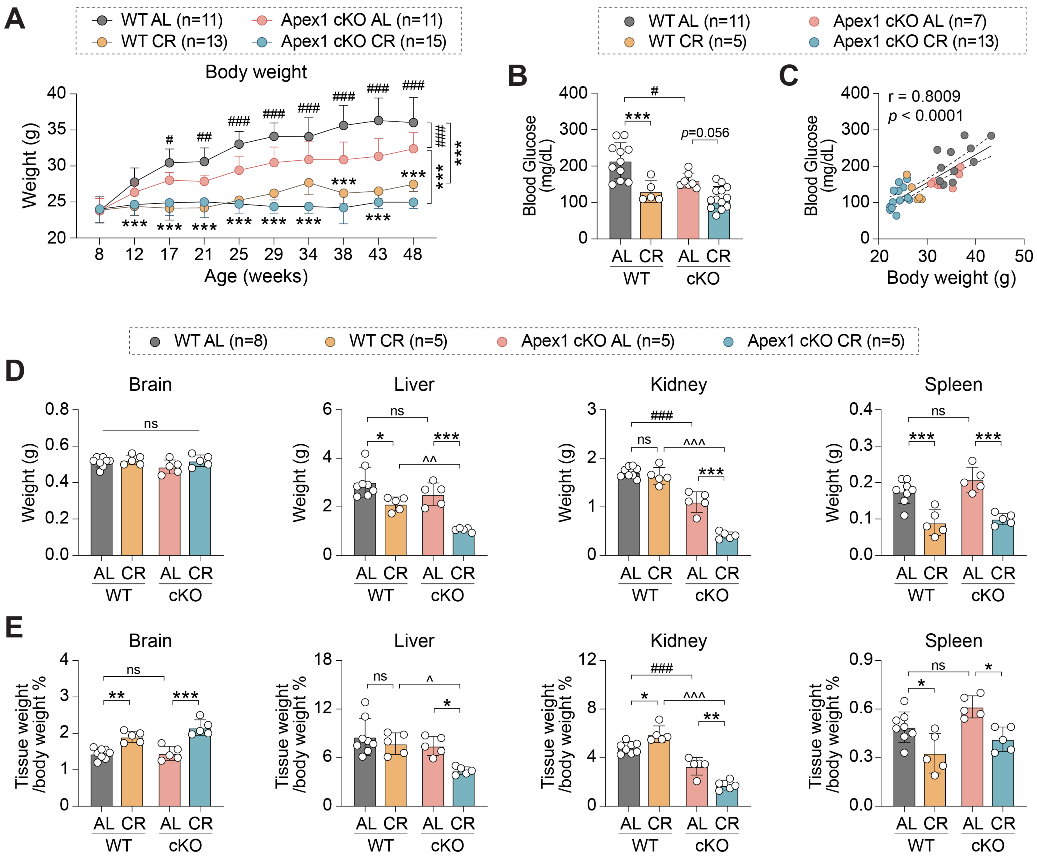

Supplementary Figure 11. Systemic alterations in *Apex1* cKO mice. (A) Body weight was measured longitudinally from 8-48 weeks of age at 4-week intervals. In the *Apex1* cKO CR group (n = 15), body weight measurements at 8 weeks were available for a subset of animals (n = 7), as measurements for the remaining mice (n = 8) began at 12 weeks. **(B)** Terminal blood glucose levels after 12 hours of fasting. **(C)** Pearson's two-tailed correlation analysis of bodyweight versus glucose levels at week 48. (**D-E**) Terminal tissue (brain, liver, kidney, and spleen) weights (D) and tissue weight/body weight % (E) at week 48. Data are presented as mean ± SD. ns = no significant difference. # p < 0.05, ### p < 0.001 *Apex1* cKO AL vs. WT AL; ^ p < 0.05, ^^ p < 0.01, ^^^ p < 0.001 *Apex1* cKO CR vs. WT CR; *p < 0.05, **p < 0.01, ***p < 0.001 CR vs. AL mice of the same genotype by repeated measures (A) or ordinary two-way ANOVA/*Bonferroni* tests (B, D-E).

Supplementary Tables

Supplementary Table 1. Animal recruitment and selection criteria

Experiment	Total number	Included number	Excluded number	Inclusion/Exclusion criteria
Body weight	52 mice:<br>WT AL n = 11<br>WT CR n = 13<br>Apex1 cKO AL n = 13<br>Apex1 cKO CR n = 15	50 mice:<br>WT AL n = 11<br>WT CR n = 13<br>Apex1 cKO AL n = 11<br>Apex1 cKO CR n = 15	2 mouse excluded:<br>Apex1 cKO AL (2)	Animals were excluded if their record lost at two or more time points.
Morris water maze	51 mice:<br>WT AL n = 11<br>WT CR n = 13<br>Apex1 cKO AL n = 13<br>Apex1 cKO CR n = 15	47 mice:<br>WT AL n = 11<br>WT CR n = 11<br>Apex1 cKO AL n = 11<br>Apex1 cKO CR n = 14	5 mice excluded:<br>WT CR (2)<br>Apex1 cKO AL (2)<br>Apex1 cKO CR (1)	The mice that cannot remain upright in the water or fail to find the visible platform were excluded from the experiment
Open Field	40 mice:<br>WT AL n = 11<br>WT CR n =13<br>Apex1 cKO AL n = 8<br>Apex1 cKO CR n = 8	37 mice:<br>WT AL n = 11<br>WT CR n =11<br>Apex1 cKO AL n = 7<br>Apex1 cKO CR n = 8	3 mouse excluded:<br>WT CR (2)<br>Apex1 cKO AL (1)	Randomly selected from animals for behavioral tests.<br>Animals that remain immobile for 50% or greater of the total trial duration were excluded from the analysis.
Novel object recognition	32 mice:<br>WT AL n = 8<br>WT CR n = 8<br>Apex1 cKO AL n = 8<br>Apex1 cKO CR n = 8	25 mice:<br>WT AL n = 6<br>WT CR n = 6<br>Apex1 cKO AL n = 6<br>Apex1 cKO CR n = 7	7 mice excluded:<br>WT AL (2)<br>WT CR (2)<br>Apex1 cKO AL (2)<br>Apex1 cKO CR (1)	Randomly selected from animals for behavioral tests.<br>Animals were excluded from the analysis if they failed to reach a minimum of 10 seconds of active exploration per object during either the familiarization or the test phase.
Golgi Staining	24 mice:<br>WT AL n = 6<br>WT CR n = 6<br>Apex1 cKO AL n = 6<br>Apex1 cKO CR n = 6	23 mice<br>WT AL n = 5<br>WT CR n = 6<br>Apex1 cKO AL n = 6<br>Apex1 cKO CR n= 6	1 mouse excluded:<br>WT AL (1)	One sample was lost during sample processing.
NeuN and 8-OHdG staining	30 mice:<br>WT AL n = 8<br>WT CR n = 7<br>Apex1 cKO AL n = 8<br>Apex1 cKO CR n = 7	30 mice:<br>WT AL n = 8<br>WT CR n = 7<br>Apex1 cKO AL n = 8<br>Apex1 cKO CR n = 7	No exclusion	Samples were randomly selected from different batches, irrespective of prior behavioral testing.
vGlu1 staining	22 mice:<br>WT AL n = 5<br>WT CR n = 6<br>Apex1 cKO AL n = 6<br>Apex1 cKO CR n = 5	22 mice:<br>WT AL n = 5<br>WT CR n = 6<br>Apex1 cKO AL n = 6<br>Apex1 cKO CR n = 5	No exclusion	Randomly selected from animals after LTP recording.
LTP (48-week-old mice)	41 mice:<br>WT AL n = 10<br>WT CR n = 8<br>Apex1 cKO AL n = 13<br>Apex1 cKO CR n = 10	35 mice:<br>WT AL n = 7<br>WT CR n = 7<br>Apex1 cKO AL n = 12<br>Apex1 cKO CR n = 9	6 mice excluded:<br>WT AL (3)<br>WT CR (1)<br>Apex1 cKO AL (1)<br>Apex1 cKO CR (1)	Randomly selected from animals after Morris water maze test.<br>LTP induction was considered valid if the initial HFS-induced fEPSP is at least 130% of baseline and the mean fEPSP slope between 40 and 60 min maintains ≥ 130% of baseline.
LTP (8-week-old mice)	18 mice:<br>WT AL n = 10<br>Apex1 cKO AL n = 8	18 mice:<br>WT AL n = 10<br>Apex1 cKO AL n = 8	No exclusion	The same criteria as above.
GAD, NeuN, Iba1, GFAP, PolB staining	24 mice:<br>WT AL n = 5<br>WT CR n = 6<br>Apex1 cKO AL n = 8<br>Apex1 cKO CR n = 5	24 mice:<br>WT AL n = 5<br>WT CR n = 6<br>Apex1 cKO AL n = 8<br>Apex1 cKO CR n = 5	No exclusion	Samples were randomly selected from different batches, irrespective of prior behavioral testing.

APE staining	16 mice:<br>WT AL n = 4<br>WT CR n = 4<br>Apex1 cKO AL n = 4<br>Apex1 cKO CR n = 4	16 mice:<br>WT AL n = 4<br>WT CR n = 4<br>Apex1 cKO AL n = 4<br>Apex1 cKO CR n = 4	No exclusion	Samples were randomly selected from different batches, irrespective of prior behavioral testing.
Microarray	16 mice:<br>WT AL n = 4<br>WT CR n = 4<br>Apex1 cKO AL n = 4<br>Apex1 cKO CR n = 4	15 mice:<br>WT AL n = 4<br>WT CR n = 4<br>Apex1 cKO AL n = 3<br>Apex1 cKO CR n = 4	1 sample excluded:<br>Apex1 cKO AL (1)	Based on the results of principal component analysis, one outlier was excluded.
qPCR	16 mice:<br>WT AL n = 4<br>WT CR n = 4<br>Apex1 cKO AL n = 4<br>Apex1 cKO CR n = 4	16 mice:<br>WT AL n = 4<br>WT CR n = 4<br>Apex1 cKO AL n = 4<br>Apex1 cKO CR n = 4	No exclusion	N/A
Blood Glucose	36 mice:<br>WT AL n = 11<br>WT CR n = 5<br>Apex1 cKO AL n = 7<br>Apex1 cKO CR n = 13	36 mice:<br>WT AL n = 11<br>WT CR n = 5<br>Apex1 cKO AL n = 7<br>Apex1 cKO CR n = 13	No exclusion	Samples were randomly selected from different batches, irrespective of prior behavioral testing.
Tissue weight	23 mice:<br>WT AL n = 8<br>WT CR n = 5<br>Apex1 cKO AL n = 5<br>Apex1 cKO CR n = 5	23 mice:<br>WT AL n = 8<br>WT CR n = 5<br>Apex1 cKO AL n = 5<br>Apex1 cKO CR n = 5	No exclusion	Samples were randomly selected from different batches, irrespective of prior behavioral testing.

Table 2. Primers used for qPCR

Gene	Oligo sequence
P21/Cdkn1a	Forward: CGAGAACGGTGGAACTTTGAC<br>Reverse: CCAGGGCTCAGGTAGACCTT
Txnip	Forward: CGAGTCAAAGCCGTCAGGAT<br>Reverse: TTCATAGCGCAAGTAGTCCAAAGT
Dmc1	Forward: GCATCAGATGGAGCTACTTGATT<br>Reverse: TCCACTCGAAAAAGTGCCATTAT
Sirt1	Forward: ATGACGCTGTGGCAGATTGTT<br>Reverse: CCGCAAGGCGAGCATAGAT
