# Supplementary material for: Caloric restriction decelerates premature aging and cognitive decline in mice with deficient DNA repair
Source: Commun Biol. 2026 May 8;9:960. doi: 10.1038/s42003-026-10182-3 (PMC13369994; doi:10.1038/s42003-026-10182-3)
Supplement: Supplementary file 3 — Description of Additional Supplementary Files [file 42003_2026_10182_MOESM3_ESM.pdf]

Description of Additional Supplementary Files:

File: Supplementary Data 1

Description: Summary of genotype × diet interaction effects across behavioral and neurobiological measures

File: Supplementary Data 2

Description: Comprehensive statistical analysis of all experimental measures – main figures

File: Supplementary Data 3

Description: Comprehensive statistical analysis of all experimental measures – supplementary figures

File: Supplementary Data 4

Description: The source data behind the graphs in the paper
