# Supplementary material for: Caloric restriction decelerates premature aging and cognitive decline in mice with deficient DNA repair
Source: Commun Biol. 2026 May 8;9:960. doi: 10.1038/s42003-026-10182-3 (PMC13369994; doi:10.1038/s42003-026-10182-3)
Supplement: Supplementary file 8 — Reporting Summary [file 42003_2026_10182_MOESM8_ESM.pdf]

nature portfolio

Corresponding author(s): Jun Chen; Yejie Shi

Last updated by author(s): Feb 18, 2026

Reporting Summary

Nature Portfolio wishes to improve the reproducibility of the work that we publish. This form provides structure for consistency and transparency in reporting. For further information on Nature Portfolio policies, see our Editorial Policies and the Editorial Policy Checklist.

Statistics

For all statistical analyses, confirm that the following items are present in the figure legend, table legend, main text, or Methods section.

n/a	Confirmed	
☐	☒	The exact sample size (*n*) for each experimental group/condition, given as a discrete number and unit of measurement
☐	☒	A statement on whether measurements were taken from distinct samples or whether the same sample was measured repeatedly
☐	☒	The statistical test(s) used AND whether they are one- or two-sided<br>*Only common tests should be described solely by name; describe more complex techniques in the Methods section.*
☐	☒	A description of all covariates tested
☐	☒	A description of any assumptions or corrections, such as tests of normality and adjustment for multiple comparisons
☐	☒	A full description of the statistical parameters including central tendency (e.g. means) or other basic estimates (e.g. regression coefficient) AND variation (e.g. standard deviation) or associated estimates of uncertainty (e.g. confidence intervals)
☐	☒	For null hypothesis testing, the test statistic (e.g. *F*, *t*, *r*) with confidence intervals, effect sizes, degrees of freedom and *P* value noted<br>*Give P values as exact values whenever suitable.*
☒	☐	For Bayesian analysis, information on the choice of priors and Markov chain Monte Carlo settings
☒	☐	For hierarchical and complex designs, identification of the appropriate level for tests and full reporting of outcomes
☒	☐	Estimates of effect sizes (e.g. Cohen's *d*, Pearson's *r*), indicating how they were calculated

Our web collection on statistics for biologists contains articles on many of the points above.

Software and code

Policy information about availability of computer code

Data collection	Microarray analyses were performed using the Affymetrix Clariom S mouse array. The chip was scanned using the GeneArray 3000 scanner.
Data analysis	First-level image analysis was performed using Affymetrix Expression Console. Detected probes were analyzed using Transcriptome Analysis Console (Affymetrix).

For manuscripts utilizing custom algorithms or software that are central to the research but not yet described in published literature, software must be made available to editors and reviewers. We strongly encourage code deposition in a community repository (e.g. GitHub). See the Nature Portfolio guidelines for submitting code & software for further information.

Data

Policy information about availability of data

All manuscripts must include a data availability statement. This statement should provide the following information, where applicable:

- Accession codes, unique identifiers, or web links for publicly available datasets
- A description of any restrictions on data availability
- For clinical datasets or third party data, please ensure that the statement adheres to our policy

The source data related to all figures are presented in Supplementary Data. All other raw data generated in this study are available from the corresponding author upon request.

Research involving human participants, their data, or biological material

Policy information about studies with human participants or human data. See also policy information about sex, gender (identity/presentation), and sexual orientation and race, ethnicity and racism.

Reporting on sex and gender	There were no human participants in this study.
Reporting on race, ethnicity, or other socially relevant groupings	There were no human participants in this study.
Population characteristics	There were no human participants in this study.
Recruitment	There were no human participants in this study.
Ethics oversight	There were no human participants in this study.

Note that full information on the approval of the study protocol must also be provided in the manuscript.

Field-specific reporting

Please select the one below that is the best fit for your research. If you are not sure, read the appropriate sections before making your selection.

☒ Life sciences ☐ Behavioural & social sciences ☐ Ecological, evolutionary & environmental sciences

For a reference copy of the document with all sections, see nature.com/documents/nr-reporting-summary-flat.pdf

Life sciences study design

All studies must disclose on these points even when the disclosure is negative.

Sample size	Although formal power calculations were not performed, each group included a robust sample size, and data were consistently collected from at least four independent replicates per assay.
Data exclusions	A single outlier sample in the microarray dataset was identified and excluded based on principal component analysis (PCA). In the visible platform test, animals that failed to find the platform were excluded from further analyses. In the LTP experiment, LTP induction was considered valid if the mean fEPSP slope between 40 and 60 min post-tetanus was ≥130% of baseline. If the average fEPSP slope during this period fell below baseline, LTP induction was considered a failure. Slices failing either criterion were excluded a priori from analyses. All LTP data reported were filtered according to this standard.
Replication	Each experiment was performed with at least four biological replicates (mice), each representing an independent sample, and all replication attempts were successful.
Randomization	Mice were randomly assigned to experimental groups with a lottery drawing box.
Blinding	Experiments were conducted and analyzed in a blinded manner whenever feasible.

Reporting for specific materials, systems and methods

We require information from authors about some types of materials, experimental systems and methods used in many studies. Here, indicate whether each material, system or method listed is relevant to your study. If you are not sure if a list item applies to your research, read the appropriate section before selecting a response.

Materials & experimental systems

n/a	Involved in the study	
☐	☒	Antibodies
☒	☐	Eukaryotic cell lines
☒	☐	Palaeontology and archaeology
☐	☒	Animals and other organisms
☒	☐	Clinical data
☒	☐	Dual use research of concern
☒	☐	Plants

Methods

n/a	Involved in the study	
☒	☐	ChIP-seq
☒	☐	Flow cytometry
☒	☐	MRI-based neuroimaging

Antibodies

Antibodies used	rabbit anti-NeuN (EMD Millipore), mouse anti-APE1 (Abcam), rat anti-vGlut1 (NeuroMab), rabbit anti-GAD (EMD Millipore), goat anti-Iba1 (Abcam), goat anti-GFAP (Abcam), mouse anti-8-OHdG (Abcam), and rabbit anti-PolB (Acris).
Validation	These antibodies have been validated both by the manufacturer and through publications, as they are widely used antibodies. Specific citations can be found on each manufacturer's website.

Animals and other research organisms

Policy information about studies involving animals; ARRIVE guidelines recommended for reporting animal research, and Sex and Gender in Research

Laboratory animals	Apex1(flox/flox) mice and CamkIIa-Cre mice (both on C57BL/6 background) were used to breed the Apex1 cKO mice and WT control mice used in this study. Animals were used at 8 and 48 weeks of age.
Wild animals	No wild animal was used.
Reporting on sex	Only male mice were used in this study.
Field-collected samples	No field-collected sample was used.
Ethics oversight	All animal procedures were approved by the University of Pittsburgh Institutional Animal Care and Use Committee and performed in accordance with the National Institutes of Health Guide for the Care and Use of Laboratory Animal.

Note that full information on the approval of the study protocol must also be provided in the manuscript.

Plants

Seed stocks	*Report on the source of all seed stocks or other plant material used. If applicable, state the seed stock centre and catalogue number. If plant specimens were collected from the field, describe the collection location, date and sampling procedures.*
Novel plant genotypes	*Describe the methods by which all novel plant genotypes were produced. This includes those generated by transgenic approaches, gene editing, chemical/radiation-based mutagenesis and hybridization. For transgenic lines, describe the transformation method, the number of independent lines analyzed and the generation upon which experiments were performed. For gene-edited lines, describe the editor used, the endogenous sequence targeted for editing, the targeting guide RNA sequence (if applicable) and how the editor was applied.*
Authentication	*Describe any authentication procedures for each seed stock used or novel genotype generated. Describe any experiments used to assess the effect of a mutation and, where applicable, how potential secondary effects (e.g. second site T-DNA insertions, mosiacism, off-target gene editing) were examined.*
