# Supplementary material for: Caloric restriction decelerates premature aging and cognitive decline in mice with deficient DNA repair
Source: Commun Biol. 2026 May 8;9:960. doi: 10.1038/s42003-026-10182-3 (PMC13369994; doi:10.1038/s42003-026-10182-3)
Supplement: Supplementary file 1 — Transparent Peer Review file [file 42003_2026_10182_MOESM1_ESM.pdf]

nature portfolio

Peer Review File

Caloric restriction decelerates premature aging and cognitive decline in mice with deficient DNA repair

Corresponding Author: Professor Jun Chen

This file contains all reviewer reports in order by version, followed by all author rebuttals in order by version.

Version 0:

Reviewer comments:

Reviewer #1

(Remarks to the Author)
The study by Wei et al. characterizes the impact of caloric restriction (CR) on brain function in conditional ko mice lacking the DNA repair protein APEX1 in forebrain neurons. They show that CR restores age-dependent spatial memory impairment, LTP deficit and structural properties of hippocampal neurons in APEX1 cKO mice. Moreover, in these mice, CR protected from DNA damage accumulation which seems to be mediated by upregulation of DNA repair genes and antioxidant defense genes. Overall, this is an interesting study further supporting a beneficial role of CR on brain health. The manuscript is written in a clear and concise manner, however, there are some points that the authors should address.

1. The description of the APEX1 cKO mice is very limited. For instance, timing of tamoxifen is not indicated. It should also be specified what WT mice are.
2. In the open field test, Apex1 cKO CR mice seem to travel substantially less distance already at week 8 just when CR is initiated (Fig 2C). What is causing this difference?
3. The authors found no difference in Apex1 expression with CR, it would however be interesting to see its activity. Are levels of AP sites changing upon ko and with intervention?
4. In Fig 4, no comparison of LTP between WT AL and Apex cKO AL at 48 weeks is shown, thus it is not possible to conclude that Apex1 disrupts LTP (Line 232). Similar, in Line 235 the authors refer to a LTP deficit in aged WT and Apex1 cko mice, but no graph shows this age effect.
5. Fig S7D-E shows increased number of GFAP+ cells in Apex1 cko. How can a neuron-specific ko affect the astrocyte level. A discussion along this line would be beneficial.
6. Similar to point 5, the authors show a significant effect of Apex1 cKO in neurons on overall bodyweight, blood glucose level or kidney weight (Fig S8). How can that be explained?
7. In FigS9, it would be beneficial to show a graph with number of DEGs in the different groups for easier comparison. Are gene expression changes in Apex1 cKO rescued after CR intervention and therefore similar to WT CR mice? I would also suggest moving gene expression data to a main figure.
8. Although interesting, the correlation analysis with human RNAseq data looks at brain regions that are not necessarily relevant for this study. How about analyzing publicly available datasets that cover the hippocampus (e.g. GTEx -Genotype-Tissue Expression Project)?
9. In the discussion in line 330-334, it is not correct to state that the current study is filling a missing gap between Apex1 and brain aging. There are recent studies examining this relationship among a previous study by the authors themselves (Zhu et al., 2022 - DOI: 10.14336/AD.2022.0331 and Li et al.,2018 – DOI: 10.1093/nar/gky326).

Minor comments:

10. In Line 169-171 and Line 247, it is not correct to associate Apex1 with superior cognitive function. CR rather restores memory function in Apex1 cKO mice but not above the WT CR level.
11. The abbreviation AL is not introduced and should be used consistently in the study - AL vs Ad-lib in figures.
12. Fig S4C - a graph legend is missing
13. Line 280 - number of DEGs in superscript 524
14. Line 300 – it should be “selected genes”

Reviewer #2

(Remarks to the Author)
The accumulation of DNA lesions and mutations over lifetime, partially caused by an excess of reactive oxygen species, has been considered to be one central mechanism underlying natural aging. The apurinic/apyrimidinic (AP) endonuclease (APE, aka APEX) is an enzyme involved in DNA base excision repair (BER) while APE1 (aka APEX1) is supposed to play a more crucial role compared to APE2 (aka APEX2). Given that the full deletion of APE1 leads to embryonic lethality in mice, it became common practice to generate cre-flox mice for experimental use. In the present study, Wei and co-workers leveraged the conditional APE1 knockout (ko) mice under the control of the Camk2α promotor, a model they established in their previous work, to investigate whether 30% caloric restriction (CR) over 40 weeks would attenuate detrimental effects induced by conditional APE1 ko (cAPE1 ko) in a neuron specific context. Main findings included (1) CR boosted locomotion independent of genotype, (2) CR improved spatial learning and memory independent of genotype, (3) CR partially rescued the shortening of dendritic length and reduction in branch points in CA1 neurons of cAPE1 ko mice, (4) CR elevated the CA1 dendritic spine density independent of genotype, (5) CR incremented hippocampal long-term potentiation independent of genotype. Although I acknowledge the workload put into this study, I disagree with the authors in terms of data interpretation and conclusion. Furthermore, the data presentation in its current form appears to be less ideal, difficult to interpret and confusing to some extent. Finally, fundamental flaws in the experimental design exist which cannot be compensated by adding more data or re-analysis of the existing data, at least in my personal opinion. Please find my detailed comments below.

General remarks for data presentation and statistics:
First of all, it might be favorable to rearrange the order as well as the structure of the behavior datasets such that panels derived from one particular type of experiment are portrayed as stand-alone figures. Content wise, one could consider to start the results section by describing the outcomes of the open field test, followed by the two memory-related paradigms (MWZ and novel object recognition).

Secondly, it could be more meaningful to show the combined data derived from wt and cAPE1 ko mice in the respective panels (alike in Fig. 3E,F or Supp. Fig. 1E), instead of the current genotype-split variant in a high number of figures and supplementary figures. The side-by-side data presentation would allow direct comparisons to visually estimate the influence and interaction of within and between factors such that the reader is not forced to jump between figure panels back and forth. As an additional advantage, the manuscript would be cleansed to a certain extent by reduction of total figure panels.

In the M+M section, the authors stated that either the ordinary 2-way ANOVA or a 2-way ANOVA featuring repeated measures were applied for statistical analyses. However, the analysis of a number of figure panels (for instance Fig. 3E,F or Supp. Fig. 1E) would demand for the usage of a 3-way ANOVA based on the factors age, genotype and dietary restriction. Even if the authors insist to stick with the 2-way ANOVA based statistics, it would be great to display the p-values derived from the 2-way ANOVA analyses directly in the according figure panels to show whether a main age effect, a main genotype effect and/or an age x genotype interaction exist. In case this would not be possible due to space limitation, one could present those numbers in an organized table (e.g. in excel format) as a supplementary file.

Methodological remarks:
The general validity of the entire experimental approach needs to be questioned due to the following reasons.

To estimate food intake and to calculate the daily food allotment assigned to the CR mice, the authors measured the food consumption of AL-fed mice prior to initiation of the study. However, the food intake was not re-accessed and re-adjusted for the CR animals at later time points. Since the dynamic nature of murine food intake over lifetime is well documented in numerous reports, the assumption that the food intake of a mouse remains constant over the duration of almost a year appears to be inept and represents one major flaw of the current study.

Furthermore, the authors opted to group-house AL-fed animals while CR mice were individually housed, as stated in the M+M section. Keeping the groups of animals at diverging conditions impedes a proper interpretation of the experimental outcomes given that housing condition is known to affect behavior, learning and memory.

Behavior tests were repetitively administered based on a 4-weeks interval from 8 weeks of age onwards. In line with previous studies, animals familiar with a given test perform substantially better at subsequent occasions. Accordingly, the outcomes derived from the behavior tests rendered the ability to retain memory over a long period of time, rather than memory formation over a few days. I´m not aware that the authors made this discrimination explicitly.

Remarks to results and conclusion:
Throughout the entire manuscript, the authors emphasized on the validity of cAPE1 ko mice as a model of premature aging without demonstrating it clearly. The only piece of convincing evidence I recognized was the abundance of 8-OHdG in the CA1 region shown in Fig. 3K. As outlined above, the differences in MWZ performance between cAPE1 ko and wt mice arose from lack of memory retention starting from 17 weeks onwards, while similar learning curves were found when initially tested at 8 weeks of age (Fig. 1C,D). Exploratory activity in a novel environment shown as total distance moved in the open field test did not differ between wt+AL and cAPE1 ko+AL mice, while general elevation was detected after long-term CR treatment (Fig. 2C). Analyses of dendritic length and branch points of CA1 neurons, shown in Fig. 3C,D, seem to be less appropriate given that each neuron was apparently treated as an individual sample, which artificially yielded a very high sample size. It would be more meaningful to calculate the average of each biological individual and run the statistics accordingly.

In their previous work published end 2022 (PMID36465182), density of vGlut in the cortex, CA1 and CA3 regions were significantly decreased in cAPE1 ko mice. This phenotype was not successfully reproduced in the current study (Fig. 4H, Supplementary Fig. 5B). Similarly, density of GAD was significantly decreased in cAPE1 ko mice in their 2022 publication, but was not distinguishable between wt+AL and cAPE1 ko+AL mice in the current study (Fig. 4I, Supplementary Fig. 6B). These discrepancies raise two issues. Firstly, the behavior phenotypes observed in the current study are unrelated to vGlut and GAD levels such that the underlying molecular base remains obscure. Secondly, the failure to validate phenotypes previously established in the same lab potentially indicate that either the model is volatile from batch to batch or alternatively the phenotypes of the model may vary depending on the actual age stage. Both scenarios are worrisome and don´t support the validity of the mouse model.

Remarks to figures and supplementary figures:
Abbreviations should be unified throughout the main text of the manuscript, the figures and legends. Apex1 cKO is used in most figure panels, while APE1 cKO is also present in some figure panels. For instance in Fig. 3, both versions are included. Although I understand that the former refers to the gene and the latter refers to the protein, it might be meaningful to use Apex1 cKO exclusively given that this abbreviation was introduced by the authors in the abstract.

The bar chart in Fig.2F does not correspond to data shown in the left and middle panel. In the left panel, the preference index of the wt+AL mice is slightly higher than the value displayed for the wt+CR mice at 48 weeks of age. The opposite is shown in the bar chart on the right. Same applies for Supplementary Fig. 2B.

Sample size of the APE1 cKO group shown in Supplementary Fig. 1A does not align with the other panels of this figure, although the data was obtained from the same animals. While the APE1 cKO group shows 12 data points, 14 data points are indicated in the other panels.

It is interesting that conditional knockout of APE1 in the central nervous system specifically reduced kidney weight while the brain and other peripheral organs remained unaffected. Unfortunately, the authors did not follow-up on this finding.

The data presentation related to the microarray-based analyses could be further refined. Six groups (wt young, wt AL, wt CR, Apex1 cKO young, Apex1 cKO AL, Apex cKO CR) with unknown sample sizes and age structure have been analyzed, as stated in the M+M section. The current data presentation including only four groups prohibit any conclusion related to age (no young animals included). It remains unclear to me why the authors opted to perform multiple pairwise comparisons instead of showing the DEGs with a main effect of genotype, a main effect of CR and/or a genotype x CR interaction in a comprehensive manner, e.g. using a Venn-diagram. Among the DEGs of interest shown in Supplementary Fig. 9B, cdkn1a (encoding p21) is a prominent senescence associated gene and its transcription is elevated in the Apex1 cKO mice and further exacerbated by CR, indicating an obvious pro-aging profile by the applied dietary paradigm in this specific case.

Remarks to figure legends and supplementary figure legends:
Scale bar numbers shown in Fig. 3A (400 µm, 200 µm, 100 µm) don´t align with the figure legend (50 µm, 100 µm, 50 µm).

In lines 141-143, the authors stated that “These memory trials were confirmed by testing other parameters, such as the latency to first entry into the target zone and the time to cross the platform [Fig. S1 C, D].”. In contrast, “Time to reach the platform area during the learning phase of the water maze test.” is stated in the legend of Supplementary Fig. 1C-D.

Reviewer #3

(Remarks to the Author)
The study by Wei et. al presents the effects of caloric restriction on aging and cognitive decline in Apex1 KO mice. The work is very interesting and provides a perspective on the possible therapeutic effects of CR. Manuscript is clearly written, appropriate statistical tests have been used and the conclusions are well supported. However, one important limitation that should be addressed, is the lack of information regarding the sex of the animals used. The sex variable is not included in the experimental design or data analysis. Incorporating sex as a biological variable would significantly strengthen the study, as it could reveal potential sex-specific differences in the metabolic response to CR and/or the impact of Apex1 deficiency.

SPECIFIC COMMENTS
Line 83: There is a more recent Lopez-Otin et al. paper that could be cited here, defining 12 hallmarks of aging. Perhaps it would be more suitable to cite more recent literature
Line 126: I think it should be „Apex1 cKO CR mice displayed faster swim speeds than cKO AL and WT CR“ – as it is labeled on Fig S1 B
Figure 1 C, D: please add 'day' to indicate what the numbers 1-4 refer to, as in Fig S1 E, to make it more clear
Figure 1 F, G: X-axis could be just labeled Age/weeks, no need for additional line
Lines 159-160: Please rewrite to make it clear that the effect that waned at 48 weeks is between cKO AL and CR mice, not between cKO CR and WT AL (it makes no sense to compare those two)
Figure 2: label X axis Age/weeks and consider re-labeling every panel in C, D with different letter (so to have 6 panels labeled, instead of just 2)
Figure S2: label X axis Age/weeks
Figure 3F: it is not clear to which groups statistical significance refers to
375-377: please state the paper you are referring to as Author, et al., or put the reference number in that sentence to make it more clear

Discussion: Please state the limitations of the study - biological sex of the animals used

Version 1:

Reviewer comments:

Reviewer #1

(Remarks to the Author)
The manuscript has been revised thoroughly, and my concerns have been adequately addressed.

Reviewer #2

(Remarks to the Author)
I acknowledge the effort the authors invested in strengthening the revised manuscript by accommodating my remarks. While many issues have been addressed appropriately, some aspects remain worthy of discussion.

Point 1:
Based on the data presented in the original manuscript, I interpreted the effects induced by CR as a mixture of genotype-independent (e.g. locomotion, learning and memory, CA1 dendritic spine density, hippocampal LTP) and genotype-dependent (e.g. rescued shortening of dendritic length and reduction in branch points in CA1 neurons) phenotypes. The authors obviously disagree in this regard and emphasize that the genotype-dependent outcomes are more prominent. Although I understand that this discussion may rely on individual perspective, such that right or wrong simply does not exist, I would like to explain my view of the data by walking through all relevant panels.

In the open field test (formerly Fig. 2A-D, now Fig 1B-G), CR significantly elevates exploratory locomotion in both WT and Apex1 cKO mice. At 48 weeks of age, total distance traveled is higher (Fig. 1E) and the time spent in the corner regions is decreased in the CR groups, independent of genotype (Fig. 1G). In Table 1 / Fig. 1E, the parameter “Total distance traveled (48 weeks)” shows a non-significant comparison between cKO-AL vs. cKO-CR (p=0.1394) after posthoc correction. However, in Fig. 1E, the authors depicted a statistically significant outcome (p=0.0331) derived from a separate two-tailed t-test, which is not legitimate. Therefore, the overall CR effect on exploratory locomotion appears to be genotype-independent.

In the Morris water maze test (formerly Fig. 1, now Fig. 2), CR significantly improves escape latency in both wt and Apex1 cKO mice (Fig. 2A). The authors might argue that the magnitude of change at 48 weeks of age is substantially greater in the Apex1 cKO mice than in WT animals – which is true. However, this might be explained by a ceiling effect, because the performance of the mice has already reached its optimum after numerous rounds of training and further reductions of escape latency to less than a few seconds appear less likely. As shown in the bottom panels of Fig. 2C (48 weeks old), WT animals - with or without CR treatment - head straight toward the platform zone without hesitation once they enter the test arena. Regarding quadrant occupancy during the probe trial shown in Fig. 2D, I admit that the outcome of this measure resembles a true interaction between CR and genotype. In the lower panel of Fig. 2E (48 weeks old), statistical significance is depicted for the comparisons WT+AL vs. cKO-AL (###) and cKO-AK vs. cKO-CR (**). However, as shown in Table 1, the comparison WT-AL vs. cKO-AL yielded a p-value of 0.0172 (#) and the comparison cKO-AK vs. cKO-CR failed to reach statistical significance (p=0.9109). Please correct Fig. 2E accordingly.

In the original submission, total spine density was significantly increased by CR, independent of genotype (formerly Fig. 3H right panel, now Fig. 3F right panel). After applying the 2-way ANOVA, the revised data indicates a genotype x diet interaction while the main diet effect is not significant (p=0.0926). Based on the statistical outcome, I agree with the authors that CR differentially affects this trait in a genotype-dependent manner.

Hippocampal LTP is another case in which a disagreement exists. Based on the data enclosed in the original submission (formerly Fig. 4A-F), the fEPSP slope was modified by CR in both WT and Apex1 cKO mice to a similar extent, leading to my interpretation that the effect was genotype-independent. Consistent with my observation, in lines 224-226 of the original submission, the authors stated that “The hippocampal slices from 48-week-old WT CR mice showed superior LTP, with higher fEPSPs slopes than WT AL mice [Fig. 4A-C].” Curiously, in the revised manuscript, the fEPSP slope of the WT-AL mice in Fig. 4B substantially differs from the graph shown in the original submission, whereas the other fEPSP slopes remained unmodified. The marked difference in the fEPSP slopes between WT-AL and WT-CR animals, shown in Fig. 4C of the original submission, disappeared and became statistically non-significant in the revised manuscript. This data replacement demands a logical explanation.

Point 5:
I questioned the validity of the CR treatment since a highly unusual CR protocol (group housing combined with determination of food consumption only once prior to initiation of CR) was used in this study. But the authors disagree and claim that their experimental design represents the standard approach in long-term CR studies. Furthermore, the technical feasibility of dynamically adjusting food intake was questioned by the authors and two related publications were cited, which ironically contradict the authors´ viewpoint. For instance, the Turturro et al. study, referenced by the authors, stated that “Animals were housed individually.” and “Food consumption was monitored weekly for a cohort of animals. These consumptions were used to determine the ration to be fed to the CR groups in the studies.” – which is precisely how a long-

term CR paradigm should be administered in mice.

As shown in previous studies, food consumption in mice gradually increases from the juvenile stage to middle age, making continuous adjustment of the food allotment a crucial feature of any long-term CR study when initiated at young age. Determining food consumption only once prior to CR administration at young age bears the risk of underestimating the degree of CR at later ages. Therefore, I suggest that the authors acknowledge this limitation in their experimental design by adding a clear statement in the Discussion section noting that the CR protocol applied in the current study differs from the standard approach and the results should also be interpreted accordingly.

Point 9:
I appreciate that the authors provided additional evidence supporting the validity of Apex1 cKO as a valuable tool to study brain aging. On the other hand, simply referring to differences in the age of assessment does not convincingly explain the failure to reproduce changes in vGlut and GAD expression levels upon conditional Apex1 deletion between studies, especially since they were conducted by the same lab. Additionally, the authors speculate whether “differences in age, cohort, or housing conditions” might contribute to the discrepancies observed between studies. Such volatility to potential batch effects may complicate the data interpretation and impede reliable reproduction of phenotypes. I suggest that the authors add a sentence to the Discussion stating that – in contrast to their previous study (Zhu et al., 2022) – expression levels of vGlut and GAD do not differ between WT and Apex1 cKO mice at 48 weeks of age and that this observation warrants further investigation.

Reviewer #3

(Remarks to the Author)
The authors have addressed all my concerns and the quality of manuscript has been improved. In my opinion, the revised manuscript is ready for publication.

Open Access This Peer Review File is licensed under a Creative Commons Attribution 4.0 International License, which permits use, sharing, adaptation, distribution and reproduction in any medium or format, as long as you give appropriate credit to the original author(s) and the source, provide a link to the Creative Commons license, and indicate if changes were made.
In cases where reviewers are anonymous, credit should be given to 'Anonymous Referee' and the source.
The images or other third party material in this Peer Review File are included in the article’s Creative Commons license, unless indicated otherwise in a credit line to the material. If material is not included in the article’s Creative Commons license and your intended use is not permitted by statutory regulation or exceeds the permitted use, you will need to obtain permission directly from the copyright holder.
To view a copy of this license, visit https://creativecommons.org/licenses/by/4.0/

The authors thank all three reviewers for their positive comments on our study and insightful and constructive suggestions that have helped tremendously improve the manuscript. We have revised the manuscript according to the suggestions, and all major changes are highlighted in the revised manuscript. Below are our point-by-point responses to the critiques. We hope the reviewers find that this revision has been satisfactorily improved.

Reviewer #1

We thank Reviewer #1 for the positive comments on the clarity of our manuscript and the significance of our findings regarding the beneficial effects of caloric restriction on brain function, as well as for the insightful suggestions that have helped us improve the manuscript.

1. “The description of the APEX1 cKO mice is very limited. For instance, timing of tamoxifen is not indicated. It should also be specified what WT mice are.”

Response: We thank the reviewer for this important clarification. We have now expanded the Methods section (Lines 506-510) to provide details on how the *Apex1* cKO mice were generated. Specifically, we used a constitutive Camk2a-Cre driver line that does not require tamoxifen induction. In addition, we now specify that the WT control mice were hemizygous CaMKIIa-Cre+/- mice of the same sex and age.

2. “In the open field test, Apex1 cKO CR mice seem to travel substantially less distance already at week 8 just when CR is initiated (Fig 2C). What is causing this difference?”

Response: Thank you for this insightful comment. After auditing the raw data, we discovered that in the original submission the group colors for the week-8 open-field distance panel were inadvertently flipped, which created the appearance that Apex1 cKO CR mice traveled less than cKO AL mice. We have corrected the figure and legend in the revision (original Fig. 2C is now Fig. 1D-E). The corrected plots show that at week 8 the ***Apex1* cKO CR** group traveled **more** than the **cKO AL** group. We speculate that the initiation of CR activated the hypothalamic-pituitary-adrenal axis in Apex1 cKO mice, and that they display food-anticipatory and foraging activity [1,2]. Studies in the literature suggest that CR induces stress in mice, resulting in higher levels of corticosterone and anxiety-like behaviors [3,4]. The open field test was delayed for a few days after the initiation of CR and, therefore, this behavior could be captured early in Apex1 cKO CR mice. In response to this concern, we have now added dot plots for all behavior tests at 8 weeks (Fig. 1E, Fig. 1G, Fig. 1K, Fig. 2B, Fig. 2E, Fig. S3B, Fig. S3D, Fig. S4B), but only the open field test detected significant changes in selective parameters in Apex1 cKO after the initiation of CR at 8 weeks. Detailed statistical data are all listed in Supplementary Tables S1 and S2.

3. "The authors found no difference in Apex1 expression with CR, it would however be interesting to see its activity. Are levels of AP sites changing upon ko and with intervention?"

Response: We thank the reviewer for this thoughtful suggestion. In our previous study (Stetler et al., 2016)[5], we quantitatively measured AP site levels and found that young adult *Apex1* cKO mice exhibited a greater than 50% increase in average number of AP sites in the brain compared to age-matched wild-type controls in young adult mice (see Fig. 3A in that publication). Although we did not assess AP site levels in aged animals in the current study (it requires a whole cohort of mice specifically and only for this purpose), we agree that evaluating AP site accumulation and its modulation by CR in older mice would provide valuable mechanistic insight. We have now mentioned this as a direction for future research in the revised Discussion (Lines 468-474).

4. "In Fig 4, no comparison of LTP between WT AL and Apex cKO AL at 48 weeks is shown, thus it is not possible to conclude that Apex1 disrupts LTP (Line 232). Similar, in Line 235 the authors refer to a LTP deficit in aged WT and Apex1 cko mice, but no graph shows this age effect."

Response: Thank you for these helpful comments. We have now added graphs (Fig. S6) that show the direct comparison of LTP between WT AL and Apex cKO AL at 8 and 48 weeks, respectively, and between WT AL 8 weeks and WT AL 48 weeks and cKO AL 8 weeks and cKO AL 48 weeks. The Results have been updated accordingly (Lines 223-231).

5. "Fig S7D-E shows increased number of GFAP+ cells in Apex1 cko. How can a neuron-specific ko affect the astrocyte level. A discussion along this line would be beneficial."

Response: We thank the reviewer for this insightful comment. Although the *Apex1* deletion is restricted to forebrain neurons, our data suggest that neuronal loss of *Apex1* results in secondary neurotoxic effects that impact surrounding glial cells. At 48 weeks, *Apex1* cKO mice exhibit substantial neuronal injury, including dendritic degeneration, DNA damage, and synaptic impairment (Figures 3 and S7). As major support cells, astrocytes are highly responsive to neuronal stress and damage, undergoing reactive gliosis and increased GFAP expression. Thus, we believe that the elevated GFAP+ cell numbers in *Apex1* cKO mice likely reflect an astrocytic response to progressive neurodegeneration. This interpretation is supported by our finding that caloric restriction (CR) mitigated neuronal damage in *Apex1* cKO mice and correspondingly reduced GFAP+ astrocytosis, particularly in hippocampal CA1 and CA3. These data suggest that CR blunts neuroinflammation, at least in part by preserving neuronal integrity and preventing secondary glial activation. We have added this discussion to the revised manuscript (Lines 447-456).

6. "Similar to point 5, the authors show a significant effect of Apex1 cKO in neurons on overall bodyweight, blood glucose level or kidney weight (Fig S8). How can that be explained?"

Response: We appreciate the reviewer's observation and agree that this raises an interesting point. Although the Apex1 deletion is neuron-specific, accumulating evidence suggests that chronic neuronal

stress and neurodegeneration can contribute to systemic aging phenotypes through altered neuroendocrine signaling, energy homeostasis, and inflammatory responses. We speculate that the observed reductions in body weight, glucose levels, and kidney mass may reflect broader physiological consequences of long-term neuronal dysfunction and accelerated aging processes in *Apex1* cKO mice. Notably, a previous study (Li et al., 2018)[6] reported that early postnatal deletion of Apex1 resulted in impaired growth, reduced organ size, and increased cellular senescence, further supporting a systemic impact of Apex1 deficiency. We have added this interpretation to the revised Discussion (Lines 456-463).

7. "In FigS9, it would be beneficial to show a graph with number of DEGs in the different groups for easier comparison. Are gene expression changes in Apex1 cKO rescued after CR intervention and therefore similar to WT CR mice? I would also suggest moving gene expression data to a main figure."

Response: We thank the reviewer for this helpful suggestion. In response, we have significantly revised the presentation of our microarray data to facilitate clearer cross-group comparisons and provide a more comprehensive picture of transcriptomic changes. Specifically, we have now included a Venn diagram summarizing the number of differentially expressed genes (DEGs) for key pairwise comparisons among the four experimental groups (new Fig. 5B). This summary provides a clear overview of the relative transcriptional impact of genotype and CR treatment. To further address the reviewer's insightful question about whether caloric restriction rescues the gene expression profile of Apex1 cKO mice, we examined the global transcriptional relationships between groups using principal component analysis (PCA). The resulting PCA plot (new Fig. 5A) demonstrates that Apex1 cKO AL and WT AL mice are transcriptionally distinct, while CR induces a pronounced shift in the Apex1 cKO group. Notably, the Apex1 cKO CR samples move closer to the WT CR samples in the PCA space, suggesting that CR partially ameliorates the transcriptional alterations caused by Apex1 deficiency (Fig. 5A). These findings support our conclusion that CR confers gene expression benefits even in the context of a potentially progeroid genotype. We have revised the Results, Methods, and Figure Legends accordingly, and key elements of the microarray data (originally in Supplementary Figure S9) have been moved to the main figures (the new **Figure 5**) to highlight their importance.

8. "Although interesting, the correlation analysis with human RNAseq data looks at brain regions that are not necessarily relevant for this study. How about analyzing publicly available datasets that cover the hippocampus (e.g. GTEx -Genotype-Tissue Expression Project)?"

Response: Thank you for this excellent suggestion. We have now performed analysis on the adult GTEx-Hippocampus data, which are presented in Figure S9 and Lines 691-708. Many of the changes reflect a pro-aging impact on the hippocampal transcriptome.

9. "In the discussion in line 330-334, it is not correct to state that the current study is filling a missing gap between Apex1 and brain aging. There are recent studies examining this relationship among a previous

study by the authors themselves (Zhu et al., 2022 - DOI: 10.14336/AD.2022.0331 and Li et al.,2018 – DOI: 10.1093/nar/gky326).”

Response: We thank the reviewer for this important clarification. We agree that recent studies, including our own work (Zhu et al., 2022)[7] and that of Li et al. (2018)[6], have begun to explore the role of *Apex1* in the context of brain aging and neurodegeneration. We have revised the Discussion (Lines 394-400) to better reflect the current state of the literature and to clarify that our contribution lies in extending this knowledge using a neuron-specific, aging-focused conditional knockout model to investigate the functional consequences of *Apex1* deficiency and the neuroprotective impact of caloric restriction.

10. “In Line 169-171 and Line 247, it is not correct to associate Apex1 with superior cognitive function. CR rather restores memory function in Apex1 cKO mice but not above the WT CR level.”

Response: We thank the reviewer for this helpful clarification. We agree that the original wording could be misinterpreted as suggesting that *Apex1* cKO CR mice exhibited superior cognitive performance compared to WT CR mice. Our intention was to convey that CR restored memory and cognitive function in *Apex1* cKO mice relative to their AL-fed counterparts, not that they outperformed WT mice. In revision, we describe the CR effect in Apex1 cKO mice using neutral, statistics-aligned phrasing to eliminate any wording that overstate the effect. These changes appear throughout the Results and Discussion and are consistent with the updated figures and numerical source data.

11. “The abbreviation AL is not introduced and should be used consistently in the study - AL vs Ad-lib in figures.”

Response: We thank the reviewer for pointing this out. We have now defined the abbreviation "AL" (*ad libitum*) in the text (Lines 110 and 930), and we have revised the figures to use "AL" consistently throughout the manuscript.

12. “Fig S4C - a graph legend is missing.”

Response: Thank you for catching this. We have included legends to the graphs in Fig. 4G (previous Fig. S4C).

13. “Line 280 - number of DEGs in superscript 524.”

Response: We have corrected this (Line 266).

14. “Line 300 – it should be “selected genes”.”

Response: Corrected as suggested (Line 280).

Reviewer #2

We thank Reviewer #2 for the time and effort spent reviewing our manuscript and for providing detailed and constructive suggestions. We have carefully considered all of the reviewer's comments and have revised the manuscript accordingly. Below, we provide a point-by-point response to each concern.

1. "In the present study, Wei and co-workers leveraged the conditional APE1 knockout (ko) mice under the control of the Camk2α promotor, a model they established in their previous work, to investigate whether 30% caloric restriction (CR) over 40 weeks would attenuate detrimental effects induced by conditional APE1 ko (cAPE1 ko) in a neuron specific context. *Main findings included (1) CR boosted locomotion independent of genotype, (2) CR improved spatial learning and memory independent of genotype, (3) CR partially rescued the shortening of dendritic length and reduction in branch points in CA1 neurons of cAPE1 ko mice, (4) CR elevated the CA1 dendritic spine density independent of genotype, (5) CR incremented hippocampal long-term potentiation independent of genotype*. Although I acknowledge the workload put into this study, I disagree with the authors in terms of *data interpretation and conclusion*. Furthermore, the data presentation in its current form *appears to be less ideal, difficult to interpret and confusing to some extent*. Finally, *fundamental flaws in the experimental design* exist which cannot be compensated by adding more data or re-analysis of the existing data, at least in my personal opinion. Please find my detailed comments below."

Response: We thank the reviewer for carefully reading of our manuscript and for acknowledging the effort involved in this study. While we appreciate the feedback, we respectfully disagree with several key points raised regarding the interpretation of our data and the conclusion that the effects of caloric restriction (CR) are uniformly independent of genotype. Our statistical analyses indicate that several CR-related outcomes—particularly in hippocampal structure, function, and behavior—are **genotype-dependent**. For instance, the beneficial effects of CR on spatial memory performance, dendritic complexity, and glial reactivity were more pronounced or specific in *Apex1* cKO mice. To clarify the results of those key assays, we have included a new summary table below, detailing statistically significant genotype-by-diet interactions, according to full-factorial two-way ANOVAs.

In response to the reviewer's concerns about data presentation, we have taken the following steps in the revised manuscript: 1) Reorganized the behavioral data to improve narrative and logical flow; 2) Included a statistical summary table listing all key comparisons and p-values for transparency; and 3) Expanded and clarified experimental details, including justification of the model design and genotype-specific interpretations.

We hope these revisions will address the concerns raised and provide a clearer framework for evaluating our findings. Detailed responses to each of the reviewer's specific comments are provided below.

Table 1 (Does CR have genotype-dependent effects?)

**Figure panel**	**Assay name**	**Key findings**	**Statistical details**
Fig. 1D-G	Open field test	CR significantly improves the overall performance (total distance traveled and time in the corner) in cKO mice but only had marginal effects in WT mice at 48 weeks.	**Fig. 1E** Total distance traveled (48 weeks)<br>Kruskal-Wallis ANOVA /Dunn *post hoc* p = 0.0043**<br>WT AL-cKO AL p = 0.2104<br>WT AL-WT CR p > 0.9999<br>cKO AL-cKO CR p = 0.1394<br>cKO CR-WT CR p = 0.6950<br><br>Two-tailed *t* test:<br>cKO AL-cKO CR p = 0.0331*<br><br>**Fig. 1G** Time in the corner (48 weeks)<br>Genotype x Diet interaction:<br>ANOVA p = 0.5286<br><br>Genotype effect:<br>ANOVA p = 0.6600<br><br>Diet effect:<br>ANOVA p < 0.0001***<br><br>WT AL-cKO AL p > 0.9999<br>WT AL-WT CR p = 0.0203*<br>cKO AL-cKO CR p = 0.0099**<br>cKO CR-WT CR p > 0.9999
Fig. 1J-K	Novel objective recognition test	CR significantly elevates the Preference index in cKO mice at 48 weeks, but not in WT mice.	**Fig. 1K** Preference index (48 weeks)<br>Genotype x Diet interaction:<br>ANOVA p = 0.0970<br><br>Genotype effect:<br>ANOVA p = $0.0035^{\#\#}$<br><br>Diet effect:<br>ANOVA p = 0.0016**<br><br>WT AL-cKO AL p = $0.0131^{\#}$<br>WT AL-WT CR p > 0.9999<br>cKO AL-cKO CR p = 0.0055**<br>cKO CR-WT CR p > 0.9999

Fig. 2A and B	MWM test	CR significantly improves spatial learning in cKO mice, but not in WT mice at 48 weeks.	**Fig. 2B** Latency to escape (48 weeks)<br>Genotype x Diet interaction:<br>ANOVA p = 0.0006***<br><br>Genotype effect:<br>ANOVA p = $0.0005^{\#\#\#}$<br><br>Diet effect:<br>ANOVA p < 0.0001***<br><br>WT AL-cKO AL p < $0.0001^{\#\#\#}$<br>WT AL-WT CR p > 0.9999<br>cKO AL-cKO CR p < 0.0001***<br>cKO CR-WT CR p > 0.9999
Fig. 2D and E	MWM test	CR significantly improves spatial memory in cKO mice, but not in WT mice.	**Fig. 2D** Time percentage in target quadrant (WT)<br>Age x Diet interaction:<br>ANOVA p = 0.0038**<br><br>Diet effect:<br>ANOVA p = 0.7069<br><br>Age effect:<br>ANOVA p < 0.0001***<br><br>WT CR -WT AL:<br>ANOVA p = 0.7069<br>8W: p = 0.5015<br>12W: p = 0.1995<br>17W: p > 0.9999<br>21W: p > 0.9999<br>25W: p > 0.9999<br>29W: p = 0.0052**<br>34W: p > 0.9999<br>38W: p > 0.9999<br>43W: p > 0.9999<br>48W: p > 0.9999<br>**Fig. 2D** Time percentage in target quadrant (Apex1 cKO)<br>Age x Diet interaction:<br>ANOVA p = 0.0393*<br><br>Diet effect:<br>ANOVA p < 0.0001***<br><br>Age effect:<br>ANOVA p = 0.0005***<br><br>Apex1 cKO CR-Apex1 cKO AL:<br>ANOVA p < 0.0001***<br>8W: p > 0.9999<br>12W: p > 0.9999<br>17W: p > 0.9999

			21W: p = 0.6988<br>25W: p = 0.2319<br>29W: p = 0.0046**<br>34W: p = 0.0035**<br>38W: p = 0.0009***<br>43W: p = 0.2524<br>48W: p = 0.0035**<br><br>**Fig. 2E** Time percentage in target quadrant (48 weeks)<br>Genotype x Diet interaction:<br>ANOVA p = 0.0217*<br><br>Genotype effect:<br>ANOVA p = 0.0321$^{\#}$<br><br>Diet effect:<br>ANOVA p = 0.7071<br><br>WT AL-cKO AL p = 0.0172$^{\#}$<br>WT AL-WT CR p = 0.3857<br>cKO AL-cKO CR p = 0.9109<br>cKO CR-WT CR p > 0.9999
Fig. S4F			**Fig. S4F** Times across the platform (48 weeks)<br>ANOVA p = 0.0076**<br><br>Genotype effect:<br>ANOVA p = 0.1441<br><br>Diet effect:<br>ANOVA p = 0.0005***<br><br>WT AL-cKO AL p = 0.0307$^{\#}$<br>WT AL-WT CR p > 0.9999<br>cKO AL-cKO CR p = 0.0001***<br>cKO CR-WT CR p > 0.9999
Fig. S4B			**Fig. S4B** Path efficiency (48 weeks)<br>Diet x Genotype interaction:<br>ANOVA p = 0.0106<br><br>Genotype effect:<br>ANOVA p < 0.0001<br><br>Diet effect:<br>ANOVA p = 0.0612<br><br>WT AL-cKO AL p < 0.0001$^{\#\#\#}$<br>WT AL-WT CR p > 0.9999<br>cKO AL-cKO CR p = 0.0103*

			cKO CR-WT CR p = 0.5408
Fig. 3A-F	Dendritic and spines	CR significantly improves dendritic length, branch points, and the number of intersections in cKO mice, but not in WT mice.<br><br>Fig. 3F shows that CR significantly increases the total spine length in cKO mice, but not in WT mice.	**Fig. 3B** Total dendritic length<br>Genotype x Diet interaction:<br>ANOVA $p = 0.0855$<br><br>Genotype effect:<br>ANOVA $p < 0.0001^{\#\#\#}$<br><br>Diet effect:<br>ANOVA p = 0.0245*<br><br>WT AL-cKO AL $p = 0.0007^{\#\#\#}$<br>WT AL-WT CR p > 0.9999<br>cKO AL-cKO CR p = 0.0366*<br>cKO CR-WT CR p = 0.1424<br><br>**Fig. 3B** Basal branch point<br>Genotype x Diet interaction:<br>ANOVA p = 0.0140*<br><br>Genotype effect:<br>ANOVA $p = 0.0014^{\#\#}$<br><br>Diet effect:<br>ANOVA p = 0.1178<br><br>WT AL-cKO AL $p = 0.0016^{\#\#}$<br>WT AL-WT CR p > 0.9999<br>cKO AL-cKO CR p = 0.0318*<br>cKO CR-WT CR p > 0.9999<br><br>**Fig. 3F** Total spine length<br>Genotype x Diet interaction:<br>ANOVA p = 0.0003***<br><br>Genotype effect:<br>ANOVA $p < 0.0001^{\#\#\#}$<br><br>Diet effect:<br>ANOVA p < 0.0001***<br><br>WT AL-cKO AL $p < 0.0001^{\#\#\#}$<br>WT AL-WT CR p > 0.9999<br>cKO AL-cKO CR p < 0.0001***<br>cKO CR-WT CR $p = 0.0056^{\#\#}$<br><br>**Fig. 3F** Spines/10 µm<br>Genotype x Diet interaction:<br>ANOVA p = 0.0125*<br><br>Genotype effect:

			ANOVA $p < 0.0001^{\#\#\#}$<br><br>Diet effect:<br>ANOVA p < 0.0001***<br><br>WT AL-cKO AL $p < 0.0001^{\#\#\#}$<br>WT AL-WT CR p = 0.2777<br>cKO AL-cKO CR p < 0.0001***<br>cKO CR-WT CR $p = 0.0165^{\#}$
Fig. 3G-I	8-OHdG staining	CR significantly decreases 8-OHdG^{+} neurons in cKO mice, but not in WT mice.	**Fig. 3I** 8-OHdG^{+} neurons<br>Genotype x Diet interaction:<br>ANOVA p = 0.0060<br><br>Genotype effect:<br>ANOVA $p < 0.0001^{\#\#\#}$<br><br>Diet effect:<br>ANOVA p = 0.0025**<br><br>WT AL-cKO AL $p < 0.0001^{\#\#\#}$<br>WT AL-WT CR p > 0.9999<br>cKO AL-cKO CR p = 0.0008***<br>cKO CR-WT CR p = 0.9646
Fig. 4A-F	LTP	CR significantly enhances LTP in cKO mice, but is less effective in WT mice.	**Fig. 4B** LTP (WT CR vs. WT AL)<br>Time x Diet interaction:<br>ANOVA p < 0.0001***<br><br>Diet effect:<br>ANOVA p = 0.0926<br><br>**Fig. 4C** LTP (WT CR vs. WT AL)<br>10-20 min: p = 0.0111*<br>30-40 min: p = 0.2593<br>50-60 min: p = 0.5350<br><br>**Fig. 4E** LTP (cKO CR vs. cKO AL)<br>Time x Diet interaction:<br>ANOVA p < 0.0001***<br><br>Diet effect:<br>ANOVA p = 0.0137*<br><br>**Fig. 4F** LTP (cKO CR vs. cKO AL)<br>10-20 min: p = 0.0491*<br>30-40 min: p = 0.0056**<br>50-60 min: p = 0.0235*

Fig. 4H-I	vGlu1 staining	CR significantly increases vGlu1$^+$ puncta in CA1 of both WT and Apex1 cKO mice.	**Fig. 4I** vGlu1$^+$ puncta in CA1<br>Genotype x Diet interaction:<br>ANOVA $p = 0.6889$<br><br>Genotype effect:<br>ANOVA $p = 0.2446$<br><br>Diet effect:<br>ANOVA $p = 0.0001$***<br><br>WT AL-cKO AL $p > 0.9999$<br>WT AL-WT CR $p = 0.0102$*<br>cKO AL-cKO CR $p = 0.0361$*<br>cKO CR-WT CR $p > 0.9999$
Fig. 4H-I		CR significantly increases vGlu1$^+$ puncta in CA3 of Apex1 cKO mice, but not in WT mice.	**Fig. 4I** vGlu1$^+$ puncta in CA3<br>Diet x Genotype interaction:<br>ANOVA $p = 0.8245$<br><br>Genotype effect:<br>ANOVA $p = 0.7419$<br><br>Diet effect:<br>ANOVA $p = 0.0006$***<br><br>WT AL-cKO AL $p > 0.9999$<br>WT AL-WT CR $p = 0.0713$<br>cKO AL-cKO CR $p = 0.0357$*<br>cKO CR-WT CR $p > 0.9999$
Fig. S10	GFAP staining	CR significantly decreases the levels of GFAP expression in CA1 and CA3 of cKO mice, but not in WT mice.	**Fig. S10H** GFAP$^+$ cells in CA1<br>Diet x Genotype interaction:<br>ANOVA $p = 0.0005$***<br><br>Genotype effect:<br>ANOVA $p = 0.0001^{\#\#\#}$<br><br>Diet effect:<br>ANOVA $p = 0.0001$***<br><br>WT AL-cKO AL $p < 0.0001^{\#\#\#}$<br>WT AL-WT CR $p > 0.9999$<br>cKO AL-cKO CR $p < 0.0001$***<br>cKO CR-WT CR $p > 0.9999$<br><br>**Fig. S10H** GFAP$^+$ cells in CA3<br>Diet x Genotype interaction:<br>ANOVA $p < 0.0001$***<br><br>Genotype effect:<br>ANOVA $p < 0.0001^{\#\#\#}$<br><br>Diet effect:

			ANOVA p = 0.0057**<br><br>WT AL-cKO AL p < 0.0001###<br>WT AL-WT CR p = 0.6628<br>cKO AL-cKO CR p < 0.0001***<br>cKO CR-WT CR p > 0.9999

2. "…First of all, it might be favorable to rearrange the order as well as the structure of the behavior datasets such that panels derived from one particular type of experiment are portrayed as stand-alone figures. Content wise, one could consider to start the results section by describing the outcomes of the open field test, followed by the two memory-related paradigms (MWZ and novel object recognition)."

Response: We thank the reviewer for this helpful suggestion regarding figure organization and narrative structure. We agree that starting with the open field test and then presenting the two memory-related paradigms improves the logical flow of the behavioral results. In the revised manuscript, we have reorganized the Results section accordingly and adjusted the corresponding figures to reflect this new structure (see Figures 1 and 2 and the revised text).

3. "Secondly, it could be more meaningful to show the combined data derived from wt and cAPE1 ko mice in the respective panels (alike in Fig. 3E,F or Supp. Fig. 1E), instead of the current genotype-split variant in a high number of figures and supplementary figures. The side-by-side data presentation would allow direct comparisons to visually estimate the influence and interaction of within and between factors such that the reader is not forced to jump between figure panels back and forth. As an additional advantage, the manuscript would be cleansed to a certain extent by reduction of total figure panels."

Response: We appreciate the reviewer's suggestion and agree that side-by-side data presentation can be useful in some contexts. However, in this study, we chose to present the behavioral and physiological data stratified by genotype to highlight the *genotype-specific* effects of caloric restriction (CR). As detailed in our statistical summary table (now included in the revised manuscript as Supplementary Tables S1 and S2), several CR-induced changes—particularly those related to memory, synaptic structure, and glial activation (please also see Table in this response letter)—are not uniform across genotypes but instead show genotype × diet interactions. Collapsing the data into combined panels would obscure these important distinctions and potentially mislead readers about the nature of the effects. We have, however, made efforts to streamline the figures and improve clarity where possible, and have cross-referenced related panels more explicitly in the figure legends and Results text.

4. "In the M+M section, the authors stated that either the ordinary 2-way ANOVA or a 2-way ANOVA featuring repeated measures were applied for statistical analyses. However, the analysis of a number of figure panels (for instance Fig. 3E,F or Supp. Fig. 1E) would demand for the usage of a 3-way ANOVA based on the factors age, genotype and dietary restriction. Even if the authors insist to stick with the 2-way ANOVA based statistics, it would be great to display the p-values derived from the 2-way ANOVA

analyses directly in the according figure panels to show whether a main age effect, a main genotype effect and/or an age x genotype interaction exist. In case this would not be possible due to space limitation, one could present those numbers in an organized table (e.g. in excel format) as a supplementary file."

Response: We thank the reviewer for this thoughtful comment regarding statistical presentation. In our analysis, we used two-way ANOVAs (and repeated-measures ANOVAs where appropriate) to assess the main effects and interactions of genotype and diet. While we considered adding age as a third factor, we found that in most cases, sample sizes and study design were better suited to age-stratified two-way analyses, which allowed for clear interpretation of genotype × diet interactions at specific time points. To improve transparency, we have now tables summarizing the key p-values for main effects and interactions (genotype, diet, and where applicable, age) for each relevant assay. These tables are provided as Supplementary Tables 1 & 2 and complements the updated figure legends and Results section.

5. "The general validity of the entire experimental approach needs to be questioned due to the following reasons. To estimate food intake and to calculate the daily food allotment assigned to the CR mice, the authors measured the food consumption of AL-fed mice prior to initiation of the study. However, the food intake was not re-accessed and re-adjusted for the CR animals at later time points. Since the dynamic nature of murine food intake over lifetime is well documented in numerous reports, the assumption that the food intake of a mouse remains constant over the duration of almost a year appears to be inept and represents one major flaw of the current study."

Response: We appreciate the reviewer's concern regarding the dynamic nature of food intake across the lifespan in mice. As is standard in long-term CR studies, we based the CR allotment on average food intake measured in age- and sex-matched ad libitum (AL) controls at baseline. This approach is consistent with widely accepted CR protocols used in aging research.[8,9] We acknowledge that individual food intake may change with age; however, it is not technically feasible to monitor and adjust daily intake for each individual animal over a 40-week period without introducing additional variables (e.g., single housing, stress). Our goal was to model a realistic and standardized CR regimen commonly used in the field, rather than achieve precise caloric matching at every time point. We have added a note in the Methods and Discussion sections to clarify this point and acknowledge the limitation (Lines 527-532 and 438-446). We also emphasize that the bodyweight graphs of Figure S11A demonstrate an unequivocal and consistent reduction in body mass for both CR groups—until the end of the study period. This significant loss of body mass reduces the confounding impact of age-related changes in food intake over the duration of our study.

6. "Furthermore, the authors opted to group-house AL-fed animals while CR mice were individually housed, as stated in the M+M section. Keeping the groups of animals at diverging conditions impedes a proper interpretation of the experimental outcomes given that housing condition is known to affect behavior, learning and memory."

Response: We appreciate the reviewer's point regarding the potential influence of housing conditions on behavioral outcomes. In our study, AL-fed animals were housed in pairs (no more than two per cage), which minimized social hierarchy effects and allowed for cost-effective management without overcrowding. CR animals were also housed in pairs, except during the daytime testing period, when they had to be housed individually to ensure accurate food delivery and compliance with CR protocols—an approach widely used in aging research. We acknowledge that single housing can influence behavioral phenotypes; however, caloric restriction itself has a dominating effect on activity, cognition, and physiology, as demonstrated in numerous studies where individually housed CR mice still outperform group-housed controls.

Importantly, our study design was balanced across genotype and diet within each housing condition, and we observed genotype-specific effects of CR despite any potential confounding due to housing. Nonetheless, we now explicitly acknowledge this limitation in the Discussion (Lines 418-430) and note that future studies with uniform housing across all groups would further strengthen interpretations.

7. "Behavior tests were repetitively administered based on a 4-weeks interval from 8 weeks of age onwards. In line with previous studies, animals familiar with a given test perform substantially better at subsequent occasions. Accordingly, the outcomes derived from the behavior tests rendered the ability to retain memory over a long period of time, rather than memory formation over a few days. I´m not aware that the authors made this discrimination explicitly."

Response: We thank the reviewer for this thoughtful comment. It is true that repeated exposure to the same behavioral tasks can influence performance over time, potentially reflecting memory retention or familiarity with the task structure. However, in our longitudinal design, repeated testing allowed us to track the trajectory of cognitive aging and treatment effects within individual animals, providing greater statistical power and sensitivity to detect age- and genotype-related changes. While this design may not isolate *de novo* memory formation at each time point, it does allow for meaningful assessment of long-term trends in spatial learning, memory retention, and the influence of CR. To address the reviewer's point, we have added a clarification in the revised Discussion (Lines 431-437), noting that the observed improvements in performance may reflect both memory retention and reduced cognitive decline over time, and that the repeated testing paradigm increases our confidence in the robustness and consistency of the behavioral outcomes.

8. "Throughout the entire manuscript, the authors emphasized on the validity of cAPE1 ko mice as a model of premature aging without demonstrating it clearly. The only piece of convincing evidence I recognized was the abundance of 8-OHdG in the CA1 region shown in Fig. 3K. As outlined above, the differences in MWZ performance between cAPE1 ko and wt mice arose from lack of memory retention starting from 17 weeks onwards, while similar learning curves were found when initially tested at 8 weeks of age (Fig. 1C,D). Exploratory activity in a novel environment shown as total distance moved in the open field test

did not differ between wt+AL and cAPE1 ko+AL mice, while general elevation was detected after long-term CR treatment (Fig. 2C). Analyses of dendritic length and branch points of CA1 neurons, shown in Fig. 3C,D, seem to be less appropriate given that each neuron was apparently treated as an individual sample, which artificially yielded a very high sample size. It would be more meaningful to calculate the average of each biological individual and run the statistics accordingly."

Response: We appreciate the reviewer's critical evaluation of the *Apex1* cKO model and welcome the opportunity to clarify and strengthen our rationale. While the current manuscript focuses on caloric restriction and aging-related phenotypes, the characterization of the *Apex1* cKO mouse model has been previously reported in detail in our 2022 study (Zhu et al., Aging and Disease),[7] which demonstrated early onset of cognitive decline and molecular signatures of accelerated brain aging. We have now cited and summarized this prior work in the revised Introduction to better support the validity of the model (Lines 73-81).

Regarding the dendritic morphology data (Fig. 3B, previous Fig. 3C, D), we agree with the reviewer that it is important to account for biological replicates at the animal level. In our original analysis, individual neurons were treated as technical replicates, a common approach for evaluating neuronal structure. However, to address this concern, we have now reanalyzed the data by averaging values per animal (biological replicates) and rerunning the statistical tests accordingly. Please note that the overall conclusions remain unchanged and continue to support the genotype-specific rescue of dendritic structure by CR. These updated analyses are now presented in the revised main figure.

9. "In their previous work published end 2022 (PMID36465182), density of vGlut in the cortex, CA1 and CA3 regions were significantly decreased in cAPE1 ko mice. This phenotype was not successfully reproduced in the current study (Fig. 4H, Supplementary Fig. 5B). Similarly, density of GAD was significantly decreased in cAPE1 ko mice in their 2022 publication, but was not distinguishable between wt+AL and cAPE1 ko+AL mice in the current study (Fig. 4I, Supplementary Fig. 6B). These discrepancies raise two issues. Firstly, the behavior phenotypes observed in the current study are unrelated to vGlut and GAD levels such that the underlying molecular base remains obscure. Secondly, the failure to validate phenotypes previously established in the same lab potentially indicate that either the model is volatile from batch to batch or alternatively the phenotypes of the model may vary depending on the actual age stage. Both scenarios are worrisome and don´t support the validity of the mouse model."

Response: We thank the reviewer for raising this important point. Our previous study (Zhu et al., 2022) provided detailed molecular characterization of the Apex1 cKO model and demonstrated reduced expression of vGlut and GAD in multiple brain regions. In the current study, our primary focus was to examine the effects of caloric restriction (CR) on aging-related phenotypes, and therefore the data presentation emphasizes diet-dependent changes. It is possible that differences in age, cohort, or housing conditions contributed to the variability in absolute expression levels of vGlut and GAD in AL-fed animals between studies. However, we would like to emphasize that **the ages of assessment (24 weeks in the 2022 paper versus 48 weeks in the current study) are different** and that Table 2 (below) provides evidence supporting premature aging in our model. In addition, the relative patterns remain consistent—

CR partially restored vGlut expression in CA1 and GAD expression in the cortex and hippocampus of cKO mice. Additionally, the behavioral and electrophysiological deficits we observed are consistent with impaired excitatory-inhibitory balance, which was mitigated by CR. We continue to consider the Apex1 cKO model as a valid tool for studying DNA repair deficiency and age-related neurodegeneration, and we have compiled a summary table below highlighting the core features of this model across studies.

We respectfully disagree with the reviewer's assertion that there is insufficient evidence supporting the validity of the APEX1 cKO model as a tool to study accelerated brain aging. While the primary focus of our current manuscript is on the effects of caloric restriction (CR), we acknowledge that this may have limited the visibility of genotype-specific phenotypes in the original presentation. In response to the reviewer's concern, we now include expanded comparisons between Apex1 cKO-AL and WT-AL groups across multiple behavioral, molecular, and structural endpoints. These analyses reveal clear deficits in memory retention, synaptic structure (dendritic spines) and function (LTP), oxidative DNA damage, and glial reactivity in cKO-AL mice relative to WT-AL controls, consistent with accelerated aging features. This additional presentation highlights the baseline vulnerability of the cKO model and reinforces its validity as a system for studying age-related neuronal decline. A summary of these findings is now provided in Table 2.

Table 2 (Do the Apex1 cKO show harmful effects in the current study?)

**Parameters (cKO vs. WT)**	**2022 paper, 24 wks**	**Current study, up-to-48 wks**
MWM Learning	No changes 4,5,6 M	Significant decrease, Fig. S2
MWM Memory	Significant decrease, 4-6M	Significant decrease, Fig. S2
vGlut1^{+} spots	Significant decreases in CA1, CA3, Ctx, 24W<br>t-test based on two groups	No changes in CA1 or CA3, 48W, two-way ANOVA, Fig. 4I
GAD^{+} spots	Significant decreases in CA1, 24W<br>t-test based on two groups	No changes in CA1, CA3, or Ctx, 48W, two-way ANOVA, Fig. S7
Spine number, length	Significant decreases, 24W CA1, Ctx	Significant decrease in CA1, 48W, Fig. 3
LTP (fEPSP)	Significant decreases, 24W	Significant decrease, 48W, Fig. S6
Open field	Evaluation not conducted	No difference -- Distance traveled,<br>Significant increases in time in the corner, Fig. S1<br>Significant decreases in time in the center, Fig. S1
Novel object recognition test	Evaluation not conducted	Significant decreases in preference index, Fig. S1
8-OHdG	Evaluation not conducted	Significant decrease in CA1, 48W, Fig. 3

APE1 staining	Evaluation not conducted	Significant decreases in CA1, CA3, and Ctx, 48W, Fig. S5A-D
β-Pol staining	Evaluation not conducted	No changes in CA1, CA3, or Ctx (supporting the APE1-cKO-specific model)
GFAP staining	Evaluation not conducted	Significant increases in CA1 and CA3,48W, Fig. S10

10. "Abbreviations should be unified throughout the main text of the manuscript, the figures and legends. Apex1 cKO is used in most figure panels, while APE1 cKO is also present in some figure panels. For instance in Fig. 3, both versions are included. Although I understand that the former refers to the gene and the latter refers to the protein, it might be meaningful to use Apex1 cKO exclusively given that this abbreviation was introduced by the authors in the abstract."

Response: We thank the reviewer for noting this inconsistency. We have revised the manuscript and all figure panels to use the term ***Apex1* cKO** consistently throughout, in alignment with the gene-based nomenclature introduced in the abstract and main text.

11. "The bar chart in Fig.2F does not correspond to data shown in the left and middle panel. In the left panel, the preference index of the wt+AL mice is slightly higher than the value displayed for the wt+CR mice at 48 weeks of age. The opposite is shown in the bar chart on the right. Same applies for Supplementary Fig. 2B."

Response: We apologize that the two groups were accidently flipped. We have now corrected these and other errors in the revised figures. We are immensely grateful that the reviewer helped us identify these errors.

12. "Sample size of the APE1 cKO group shown in Supplementary Fig. 1A does not align with the other panels of this figure, although the data was obtained from the same animals. While the APE1 cKO group shows 12 data points, 14 data points are indicated in the other panels."

Response: There was a data-loading error for the original Supplementary Fig. 1A. We have now corrected this error. Thank you for identifying this error as well.

13. "It is interesting that conditional knockout of APE1 in the central nervous system specifically reduced kidney weight while the brain and other peripheral organs remained unaffected. Unfortunately, the authors did not follow-up on this finding."

Response: We agree with the reviewer that the selective reduction in kidney weight is intriguing. At present, we do not have sufficient mechanistic data to interpret this finding, but we believe it may reflect systemic consequences of chronic neuronal dysfunction, as discussed in the revised manuscript (Lines

456-463), as well as the expression of CaMKII in the renal/urinary system[10-12]. We appreciate the suggestion and consider this an important direction for future investigation.

14. "The data presentation related to the microarray-based analyses could be further refined. Six groups (wt young, wt AL, wt CR, Apex1 cKO young, Apex1 cKO AL, Apex cKO CR) with unknown sample sizes and age structure have been analyzed, as stated in the M+M section. The current data presentation including only four groups prohibit any conclusion related to age (no young animals included). It remains unclear to me why the authors opted to perform multiple pairwise comparisons instead of showing the DEGs with a main effect of genotype, a main effect of CR and/or a genotype x CR interaction in a comprehensive manner, e.g. using a Venn-diagram. Among the DEGs of interest shown in Supplementary Fig. 9B, cdkn1a (encoding p21) is a prominent senescence associated gene and its transcription is elevated in the Apex1 cKO mice and further exacerbated by CR, indicating an obvious pro-aging profile by the applied dietary paradigm in this specific case."

Response: We thank the reviewer for the constructive feedback and helpful suggestions to improve the clarity of our transcriptomic analyses. In our revised manuscript (Lines 661-662), we have clarified that the analyses were performed on ***four*** experimental groups: WT AL, WT CR, Apex1 cKO AL, and Apex1 cKO CR. All samples were derived from ***aged*** animals, as our primary aim was to evaluate Apex1 deletion and CR-induced transcriptomic changes in aged mice. Although young animals were included in other experiments, transcriptomic data from these animals were not analyzed in the present study. We also wish to clarify that although the original manuscript stated n=4 per group, one sample from the Apex1 cKO AL group was excluded from the final analysis due to quality control concerns—this sample deviated substantially from its group and was removed prior to differential expression analysis. Therefore, the final dataset included 15 animals: n=3 for Apex1 cKO AL and n=4 for each of the other three groups (see Lines 677-679).

To address the reviewer's suggestion, we now provide a Venn diagram summarizing the overlap of differentially expressed genes (DEGs) across key pairwise comparisons, allowing a clearer view of the main effects of genotype and diet. This new analysis illustrates the distinct and group-specific transcriptional effects of CR in both WT and Apex1 cKO mice. Regarding the expression of Cdkn1a (encoding p21), we agree it is a well-known marker of senescence. However, we interpret its elevation in Apex1 cKO CR animals not as evidence of an exacerbated pro-aging response, but rather as part of a broader, potentially adaptive stress response triggered by Apex1 deficiency. Importantly, multiple physiological and behavioral measures presented in our study demonstrate that CR mitigates aging-related decline in Apex1 cKO mice. We have revised the Results, Methods, and figure legends to reflect the correct sample sizes and analysis details, and to better align the transcriptomic data with the observed phenotypic outcomes.

We also conducted bioinformatics analysis using publicly available human GTEx-hippocampus dataset as reviewer #1 requested (Lines 691-708). The results reveal that while the mean expression levels of several senescence associated genes such as *GADD45A*, *CDKN1B*, and *CDKN1C* are increased in older human hippocampus (Fig. S9**)**, several other senescence associated genes are decreased with aging,

including CDK4, CCNB1, TERF1, GSK3B, NBN, ELP3, and PRKCD. These results suggest that: 1) Not all senescence associated genes are subjected to upregulation with aging, and some of them could be downregulated with aging. 2) Bulk RNA-seq or microarray data do not offer information about cell type- or cell population-based alterations in gene expression. Future studies employing single-cell or single-nuclei RNA-seq and transcriptomic profiling will help address these deficiencies. One function of p21 is to block cyclin-dependent kinase complexes, which halts the cell cycle in proliferating cells with genotoxic damage. This prevents cells with damaged DNA from replicating, thereby protecting the organism as a whole, and allowing time for DNA repair. Thus, p21 is believed to regulate DNA replication fork speed and promote DNA repair, functioning to stabilize the genome and not serving only as a tumor suppressor protein[13,14].

15. "Scale bar numbers shown in Fig. 3A (400 µm, 200 µm, 100 µm) don´t align with the figure legend (50 µm, 100 µm, 50 µm)."

Response: We thank the reviewer for identifying this discrepancy. We have corrected the scale bar labels and ensured consistency between the figure and legend in the revised version of Figure 3A.

16. "In lines 141-143, the authors stated that "These memory trials were confirmed by testing other parameters, such as the latency to first entry into the target zone and the time to cross the platform [Fig. S1 C, D].". In contrast, "Time to reach the platform area during the learning phase of the water maze test." is stated in the legend of Supplementary Fig. 1C-D."

Response: We appreciate the reviewer's attention to this inconsistency. We have corrected the wording in both the main text and figure legend to ensure accurate and consistent description of the behavioral parameters presented in Supplementary Figure 4E-F (previous Fig. S1C-D).

Reviewer #3

We thank Reviewer #3 for the positive evaluation of our study and for recognizing the clarity of the manuscript, the appropriateness of the statistical analyses, and the significance of our findings on the potential therapeutic effects of caloric restriction (CR) in the context of aging and *Apex1* deficiency. We also appreciate the reviewer's thoughtful suggestions, which have helped us improve the manuscript.

1. "…one important limitation that should be addressed, is the lack of information regarding the sex of the animals used. The sex variable is not included in the experimental design or data analysis. Incorporating sex as a biological variable would significantly strengthen the study, as it could reveal potential sex-specific differences in the metabolic response to CR and/or the impact of Apex1 deficiency."

Response: We thank the reviewer for this important point. This study focused exclusively on male mice to ensure adequate statistical power, leverage the well-established body of literature on aging in males, and minimize variability associated with hormonal fluctuations in aging females. We have now clarified this in the Methods section (Line 540) and acknowledged the limitation of using a single sex in the Discussion (Lines 491-497). We fully agree that incorporating both sexes in future studies will be essential to determine whether the observed effects are sex-specific.

2. "Line 83: There is a more recent Lopez-Otin et al. paper that could be cited here, defining 12 hallmarks of aging. Perhaps it would be more suitable to cite more recent literature."

Response: We thank the reviewer for this suggestion. We have updated the citation in Line 62 to include the more recent 2023 review by López-Otín et al. defining the 12 hallmarks of aging[15].

3. "Line 126: I think it should be „Apex1 cKO CR mice displayed faster swim speeds than cKO AL and WT CR" – as it is labeled on Fig S1 B."

Response: Thank you for catching this. We have corrected this sentence in Lines 150-151 to match the data shown in Fig. S4D (previous Fig. S1B).

4. "Figure 1 C, D: please add 'day' to indicate what the numbers 1-4 refer to, as in Fig S1 E, to make it more clear."

Response: We agree and have revised Figure 2A (previous Fig. 1 C and D) as suggested.

5. "Figure 1 F, G: X-axis could be just labeled Age/weeks, no need for additional line."

Response: We have revised Figure 1 and Figure 2 as the reviewer suggested.

6. "Lines 159-160: Please rewrite to make it clear that the effect that waned at 48 weeks is between cKO AL and CR mice, not between cKO CR and WT AL (it makes no sense to compare those two)."

Response: We agree and have revised the text as the reviewer suggested in Lines 134-137: "WT CR and *Apex1* cKO CR mice both spent more time exploring the center region compared to their respective AL controls (Fig. S3A-B). However, the effect between cKO CR and cKO AL waned at 48 weeks, while the difference between WT CR and WT AL persisted (Fig. S3A-B)."

7. "Figure 2: label X axis Age/weeks and consider re-labeling every panel in C, D with different letter (so to have 6 panels labeled, instead of just 2)."

Response: Revised as suggested (now Fig. 1D-G).

8. “Figure S2: label X axis Age/weeks.”

Response: Done as suggested (now Fig. S3).

9. “Figure 3F: it is not clear to which groups statistical significance refers to.”

Response: We apologize for the confusion. We have revised the figure legend (Lines 980-982) to explicitly state the pairwise comparisons for which statistical significance is indicated in Figure 3D (previous Fig. 3F).

10. “375-377: please state the paper you are referring to as Author, et al., or put the reference number in that sentence to make it more clear.”

Response: Thank you for the suggestion. In revision, we removed the “UW vs. NIA” sentence and replaced it with a streamlined, fully cited Discussion that (i) references longitudinal primate CR studies and (ii) adds a translational note from the CALERIE trial showing slowed biological aging with ~25% CR (Discussion, Lines 484-490). As the original sentence no longer appears, the citation request is resolved.

11. “Discussion: Please state the limitations of the study - biological sex of the animals used.”

Response: As noted above, we have added the following statement to the Discussion section (Lines 491-497) acknowledging the limitation of using only male mice and the need to assess sex differences in future studies: “The present study focused exclusively on male mice to ensure sufficient statistical power, draw on the extensive aging literature in males, and minimize variability from hormonal fluctuations in females. While this approach was appropriate for our initial investigation, the lack of female subjects remains a limitation. Future studies will be needed to assess whether the effects of CR and *Apex1* deficiency are consistent across sexes or show sex-specific differences. In this context, it is worth noting that no key sex differences were identified for the aforementioned blood DNA methylation analysis of the CALERIE trial subjects [16].”

References

1. Chaix, A. & Panda, S. Ketone Bodies Signal Opportunistic Food-Seeking Activity. *Trends Endocrinol Metab* **27**, 350–352 (2016).

2. Mistlberger, R.E. Neurobiology of food anticipatory circadian rhythms. *Physiol Behav* **104**, 535–545 (2011).

3. Pankevich, D.E., Teegarden, S.L., Hedin, A.D., Jensen, C.L. & Bale, T.L. Caloric restriction experience reprograms stress and orexigenic pathways and promotes binge eating. *J Neurosci* **30**, 16399–16407 (2010).

4. Hutchinson, E. Systems neuroscience: the stress of dieting. *Nat Rev Neurosci* **12**, 65 (2011).

5. Stetler, R.A.*, et al.* APE1/Ref-1 facilitates recovery of gray and white matter and neurological function after mild stroke injury. *Proc Natl Acad Sci U S A* **113**, E3558–3567 (2016).

6. Li, M.*, et al.* APE1 deficiency promotes cellular senescence and premature aging features. *Nucleic Acids Res* **46**, 5664–5677 (2018).

7. Zhu, L.*, et al.* Neuron-targeted Knockout of APE1 Forces Premature Cognitive Impairment and Synaptic Dysfunction in Adult Mice. *Aging Dis* **13**, 1862–1874 (2022).

8. Colman, R.J.*, et al.* Caloric restriction delays disease onset and mortality in rhesus monkeys. *Science* **325**, 201–204 (2009).

9. Turturro, A.*, et al.* Growth curves and survival characteristics of the animals used in the Biomarkers of Aging Program. *J Gerontol A Biol Sci Med Sci* **54**, B492–501 (1999).

10. B6.Cg-Tg(Camk2a-cre)T29-1Stl/J. https://www.jax.org/strain/005359.

11. Hanley, R.M., Shenolikar, S., Pollack, J., Steplock, D. & Weinman, E.J. Identification of calcium-calmodulin multifunctional protein kinase II in rabbit kidney. *Kidney Int* **38**, 63–66 (1990).

12. Mikhailov, A.V., Liu, Y., Cheng, H.J., Lin, J.J. & Cheng, C.P. Calmodulin-dependent protein kinase II activation promotes kidney mesangial expansion in streptozotocin-induced diabetic mice. *Heliyon* **8**, e11653 (2022).

13. Dutto, I., Tillhon, M., Cazzalini, O., Stivala, L.A. & Prosperi, E. Biology of the cell cycle inhibitor p21(CDKN1A): molecular mechanisms and relevance in chemical toxicology. *Arch Toxicol* **89**, 155–178 (2015).

14. Ticli, G., Cazzalini, O., Stivala, L.A. & Prosperi, E. Revisiting the Function of p21(CDKN1A) in DNA Repair: The Influence of Protein Interactions and Stability. *Int J Mol Sci* **23**(2022).

15. Lopez-Otin, C., Blasco, M.A., Partridge, L., Serrano, M. & Kroemer, G. Hallmarks of aging: An expanding universe. *Cell* **186**, 243–278 (2023).

16. Waziry, R.*, et al.* Effect of long-term caloric restriction on DNA methylation measures of biological aging in healthy adults from the CALERIE trial. *Nat Aging* **3**, 248–257 (2023).

The authors thank **Reviewer 2** for their careful evaluation of our revised manuscript and for the insightful and constructive comments that have further strengthened the study. We have revised the manuscript accordingly, and all changes are highlighted in blue in the revised version. Below, we provide point-by-point responses to **Reviewer 2**'s remaining comments. We hope these revisions satisfactorily address the concerns raised.

To improve clarity and accessibility of the statistical results, we have added a new condensed summary table (Table S1) that highlights outcomes showing significant genotype × diet interactions across figures. All statistical values reported in this condensed table are derived directly from, and fully consistent with, the comprehensive statistical tables (Tables S2 and S3) already included in the manuscript; no additional analyses were performed. The purpose of this condensed table is to provide readers with a high-level overview of interaction effects, while preserving the full statistical reporting in the comprehensive tables and the detailed statistics provided in the Supplementary Information. We believe this addition improves readability without altering the underlying results or conclusions.

Point 1:

"Based on the data presented in the original manuscript, I interpreted the effects induced by CR as a mixture of genotype-independent (e.g. locomotion, learning and memory, CA1 dendritic spine density, hippocampal LTP) and genotype-dependent (e.g. rescued shortening of dendritic length and reduction in branch points in CA1 neurons) phenotypes. The authors obviously disagree in this regard and emphasize that the genotype-dependent outcomes are more prominent. Although I understand that this discussion may rely on individual perspective, such that right or wrong simply does not exist, I would like to explain my view of the data by walking through all relevant panels."

Response: We thank the reviewer for this careful and balanced assessment of the data. We appreciate the reviewer's detailed evaluation of genotype-independent versus genotype-dependent effects and address each point below to clarify how we interpret these findings.

"In the open field test (formerly Fig. 2A-D, now Fig 1B-G), CR significantly elevates exploratory locomotion in both WT and Apex1 cKO mice. At 48 weeks of age, total distance traveled is higher (Fig. 1E) and the time spent in the corner regions is decreased in the CR groups, independent of genotype (Fig. 1G). In Table 1 / Fig. 1E, the parameter "Total distance traveled (48 weeks)" shows a non-significant comparison between cKO-AL vs. cKO-CR (p=0.1394) after posthoc correction. However, in Fig. 1E, the authors depicted a statistically significant outcome (p=0.0331) derived from a separate two-tailed t-test, which is not legitimate. Therefore, the overall CR effect on exploratory locomotion appears to be genotype-independent."

Response: We thank the reviewer for carefully examining the statistical analyses of the open field data and for pointing out this inconsistency. We agree that interpretation of exploratory locomotion should be based on the full-factorial two-way ANOVA rather than on individual pairwise t-tests. In the revised manuscript, the two-way ANOVA indicates a significant main effect of diet, but no significant genotype × diet interaction for total distance traveled or time spent in corner regions at 48 weeks. Accordingly, the effect of CR on exploratory locomotion is best interpreted as genotype-independent. We have revised the Results (Lines 132-156), Fig. 1E, and statistical annotations to remove the separate t-test-derived p-value and now report only the ANOVA-based results.

In the Morris water maze test (formerly Fig. 1, now Fig. 2), CR significantly improves escape latency in both wt and Apex1 cKO mice (Fig. 2A). The authors might argue that the magnitude of change at 48 weeks of age is substantially greater in the Apex1 cKO mice than in WT animals – which is true. However, this might be explained by a ceiling effect, because the performance of the mice has already reached its optimum after numerous rounds of training and further reductions of escape latency to less than a few seconds appear less likely. As shown in the bottom panels of Fig. 2C (48 weeks old), WT animals - with or without CR treatment - head straight toward the platform zone without hesitation once they enter the test arena. Regarding quadrant occupancy during the probe trial shown in Fig. 2D, I admit that the outcome of this measure resembles a true interaction between CR and genotype. In the lower panel of Fig. 2E (48 weeks old), statistical significance is depicted for the comparisons WT+AL vs. cKO-AL (###) and cKO-AK vs. cKO-CR (**). However, as shown in Table 1, the comparison WT-AL vs. cKO-AL yielded a p-value of 0.0172 (#) and the comparison cKO-AK vs. cKO-CR failed to reach statistical significance (p=0.9109). Please correct Fig. 2E accordingly.

Response: We thank the reviewer for the careful re-examination of the Morris water maze data and for raising concerns regarding the statistical annotations in Fig. 2E. Upon re-review of the statistical analyses, we confirm that the annotations shown in Fig. 2E (48 weeks, bottom panel) are correct as presented. Specifically, at 48 weeks of age, the comparison between WT AL and *Apex1* cKO AL yielded p = 0.0002 (###), and the comparison between *Apex1* cKO AL and *Apex1* cKO CR yielded p = 0.0018 (**), consistent with the annotations displayed in the figure. The p-value of 0.0172 (#) cited by the reviewer corresponds to the comparison between WT AL vs. *Apex1* cKO AL at 8 weeks of age, not 48 weeks. To minimize potential confusion, we have highlighted the relevant entries in the comprehensive statistical table for clarity. We appreciate the opportunity to clarify this point and have ensured that the figure, table, and accompanying text are fully aligned.

Regarding escape latency, we agree that CR improved learning performance in both WT and *Apex1* cKO mice. As the reviewer notes, performance in WT mice approached a ceiling after repeated training, which may limit the observable magnitude of CR-induced improvement at later ages. We have revised the Results text (Lines 177-183) to clarify this point and to emphasize that genotype-dependent effects are most evident in memory retention measures during the probe trials rather than escape latency alone.

“In the original submission, total spine density was significantly increased by CR, independent of genotype (formerly Fig. 3H right panel, now Fig. 3F right panel). After applying the 2-way ANOVA, the revised data indicates a genotype × diet interaction while the main diet effect is not significant ($p=0.0926$). Based on the statistical outcome, I agree with the authors that CR differentially affects this trait in a genotype-dependent manner.”

Response: We thank the reviewer for this careful statistical evaluation and for acknowledging that the revised two-way ANOVA supports a genotype x diet interaction for total spine density. We agree that, based on the updated analysis, this phenotype is best interpreted as a genotype-dependent response to CR.

“Hippocampal LTP is another case in which a disagreement exists. Based on the data enclosed in the original submission (formerly Fig. 4A-F), the fEPSP slope was modified by CR in both WT and Apex1 cKO mice to a similar extent, leading to my interpretation that the effect was genotype-independent. Consistent with my observation, in lines 224-226 of the original submission, the authors stated that “The hippocampal slices from 48-week-old WT CR mice showed superior LTP, with higher fEPSPs slopes than WT AL mice [Fig. 4A-C].” Curiously, in the revised manuscript, the fEPSP slope of the WT-AL mice in Fig. 4B substantially differs from the graph shown in the original submission, whereas the other fEPSP slopes remained unmodified. The marked difference in the fEPSP slopes between WT-AL and WT-CR animals, shown in Fig. 4C of the original submission, disappeared and became statistically non-significant in the revised manuscript. This data replacement demands a logical explanation.”

Response: We thank the reviewer for carefully examining the LTP data and for raising this important point regarding differences between the original (Figure 4C) and revised (Figure 4B) figures. We agree that clarification is warranted. During the revision process, we conducted a rigorous review of all raw data, reanalyzed all data for presentation, and reconstructed the figures accordingly. In doing so, we identified three WT AL hippocampal slice recordings that did not meet our predefined LTP inclusion criteria. Although LTP was initially induced in these slices, the potentiation failed to be maintained at ≥130% of baseline for 60 minutes following high-frequency stimulation (HFS). Specifically, samples #1 and #2 decayed below threshold within 10 minutes post-HFS, and sample #3 decayed at ~27.5 minutes.

Following consultation with an electrophysiology expert (Dr. W Zhang, co-first author), these three recordings were excluded from the revised analysis. As a result, the WT AL sample size was reduced from n=10 to n=7, which accounts for the change in the WT AL fEPSP slope and the loss of statistical significance between WT AL and WT CR groups in the revised manuscript. All other groups and recordings remained unchanged.

The figures below show Figure 4B and C (original submission, WT AL, n=10) that included the 3 samples marked by blue circles that failed to meet our inclusion criteria, a graph showing the three unqualified samples only, and the revised Figure 4B (WT AL, n=7) after we removed those 3 samples for the resubmission. For transparency, we have included the raw data for the 3 unqualified samples in the table below (LTP decayed below the 130% threshold is highlighted in yellow). We have also updated the Methods section (Lines 666-670) to explicitly define the LTP inclusion/exclusion criteria. We have retained all revised analyses based on uniformly applied standards, and we believe this approach improves the rigor and reproducibility of the electrophysiological findings.

Figure 4B and C in Original submission

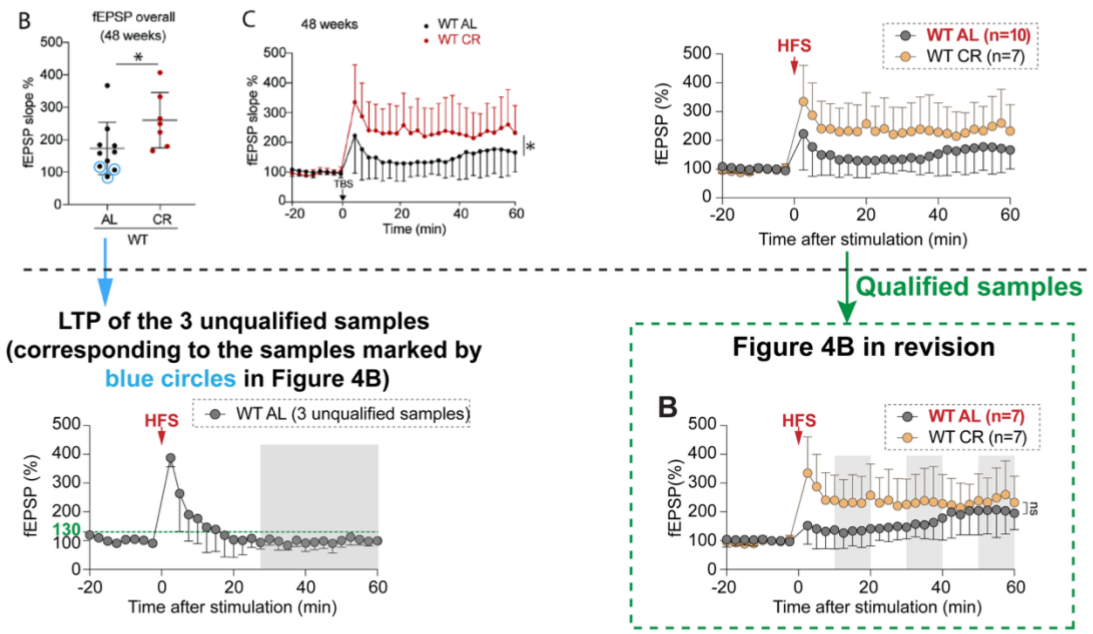

	Time after stimulation (min)	WT AL (3 excluded samples)		
		Excluded No. 1	Excluded No.2	Excluded No.3
	-20	119.7674761	123.2883778	115.4667078
	-17.5	103.7866165	107.9203739	118.0681422
	-15	100.3294959	106.1344661	89.28164842
	-12.5	93.55890117	89.42907064	88.70638067
	-10	103.1341023	94.23234791	112.7458968
	-7.5	106.7811943	103.9996907	103.6977265
	-5	107.9615167	95.55660403	99.5629548
Before HFS	-2.5	82.1231867	106.2113574	83.99342181

	2.5	373.3709193	422.7437018	366.7069931
	5	175.3478889	199.7002808	415.1928045
	7.5	135.0318484	139.2281381	295.9512632
	10	123.9767227	129.2660914	277.4670559
	12.5	91.48478288	99.90789144	247.8463584
	15	98.31006968	91.07558909	227.077764
	17.5	79.93578179	70.73473051	204.8310463
	20	79.54557438	57.54379039	172.6016509
	22.5	85.0083354	60.92114498	156.4938868
	25	92.68167729	86.78996728	143.7109284
	27.5	82.53100305	78.02030762	120.2090626
	30	101.4016384	86.67399136	127.6163325
	32.5	113.6575219	62.44144283	117.6450745
	35	87.06651443	66.28946561	98.18347294
	37.5	102.3782352	78.81748394	122.1719416
	40	99.14372819	59.18100211	116.1135676
	42.5	102.4202455	65.57892415	115.6736642
	45	119.9730655	70.32868983	105.0742061
	47.5	128.7028793	73.65037915	68.61275993
	50	126.8929996	85.12388224	88.80179627
	52.5	102.4225643	90.98266875	143.5832513
	55	118.0158117	82.50760804	111.5382067
	57.5	100.4936065	80.22809384	112.0993495
After HFS	60	104.9043393	89.62818469	103.1231523

Point 5:

"I questioned the validity of the CR treatment since a highly unusual CR protocol (group housing combined with determination of food consumption only once prior to initiation of CR) was used in this study. But the authors disagree and claim that their experimental design represents the standard approach in long-term CR studies. Furthermore, the technical feasibility of dynamically adjusting food intake was questioned by the authors and two related publications were cited, which ironically contradict the authors´ viewpoint. For instance, the Turturro et al. study, referenced by the authors, stated that "Animals were housed individually." and "Food consumption was monitored weekly for a cohort of animals. These consumptions were used to determine the ration to be fed to the CR groups in the studies." – which is precisely how a long-term CR paradigm should be administered in mice.

As shown in previous studies, food consumption in mice gradually increases from the juvenile stage to middle age, making continuous adjustment of the food allotment a crucial feature of any long-term CR study when initiated at young age. Determining food consumption only once prior to CR administration at young age bears the risk of underestimating the degree of CR at later ages. Therefore, I suggest that the authors acknowledge this limitation in their experimental design by adding a clear statement in the Discussion section noting that the CR protocol applied in the current study differs from the standard approach and the results should also be interpreted accordingly."

Response: We thank the reviewer for this thoughtful and detailed critique of our caloric restriction (CR) protocol. We agree that continuous reassessment and adjustment of food intake, typically achieved through individual housing and repeated intake measurements, represents a widely used approach in long-term CR studies, including those cited by the reviewer (e.g., Turturro et al., 1999). In the present study, we chose a group-housed CR paradigm in which food allotment was determined based on baseline intake of age- and sex-matched AL-fed controls. This design was implemented to minimize stress associated with long-term individual housing and to maintain stable social conditions across the lifespan. We acknowledge, however, that this approach differs from protocols that dynamically adjust caloric intake over time and may underestimate the degree of restriction at later ages due to age-dependent increases in food consumption.

In response to the reviewer's suggestion, we have now added an explicit statement in the Discussion (Lines 455-481) acknowledging this limitation and clarifying that our CR regimen differs from standard dynamically adjusted protocols. We emphasize that, despite this limitation, CR animals exhibited a sustained and significant reduction in body weight throughout the study (Fig. S11A), supporting the presence of a biologically meaningful caloric deficit. Nonetheless, we agree that future studies incorporating longitudinal intake monitoring will be important to refine the CR paradigm and to assess how protocol differences influence aging-related outcomes.

Point 9:

"I appreciate that the authors provided additional evidence supporting the validity of Apex1 cKO as a valuable tool to study brain aging. On the other hand, simply referring to differences in the age of assessment does not convincingly explain the failure to reproduce changes in vGlut and GAD expression levels upon conditional Apex1 deletion between studies, especially since they were conducted by the same lab. Additionally, the authors speculate whether "differences in age, cohort, or housing conditions" might contribute to the discrepancies observed between studies. Such volatility to potential batch effects may complicate the data interpretation and impede reliable reproduction of phenotypes. I suggest that the authors add a sentence to the Discussion stating that – in contrast to their previous study (Zhu et al., 2022) – expression levels of vGlut and GAD do

not differ between WT and Apex1 cKO mice at 48 weeks of age and that this observation warrants further investigation."

Response: We thank the reviewer for this constructive suggestion and agree that the difference in vGlut and GAD expression between our prior study (Zhu et al., 2022) and the current work warrants explicit clarification. As suggested, we have now added a sentence to the Discussion (Lines 482-485) stating that, in contrast to our earlier findings, vGlut and GAD expression levels do not significantly differ between WT and Apex1 cKO mice at 48 weeks of age. We further note that this age-dependent divergence merits additional investigation.

Importantly, while these molecular readouts differed between studies, multiple independent endpoints including behavior, synaptic structure and function, oxidative DNA damage, and glial activation consistently demonstrate accelerated aging phenotypes in Apex1 cKO mice. We therefore interpret the current findings as indicating that some synaptic marker changes may be age- or context-dependent, rather than reflecting instability of the model itself. We appreciate the reviewer's suggestion, which has improved the clarity and transparency of the manuscript.
